# The risk of perinatal mortality following short inter-pregnancy intervals—insights from 692 402 pregnancies in 113 Demographic and Health Surveys from 46 countries: a population-based analysis

**DOI:** 10.1016/S2214-109X(23)00359-5

**Published:** 2023-09-19

**Authors:** Mohamed M Ali, Saverio Bellizzi, Iqbal H Shah

**Affiliations:** a UNDP-UNFPA-UNICEF-WHO-World Bank Special Programme of Research, Development and Research Training in Human Reproduction, Department of Sexual and Reproductive Health and Research, World Health Organization, Geneva, Switzerland; b WHO Country Office, Amman, Jordan; c Department of Global Health and Population, Harvard T H Chan School of Public Health, Boston, MA, USA

## Abstract

**Background:**

Inter-pregnancy interval has been identified as a potentially modifiable risk factor to improve perinatal outcomes. We examined the WHO recommended interval of at least 24 months after a livebirth to next pregnancy, and its recommendation of waiting for at least 6 months after a pregnancy loss to improve subsequent pregnancy outcomes. We aimed to estimate the association between inter-pregnancy interval and perinatal mortality using the Demographic and Health Survey reproductive and contraceptive calendar.

**Methods:**

For this population-based analysis, we extracted data for pregnancies with gestational age and pregnancy outcomes from 113 publicly available Demographic and Health Surveys conducted between 2000 and 2022 in 46 countries that included a reproductive or contraceptive calendar module. The primary outcome was perinatal mortality (stillbirth and early neonatal death) while the inter-pregnancy interval was the exposure of interest, grouped into categories of less than 6 months, 6–11 months, 12–17 months, 18–23 months, and 24–59 months. The analysis was stratified by preceding pregnancy outcome (livebirths, stillbirths, or abortions). The Kaplan-Meier method and Cox proportional hazard model were used to calculate the cumulative probability of perinatal mortality and the hazard ratios (HRs).

**Findings:**

The analysis sample comprised of 692 402 pregnancies contributed by 570 145 women with a mean age of 28·4 years (SD 5·96). The overall HR of perinatal death was 2·72 (95% CI 2·52–2·93) times higher for an inter-pregnancy interval of less than 6 months compared with the WHO recommended optimal waiting time of 18–23 months following a livebirth. Overall HRs followed a context-related pattern, with the highest ratio of 2·95 (95% CI 2·67–3·25) in sub-Saharan Africa and the lowest of 1·98 (1·47–2·66) in north Africa, west Asia, and Europe. Inter-pregnancy intervals of less than 3 months, 6 months, and 12 months following stillbirth or abortion (spontaneous or induced) do not pose a higher risk for perinatal death in subsequent pregnancy.

**Interpretation:**

Our study reaffirms the WHO recommendation on optimal interval between the last livebirth and the next pregnancy of at least 24 months and avoiding pregnancy before 18 months. However, our analysis does not support the WHO recommendation of delaying the next pregnancy for at least 6 months after a pregnancy loss for improved perinatal survival.

**Funding:**

None.

## Introduction

Perinatal deaths, defined as fetal deaths occurring after 28 weeks of gestation (stillbirths) and deaths among livebirths within the first 7 days of life (early neonatal deaths), decreased from 5·7 million in 2000 to 4·1 million in 2015 worldwide.^1^

Inter-pregnancy interval, or the time from birth or termination of pregnancy to conception of the next pregnancy, has been identified as a potentially modifiable risk factor linked to adverse perinatal outcomes.^2^ The inter-pregnancy interval can specifically affect perinatal outcomes such as premature rupture of membranes, postpartum haemorrhage, pre-eclampsia, low birthweight, preterm birth, and small for gestational age.^3^

Although a long inter-pregnancy interval might not be modifiable, a short inter-pregnancy interval can be more easily modified by using contraceptives. Woman-centred family planning counselling would enable each woman to identify a method that is acceptable and commensurate with her future childbearing intentions; such an approach must be coupled with contraceptive accessibility and adequate knowledge by both health-care providers and women.^4^

**Research in context**

**Evidence before this study**

After assessing the evidence synthesised in seven commissioned papers, a Technical Consultation organised by WHO in 2005 concluded that: after a livebirth, the recommended interval before attempting the next pregnancy is at least 24 months to reduce the risk of adverse maternal, perinatal and infant outcomes, and after a miscarriage or induced abortion, the recommended minimum interval to the next pregnancy is at least 6 months to reduce risks of adverse maternal and perinatal outcomes. Additional research since the consultation generally supported the first recommendation but noted no benefit of delaying the next pregnancy by 6 months after a miscarriage or induced abortion. As put forward by Klebanoff, the inter-pregnancy interval might be less important than previously assumed among women whose most recent pregnancy ended in a stillbirth, at least for women in high-income regions. Swaminathan and colleagues reported an increased risk of stillbirth associated with an inter-pregnancy interval of less than 6 months following a livebirth or stillbirth. Our research set out to examine the risk of perinatal mortality following short inter-pregnancy intervals after a livebirth or pregnancy loss.

**Added value of this study**

To our knowledge, we conducted for the first time the largest study using a time-to-event approach to assess the effect of inter-pregnancy interval on perinatal mortality, thus providing additional evidence and a robust measure of association. Specifically, we explored the association between different inter-pregnancy intervals and perinatal mortality using the Kaplan-Meier method and Cox proportional hazard model stratified by different outcomes of the preceding pregnancy. The major findings from 113 Demographic and Health Surveys in 46 countries confirmed that the hazard ratio (HR) of perinatal mortality is more than doubled (HR 2·72, 95% CI 2·52–2·93) following a shorter inter-pregnancy interval of less than 6 months after a livebirth compared with an inter-pregnancy interval of 18–23 months. Conversely, the point estimates of inter-pregnancy intervals shorter than 3 months (HR 0·90, 95% CI 0·24–3·31), 6 months (1·17, 0·91–1·50), or 12 months (0·98, 0·73–1·32) after a stillbirth or abortion (3 months: HR 1·06, 95% CI 0·88–1·29; 6 months: 1·07, 0·96–1·19; 12 months: 0·89, 0·78–1·02) are unlikely to lead to higher perinatal mortality of subsequent pregnancy.Our analysis also revealed context-related patterns for the risk of adverse perinatal mortality in the case of short inter-pregnancy interval after a livebirth with the highest HRs in sub-Saharan Africa (HR 2·95, 95% CI 2·67–3·25), compared with countries in Asia (2·47, 2·23–2·74) and the Caribbean and Latin America (2·32, 1·74–3·10), whereas the lowest was seen in north Africa, west Asia and Europe (1·98, 1·47–2·66). This is a crucial piece to reconcile previous research indicating a different role of pregnancy intervals on perinatal mortality when comparing high-income with middle-income or low-income settings. Additionally, it reinforces the essential role of other risk factors, such as lifestyle, in the association between inter-pregnancy interval and perinatal survival.

**Implications of all the available evidence**

Our study reaffirms the 2005 WHO recommendation on optimal interval between the last livebirth and the next pregnancy of at least 24 months. However, we found no beneficial effect of the WHO recommended interval of 6 months from a pregnancy loss to the next pregnancy or longer inter-pregnancy interval on the subsequent pregnancy risk of perinatal mortality. In low-income and middle-income countries (LMICs), which account for most of the perinatal deaths, most of these deaths can be averted through optimal birth spacing by contraceptive use. Indeed, such a behavioural change is easier to achieve than improving socioeconomic status or other biological determinants. Women with a non-livebirth might be advised to attempt the next pregnancy when they feel ready. We deem these results to, despite some limitations related to the magnitude of associations, complement accumulated evidence by end of 2022 with major programmatic and policy implications for LMICs, in terms of confirmatory support to WHO recommendation on interval from previous livebirth to next pregnancy and, not supporting the WHO recommendation of delaying the next pregnancy for at least 6 months after a pregnancy loss for improved perinatal survival.

WHO recommends that women wait at least 2 years after a livebirth and 6 months after a pregnancy loss before attempting to conceive again to reduce the risk of adverse maternal and perinatal outcomes.^5^ The latter part of recommendation on interval from a pregnancy loss is inconsistent as an increasing inter-pregnancy interval after a pregnancy loss does not always appear to improve subsequent pregnancy outcomes.^6^ Evidence from studies investigating the influence of inter-pregnancy interval on perinatal outcomes, including inter-pregnancy interval after a pregnancy loss, is conflicting, with some evidence showing adverse effects on birth outcomes whereas others indicating the need of additional research because of weak or inconsistent results, or both.^7^

Epidemiological studies have shown that both short and very long inter-pregnancy intervals could lead to negative perinatal outcomes; however, maternal characteristics and lifestyle and outcome of the previous pregnancy might have confounded the association between short inter-pregnancy interval and the risk of a subsequent adverse perinatal outcome in several observational studies.^8^, ^9^ The issue of unmeasured confounders has been addressed by a large-scale cohort study using a matched-sibling design, which indicated that mothers with a short (<6 months) or long (≥36 months) inter-pregnancy interval had greater odds of adverse birth outcomes.^10^ However, authors underlined how these results might not apply to women who had experienced a miscarriage or stillbirth between deliveries.

A study of women from 58 low-income and middle-income countries (LMICs) based on the Demographic and Health Surveys did not find a consistent reduced risk of stillbirth for intervals greater than 24 months or an elevated risk of stillbirth for intervals of less than 6 months after a previous stillbirth.^11^ Such findings reinforce the need for additional evidence to inform guidelines for maternal and child health and family planning programmes.

In this study, we aimed to estimate the association between inter-pregnancy interval and perinatal mortality using the Demographic and Health Survey reproductive calendar, which allows measuring the intervals between pregnancy resolution (livebirth or pregnancy loss) and the beginning of the following pregnancy (the index pregnancy), stratified by preceding pregnancy outcome. We have used a large database of 692 402 pregnancies and examined perinatal mortality rather than stillbirth that is known to be underestimated.^12^ In addition, we have applied a time-to-event analytical approach that is more appropriate to examine the effect of the inter-pregnancy interval on perinatal mortality with right-censored data compared with a generalised linear model.

## Methods

### Study design and participants

We performed a population-based analysis using all publicly available data from the Demographic and Health Survey Program conducted from 2000 to 2022 and included a reproductive or contraceptive calendar module.^13^ The calendar module included a month-by-month complete history of the reproduction of women for a period of between 5 years and 7 years before the survey. In all surveys, the period covered by the calendar included the months up to the month of the interview, plus the 5 calendar years preceding the year of the interview. This study is a secondary analysis of publicly available anonymised data, and no ethical approval was required.

### Procedures

We extracted data of pregnancies with gestational age (in months), pregnancy outcomes (livebirth or pregnancy loss), and current pregnancy from the calendar. We calculated inter-pregnancy interval between pregnancy ended in the calendar and pregnancy that was conceived in the calendar (minimum two pregnancies) regardless of the outcome of the preceding pregnancy, and excluded pregnancies with no preceding pregnancy (hence no inter-pregnancy interval), and not at risk of perinatal death (ie, with gestational age of less than 7 months). The survey data were collected by national institutions.

As the calendar length varies between surveys, we retained pregnancies that were conceived and resulted in livebirth or pregnancy loss within the analysis period, which is defined as the period between month 7 and month 66 before the survey month. Livebirths were linked with the corresponding births (including multiple births) in the birth history module to obtain the survival status, current age, and age at death (in days, months, and years). Livebirths that occurred in the month of the interview were also retained. Surveys were stratified by the geographical region (sub-Saharan Africa; north Africa, west Asia, and Europe; central, south and southeast Asia; and Latin America and the Caribbean), according to the Demographic and Health Survey grouping**.**

### Outcomes and risk factor

The primary outcome was perinatal mortality. We defined stillbirth as a pregnancy loss with gestational age of at least 7 months, in which gestational age was ascertained on a monthly basis and is also aligned with the WHO definition of stillbirth—a baby who dies after 28 weeks of pregnancy, but before or during birth.^14^

We used an inter-pregnancy interval as our exposure of interest, defined as the number of months from the end of a pregnancy to the start of the subsequent pregnancy (the index pregnancy). The inter-pregnancy interval was grouped into categories of fewer than 6, 6–11, 12–17, 18–23, and 24–59 months. We also extracted relevant covariates: household wealth (index factor score was grouped into tertiles as poor, middle, and rich); urban-rural residence of women; women's highest level of education (grouped into no education, primary, and secondary or more); and age at conception of the index pregnancy in years (<25 years, 25–29 years, and ≥30 years), and for the index pregnancy, the gestational age and outcome of the preceding pregnancy and its gestational age. We created a pregnancy-based file rather than a woman-based file retaining the characteristics of the household and of the women as well as the index pregnancy characteristics.

### Statistical analysis

We used time-to-event analysis for real cohort, to calculate the cumulative probability of perinatal mortality [1-S(t)] using Kaplan-Meier (Product Limit) and the Cox proportional hazard model for survey data to estimate the hazard ratios (HRs) that take into account the survey stratified multistage sampling design (households were nested within a cluster), and the normalised weights.^15^ As the data were time to event measured in months and days, we could either use the actuarial life-table method or Kaplan-Meier (Product Limit) approach. Given that we have used a discrete time interval, the calculations of the survival probabilities are identical in both approaches. The underlying time at risk is measured in months for current pregnancies and stillbirths and in days for livebirths. Current pregnancies enter the analysis at month 7 and are censored at their gestational age. Livebirths are followed until the end of the perinatal period (day 6) and births that survived the perinatal period are censored at day 6.

As women could report more than one pregnancy, this reporting added an additional level to the pregnancy-based file (four levels [pregnancies were nested within women, and women are nested within household and households are nested within clusters] pregnancies are nested within women; women are nested within clusters). We collapsed the household level into the woman level due to fewer women per household, except for Senegal and Mali, where they used a compound that included the dwellings of an extended family as one household (appendix p 3). We used the log-rank test to assess the equality of the survival functions [S(t)], fitted Cox proportional hazards model adjusting for place of residence, highest level of education, household wealth, and maternal age at conception of the index pregnancy. Survey-specific adjusted hazard ratios (ie, the ratio of the hazard rate corresponding to the specific group of inter-pregnancy interval compared with the reference group [inter-pregnancy interval of 18–23 months following livebirth]) from the Cox models were depicted on forest plots and the overall HRs and region-specific HRs were estimated using meta-analysis with random effects models. We also stratified the analysis by other preceded pregnancy outcomes (stillbirth and abortion). We examined the proportional-hazards assumption of the Cox models and conducted a sensitivity analysis by excluding surveys in which the assumption of proportionality did not hold and reran the analysis for inter-pregnancy interval preceded by livebirth. All analysis were conducted in Stata (version 17.0).

### Role of the funding source

No funding was received for this project.

## Results

**Figure 1:**
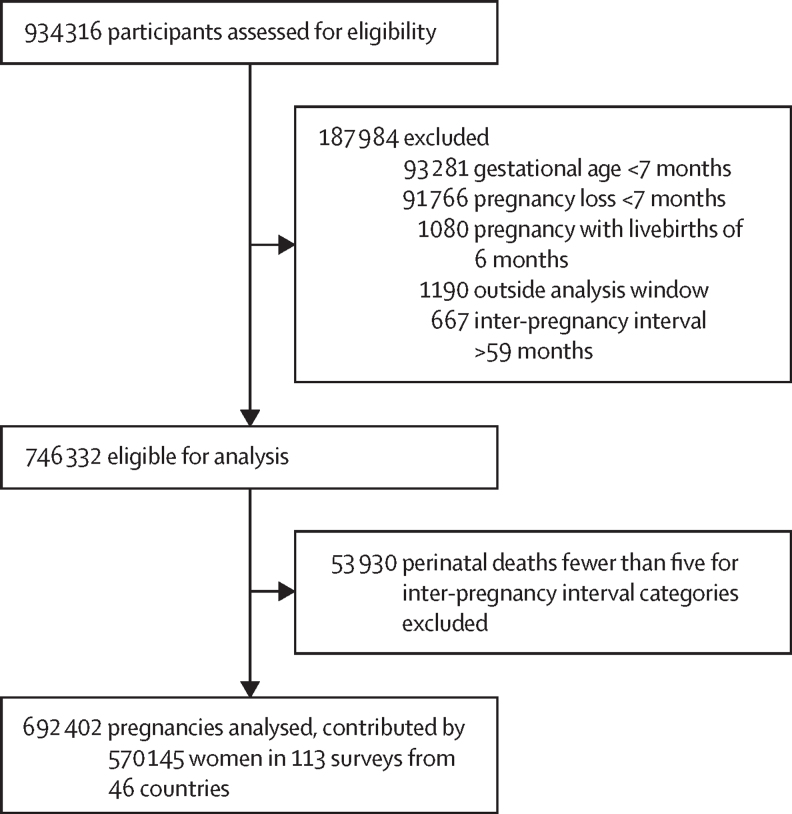
Study profile

We initially retained 145 publicly available surveys with the fieldwork starting in the year 2000 until 2022 in 59 countries. We extracted pregnancies that were conceived in the calendar with inter-pregnancy interval calculated, then excluded current pregnancies and pregnancies with a gestational age of less than 7 months, pregnancies with livebirths of 6 months (ie, extreme preterm), and we further excluded pregnancies conceived 66 months before the survey date (ie, outside the analysis window) and pregnancies with inter-pregnancy interval of more than 59 months, resulting in 746 332 pregnancies eligible for analysis (figure 1).

We also excluded 32 surveys that had an insufficient number of perinatal deaths in each of the inter-pregnancy interval category defined earlier using the minimum of five perinatal deaths as a cutoff (appendix p 2). The final analysis sample was 692 402 pregnancies contributed by 570 145 women (with a mean age of 28·4 years and SD 5·96) in 113 surveys from 46 countries—67 surveys from sub-Saharan Africa, 12 surveys from north Africa, west Asia, and Europe, 23 surveys from central, south, and southeast Asia, and 11 surveys from Latin America and the Caribbean. The survey specific distribution of the analysis sample is provided in the appendix (pp 3–11). 83% (94 of 113)of the surveys had sampled all women of reproductive age in the selected household and 17% (19 of 113) sampled ever-married women. For the sub-analyses by previous pregnancy outcomes of stillbirth and abortion, only surveys with a minimum of five perinatal deaths in each inter-pregnancy interval, conditional on the previous relevant outcome, were analysed.

The survey-specific inter-pregnancy interval medians ranged from 12 months (Jordan 2002 and 2009 surveys) to 25 months (Guinea 2005 survey), and the overall median of the median values was 17 months. 41·6% (47 of 113) of the surveys have a median inter-pregnancy interval below 18 months (appendix p 12–14). Inter-pregnancy interval by background characteristics are summarised in table 1. Women in urban settings reported slightly higher median inter-pregnancy interval than women in rural settings. The median inter-pregnancy interval is the longest in sub-Saharan Africa (19 months), and the lowest in north Africa, west Asia, and Europe (14 months). There was no observed difference in inter-pregnancy interval by level of education nor by household wealth. The median inter-pregnancy interval increased with age, and women experiencing a pregnancy loss tended to have 10 months shorter median inter-pregnancy interval than women who had a livebirth.

**Table 1:** Inter-pregnancy intervals in months by baseline characteristics

		**Median (IQR)**
Overall		17 (11–26)
Residence		
	Rural	17 (10–26)
	Urban	18 (11–26)
Region		
	Sub-Saharan Africa	19 (13–27)
	North Africa, western Asia, and Europe	14 (7–23)
	Central, south, and southeast Asia	16 (9–24)
	Latin America and the Caribbean	17 (10–25)
Highest Level of education		
	No education	18 (12–26)
	Primary	18 (11–26)
	Secondary or higher	16 (9–25)
Household wealth tertiles		
	Poor	17 (11–25)
	Middle	17 (11–26)
	Rich	17 (10–26)
Age at conception of the index pregnancy, years		
	<25	16 (9–23)
	25–29	19 (12–27)
	≥30	20 (13–28)
Previous pregnancy outcome		
	Non-livebirth	8 (3–15)
	Livebirth	18 (12–26)

Summarised over surveys and characteristics.

The survey-specific perinatal mortality rates ranged from 10·4 (95% CI 7·9–13·6) per 1000 births (Jordan 2017–18 survey) to 67·6 (61·9–73·8) per 1000 births (Pakistan 2012–13 survey; appendix pp 15–17). Table 2 shows the perinatal mortality rates by background characteristics. The overall median rate of 35·3 (30·7–41·5) per 1000 births and the median decreases with an increase in inter-pregnancy interval from 55·7 months per 1000 births for an interval shorter than 6 months to almost half (28·3 months) for the interval of 18–23 months, and a median of 32·0 months for the 24–59 months interval with an IQR decreasing by half, indicating appreciable variation within the shorter interval. The rates were higher among women from rural areas compared with women from urban areas. Women in Latin America, and north Africa, west Asia, and Europe reported lower rates of perinatal mortality compared with women in sub-Saharan Africa and central, south and southeast Asia. Perinatal mortality rates decreased with increased level of education and household wealth and increased for older women. The median perinatal mortality rates following non-livebirth was slightly higher than perinatal mortality rates following livebirth.

**Table 2:** Kaplan-Meier perinatal mortality rates by baseline characteristics

		**Median (IQR)**	**Log rank test**
Overall		35·3 (30·7–41·5)	..
Interpregnancy interval, months		..	<0·0001
	<6	55·7 (46·6–74·3)	..
	6–11	44·7 (36·0–59·8)	..
	12–17	34·5 (29·7–50·7)	..
	18–23	28·3 (26·1–34·7)	..
	24–59	32·0 (25·6–34·2)	..
Residence		..	<0·0001
	Rural	38·6 (32·7–43·4)	..
	Urban	30·7 (25·1–36·9)	..
Region		..	<0·0001
	Sub-Saharan Africa	38·4 (32·4–42·0)	..
	North Africa, western Asia, and Europe	20·2 (15·8–23·2)	..
	Central, south, and southeast Asia	35·3 (30·7–46·4)	..
	Latin America and the Caribbean	17·7 (17·6–21·4)	..
Highest Level of education		..	<0·0001
	No education	41·9 (36·4–44·8)	..
	Primary	38·0 (33·3–42·8)	..
	Secondary or higher	26·2 (25·1–32·4)	..
Household wealth tertiles		..	<0·0001
	Poor	40·7 (33·6–43·5)	..
	Middle	35·0 (29·4–39·9)	..
	Rich	30·6 (22·5–37·5)	..
Age at conception of index pregnancy (in years)		..	<0·0001
	<25	33·2 (29·5–40·9)	..
	25–29	34·0 (28·5–37·8)	..
	≥30	44·7 (39·5–49·5)	..
Previous pregnancy outcome		..	<0·0001
	Non-livebirth	38·0 (28·4–51·8)	..
	Livebirth	35·6 (31·6–40·7)	..

Data are rates per 1000 births, unless stated otherwise. Perinatal mortality rates and Kaplan-Meier estimates were stratified by survey. These perinatal mortality rates are based on a subset of women who had at least one index pregnancy within the calendar period of 7–66 months. Age at conception of the index pregnancy**.**

**Figure 2:**
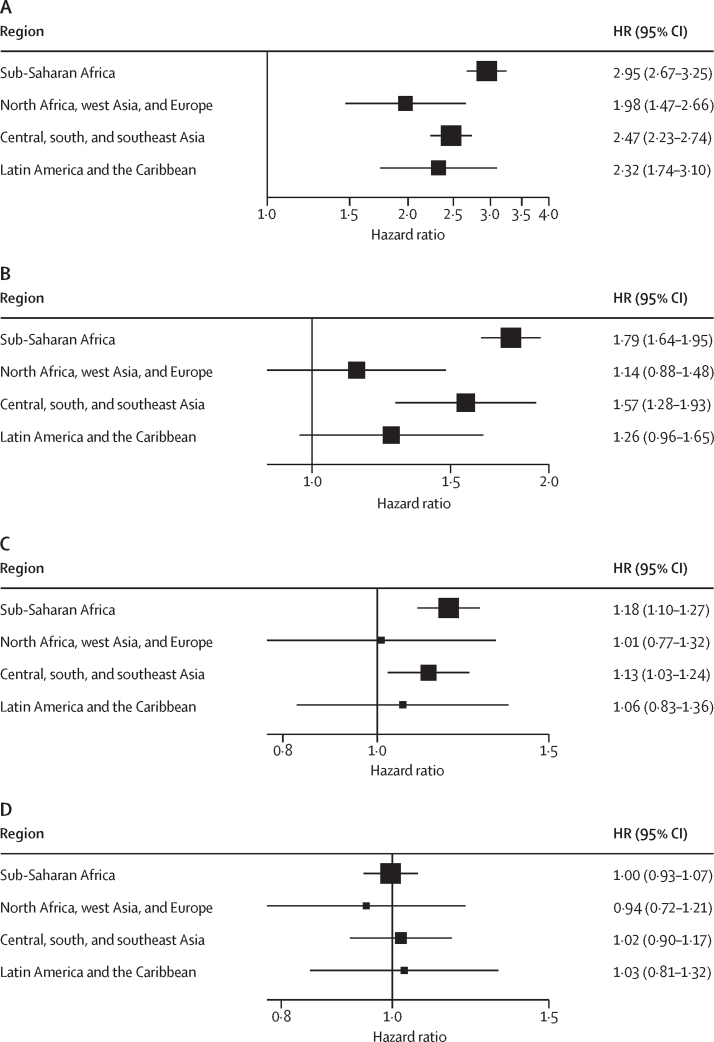
Hazard ratios of perinatal mortality by inter-pregnancy interval (A) Less than 6 months versus 18–23 months), (B) 6–11 months versus 18–23 months, (C) 12–17 months versus 18–23 months, and (D) 24–59 months versus 18–23 months

Survey-specific Kaplan-Meier survival probabilities by inter-pregnancy interval with log-rank test and level of significance are shown in the appendix (pp 18–130). Survey-specific HRs are presented in forest plots (appendix pp 131–134). The overall HR of perinatal death was 2·72 (95% CI 2·52–2·93) times higher following an inter-pregnancy interval of less than 6 months compared with the recommended optimal interval of 18–23 months following livebirth (table 3), and the HR of perinatal death decreased as the inter-pregnancy interval increased. Of 67 surveys in sub-Saharan Africa, 50 surveys had a significant HR and an overall HR of 2·95 (95% CI 2·67–3·25), followed by central, south, and southeast Asia with one of 23 surveys having significant HRs and an overall HR of 2·47 (2·23–2·74). In Latin America and the Caribbean, five of 11 surveys had significant HRs with an overall HR of 2·32 (95% CI 1·74–3·10), and for north Africa, west Asia, and Europe, two of 12 surveys had significant HRs and an overall HR of 1·98 (1·47–2·66; figure 2).

**Table 3:** Pooled hazard ratios of perinatal mortality by inter-pregnancy intervals and preceding pregnancy outcomes

		**Number of surveys**	**Pooled HR (95% CI)**
Livebirth		113	..
	<6	..	2·72 (2·52–2·93)
	6–11	..	1·64 (1·52–1·77)
	12–17	..	1·15 (1·09–1·22)
	18–23	..	1 (ref)
	24–59	..	1·00 (0·95–1·06)
Stillbirth			
	<3	3	0·90 (0·24–3·31)
	≥3	..	1 (ref)
	<6	13	1·17 (0·91–1·50)
	≥6	..	1 (ref)
	<12	12	0·98 (0·73–1·32)
	≥12	..	1 (ref)
Abortion*			
	<3	25	1·06 (0·88–1·29)
	≥3	..	1 (ref)
	<6	51	1·07 (0·96–1·19)
	≥6	..	1 (ref)
	<12	37	0·89 (0·78–1·02)
	≥12	..	1 (ref)

Inter-pregnancy intervals are defined in months. Hazard ratios (HRs) are adjusted for place of residence, woman's highest level of education, household wealth, and maternal age at conception of the index pregnancy.

* Abortion is defined as any fetal loss of less than 7 months of gestation (spontaneous or induced).

The overall HR of 6–11 months inter-pregnancy interval compared with 18–23 months was 1·64 (95% CI 1·52–1·77) and the region-specific overall HRs were significant in sub-Saharan Africa and central, south, and southeast Asia. The overall HR for the interval 12–17 months was 1·15 (1·09–1·22) compared with 18–23 months, and the region specific was also significant in the same regions as for the HRs for 6–11 months. The overall HR for the 24–59 months inter-pregnancy interval was very similar to the 18–23 months and none of the four regions showed significant overall HR (figure 2, table 3). Survey-specific assumption of proportionality test and overall HRs by inter-pregnancy interval based on the 103 surveys that did not violate the assumption of proportionality surveys are shown in the appendix (pp 134–138).

We further examined in pairwise comparisons: the hazard of inter-pregnancy interval of less than 3 months versus an inter-pregnancy interval of 3 months or more; an inter-pregnancy interval of less than 6 months versus an inter-pregnancy interval of 6 months or more; and an inter-pregnancy interval of less than 12 months versus an inter-pregnancy interval of 12 months or more following stillbirth or abortion (table 3; appendix pp 139–144). The results showed that a very short inter-pregnancy interval that started from non-livebirth (abortion or stillbirth) did not pose a greater risk of perinatal death in subsequent birth.

We also obtained the 12-month contraceptive failure rate from the Demographic and Health Survey StatCompiler^16^ for the most recent surveys that included contraceptive calendar and examined the correlation with a median inter-pregnancy interval (appendix p 145). A strong negative correlation between the 12-month contraceptive failure and inter-pregnancy interval median (–0·6635) was found (ie, the lower the failure rate, the longer the inter-pregnancy interval).

## Discussion

To our knowledge, this is the first analysis that explored the association between different inter-pregnancy intervals and perinatal mortality using the Kaplan-Meier method and Cox hazard model stratified by different outcome of the preceding pregnancy. Major findings confirmed that hazard of perinatal death is almost tripled for a shorter inter-pregnancy interval of less than 6 months following a livebirth compared with an inter-pregnancy interval of 18–23 months. Conversely, an inter-pregnancy interval shorter than 3 months, 6 months, or 12 months after a pregnancy loss is unlikely to lead to adverse perinatal mortality outcome of subsequent pregnancy.

Although a report from the Stillbirth Collaborative Research Network (a multisite case-control study conducted from 2006 to 2008) restricted to singleton pregnancies among multiparous or multigravida women (985 controls and 291 cases) revealed that 21·2% of the association of previous pregnancy loss (stillbirth, ectopic pregnancy, molar pregnancy, or spontaneous abortion) could be attributable to a short inter-pregnancy interval,^17^ more recent reports show how delaying a pregnancy following a miscarriage or a stillbirth could have no beneficial effects.^6^, ^18^ As highlighted by Kangatharan and colleagues,^6^ an inter-pregnancy interval of less than 6 months is not associated with increased risks of adverse outcomes in the pregnancy following non-livebirths compared with delaying pregnancy for at least 6 months.^6^

The same pattern was underlined in an international cohort study: conception within 12 months of a stillbirth was common and was not associated with increased risk of adverse outcomes in the subsequent pregnancy.^19^ As put forward by Klebanoff,^20^ there are no recommendations for the optimal interval after a stillbirth; counselling should probably focus more strongly on other modifiable risk factors, such as smoking, use of alcohol, and obesity while advising to try for another pregnancy as soon as the woman feels ready. Conversely, findings around the optimal spacing after a livebirth is consistent with WHO recommendations.^5^ A meta-analysis in high-resource settings showed some evidence of association between inter-pregnancy intervals shorter than 6 months since last livebirth and increased risks for preterm birth, small-for-gestational age, and infant death; however, results were inconsistent.^21^ A community-based prospective cohort study of women with an inter-pregnancy interval of less than 18 months following a livebirth showed increased risk of stillbirth.^22^

Several hypotheses, including nutritional depletion and anaemia, have been proposed as explanations for adverse birth outcomes of short inter-pregnancy interval. According to the nutritional depletion hypothesis, mothers are not given sufficient time to recover from nutritional deficiencies after the pregnancy and subsequent breastfeeding.^23^, ^24^ An alternative hypothesis is that a short inter-pregnancy interval might leave insufficient recovery time from inflammatory processes from a previous pregnancy that extends into the next pregnancy.^25^

Several studies have examined the association between inter-pregnancy interval and subsequent adverse perinatal and maternal outcomes, such as small-for-gestational-age birth, preterm birth, low birthweight, child mortality, maternal mortality and morbidity and, to a lesser extent, stillbirth, and early neonatal death.^2^, ^3^, ^10^ Our finding of a strong negative correlation between a 12-month contraceptive failure rate and inter-pregnancy interval median is quite unique in the existing literature when considering analysis of data from LMICs. Similarly, an analysis using the US 2006–10 National Survey of Family Growth showed that less effective contraceptive use was the leading predictor of having a short inter-pregnancy interval after controlling for women's sociodemographic characteristics.^26^ This finding re-emphasises the need for an integrated approach when counselling women around suitable timing for pregnancies, correct family planning approach, and addressing modifiable risk factors.

The role of confounding factors is context-related and particularly evident in the work by Molitoris and colleagues,^27^ in which mortality-reducing effects of longer birth intervals are strong in low-income countries but decline steadily toward zero in higher-income countries. Our analysis suggests a similar pattern with the highest HRs (2·95, 95% CI 2·67–3·25) being in sub-Saharan Africa whereas the lowest HR being in north Africa, west Asia, and Europe (1·98, 1·47–2·66). This finding is a crucial piece to reconcile previous research indicating a different role of birth intervals on perinatal outcomes when comparing high-income settings with low-income settings.^27^

Various methodological aspects must be considered when assessing the role of inter-pregnancy interval—for instance, assessing how pregnancy terminations might have influenced inter-pregnancy interval classifications. Further, most studies, including this study, on inter-pregnancy interval and adverse birth outcomes are traditional retrospective cohort studies that do not adjust for certain unmeasured confounders.

To our knowledge, this is the first study using a statistically robust time-to-event approach to assess the effect of inter-pregnancy interval on perinatal mortality. This study, however, has some limitations. We were able to include only those sources of confounding for which data were available. One notable variable is pregnancy intention, which is collected by the Demographic and Health Survey for livebirths but not for stillbirths. Residual confounding by these variables might have led to overestimation or underestimation of the effect of inter-pregnancy interval on subsequent risk of stillbirth.

The accuracy of the Demographic and Health Survey reproductive calendar for the calculation of inter-pregnancy interval has not been formally assessed. However, some of the studies that have examined the quality of Demographic and Health Survey calendar data concluded that the contraceptive histories are acceptably detailed and better than direct survey questions.^28^, ^29^ Consistencies in reporting of pregnancy terminations were found in 50 (31%) of 162 calendars in the surveys from the Demographic and Health Survey Program from 62 countries.^30^More notably, under-reporting of terminated pregnancies was greater in earlier years. Another study based on 157 Demographic and Health Survey calendar data from 53 countries showed that age heaping at day 7 occurred in most surveys.^12^ Similarly, under-reporting of stillbirths was a much more common issue with only 23 of 157 surveys (15%) considered to have plausible ratios when confronted with deaths on days 0–1. Although age heaping does not significantly affect the estimates of perinatal mortality because of low number of deaths after the first few days of life, the omission of stillbirths needs to be considered. If the true number of stillbirths to the mothers in our sample was greater than those recorded in the data, our estimates of risk of stillbirth could be low. In this regard, the magnitude of the overall HR for inter-pregnancy interval less than 6 months among those with a previous stillbirth was 1·17, close to statistical significance (95% CI 0·91–1·50). Further, findings related to periods shorter than 3 months relied on only three surveys.

The reproductive calendar does not contain data about spontaneous miscarriages that occurred without the mother knowing; however, early miscarriage is notoriously difficult to capture in any perinatal study. As far as time-variant covariates are concerned, including socioeconomic status, these were measured at the time of the survey and could have potentially changed throughout the 5-year period we considered. We worked with cross-sectional data, which limits the identification of a causal relationship between inter-pregnancy interval and adverse perinatal outcomes. Also, the data do not provide context-specific information that is essential in understanding the regional and country comparisons. As common in all surveys eliciting information from a respondent, birth histories and survival information were only collected from surviving mothers.

Our study reaffirms the evidence underlying the WHO recommendation on optimal interval between the last livebirth and the next pregnancy of at least 24 months and avoiding pregnancy before 18 months. However, the association between short inter-pregnancy interval and risk of perinatal mortality is greater in contexts with fewer resources than in those with more resources.

We found no beneficial effect of the WHO recommended interval of 6 months from a pregnancy loss to the next pregnancy or longer inter-pregnancy interval on the subsequent pregnancy risk of perinatal mortality. In LMICs that account for most of the perinatal deaths, most of these deaths can be averted by optimal birth spacing through contraceptive use.

## Data sharing

All data are available at https://www.dhsprogram.com/data/availabledatasets.com. The Demographic and Health Survey Program is authorised to distribute, at no cost, unrestricted survey data files for legitimate academic research. Registration is required to be able to download the data. Researchers are required to provide contact information, the research title, and a description of the proposed analysis. Approval to access datasets is usually granted and communicated via email. The data are third party data that are not owned or collected by the authors and the authors do not have any special access privileges. The created analysis files are available upon request to the corresponding author.

## Declaration of interests

MMA and SB are staff members of WHO, and IHS is affiliated with Harvard T H Chan School of Public Health. The authors alone are responsible for the views expressed in this publication, and they do not necessarily represent the decisions, policy, or views of WHO or Harvard University. The country names used do not imply the expression of any opinion whatsoever on the part of WHO or Harvard University concerning the legal status of any country, territory, city or area, or of its authorities.
