# Supplementary material for: The risk of perinatal mortality following short inter-pregnancy intervals—insights from 692 402 pregnancies in 113 Demographic and Health Surveys from 46 countries: a population-based analysis
Source: Lancet Glob Health. 2023 Sep 19;11(10):e1544–52. doi: 10.1016/S2214-109X(23)00359-5 (PMC10522774; doi:10.1016/S2214-109X(23)00359-5)

THE LANCET
Global Health

Supplementary appendix

This appendix formed part of the original submission and has been peer reviewed. We post it as supplied by the authors.

Supplement to: Ali MM, Bellizzi S, Shah IH. The risk of perinatal mortality following short inter-pregnancy intervals—insights from 692 402 pregnancies in 113 Demographic and Health Surveys from 46 countries: a population-based analysis. *Lancet Glob Health* 2023; **11:** e1544–52.

Table of Contents

List of surveys excluded due to insufficient number (<5) of perinatal deaths in each inter-pregnancy Interval (IPI) 2

E-Table 1a: Number of clusters, households, women, and pregnancies in the analysis sample, by survey 3

E-Table 1b: Number of pregnancies per woman during the analysis period, by survey 6

E-Table 2: Pregnancy outcome, by survey 9

E-Table 3: Percentiles of inter-pregnancy intervals (months), by survey 12

E-Table 4: Perinatal mortality rates (PNMRs) with 95% confidence interval (CI), per 1000 births 15

E-Figure 1: Survey-specific Kaplan-Meier survival probabilities, by IPI group (in months) 18

E-Figure 2: Survey-specific hazard ratios (HRs) of perinatal mortality, by IPI (months) following livebirth 131

E-Table 6: Tests of proportional-hazards assumption 135

E-Table 7: The pooled hazard ratios (HRs) of perinatal mortality by IPI preceded by livebirth for surveys that met the proportionality assumption 138

E-Figure 3: Survey-specific hazard ratios of perinatal mortality, by IPI (months) following Stillbirth 139

E-Figure 4: Survey-specific hazard ratios of perinatal mortality, by IPI (months) following abortion 142

E-Figure 5: Correlation of median IPI with contraceptive failure rate, at survey level, for the most recent DHS surveys 145

List of surveys excluded due to insufficient number (<5) of perinatal deaths in each inter-pregnancy Interval (IPI)

Region	Country	Year of the survey
sub-Saharan Africa		
	Comoros	2012
	Lesotho	2014
	Namibia	2013
	South Africa	2016
	Eswatini	2006/7
	Zimbabwe	2005/6
North Africa, Western Asia and Europe		
	Albania	2008/9
	Albania	2017/18
	Armenia	2000
	Armenia	2005
	Armenia	2010
	Armenia	2015/16
	Azerbaijan	2006
	Jordan	2007
	Moldova	2005
	Türkiye	2018/19
	Ukraine	2007
Central Asia		
	Kyrgyz Republic	2012
	Tajikistan	2012
	Tajikistan	2017
South & Southeast Asia		
	Cambodia	2014
	Cambodia	2021/22
	Maldives	2009
	Vietnam	2002
Latin America & Caribbean		
	Colombia	2000
	Colombia	2009/10
	Colombia	2015/16
	Guyana	2009
	Peru	2004/6
	Peru	2010
	Peru	2011
	Peru	2012

E-Table 1a: Number of clusters, households, women, and pregnancies in the analysis sample, by survey

Region	Sample	No. of Clusters	No. of households	HHs per cluster	No. of women	Women per HH	No. of pregnancies	Pregnancies per woman
**sub-Saharan Africa**								
Angola (2015/16)	AW	625	5,615	8.98	5,738	1.02	7,821	1.36
Benin (2006)	AW	745	5,858	7.86	6,392	1.09	7,659	1.20
Benin (2011/12)	AW	746	4,951	6.64	5,246	1.06	6,372	1.21
Benin (2017/18)	AW	554	4,965	8.96	5,515	1.11	6,854	1.24
Burkina Faso (2003)	AW	400	3,566	8.92	4,272	1.20	4,902	1.15
Burkina Faso (2010)	AW	573	5,260	9.18	5,974	1.14	6,938	1.16
Burundi (2010/11)	AW	376	3,300	8.78	3,316	1.00	4,319	1.30
Ethiopia (2005)	AW	508	3,786	7.45	3,807	1.01	4,792	1.26
Ethiopia (2011)	AW	563	4,196	7.45	4,234	1.01	5,288	1.25
Ethiopia (2016)	AW	596	3,756	6.30	3,788	1.01	4,914	1.30
Gambia (2013)	AW	279	2,231	8.00	2,839	1.27	3,230	1.14
Gambia (2019/20)	AW	277	2,611	9.43	3,540	1.36	4,485	1.27
Ghana (2003)	AW	375	1,320	3.52	1,365	1.03	1,598	1.17
Ghana (2008)	AW	363	1,031	2.84	1,058	1.03	1,240	1.17
Ghana (2014)	AW	417	2,040	4.89	2,094	1.03	2,461	1.18
Guinea (2005)	AW	292	2,015	6.90	2,261	1.12	2,528	1.12
Guinea (2018)	AW	399	2,651	6.64	2,939	1.11	3,428	1.17
Kenya (2003)	AW	389	1,973	5.07	2,000	1.01	2,530	1.27
Kenya (2008/9)	AW	384	2,194	5.71	2,228	1.02	2,932	1.32
Kenya (2014)	AW	1,275	3,264	2.56	3,291	1.01	4,007	1.22
Kenya (2022)	AW	1,258	2,961	2.35	2,989	1.01	3,656	1.22
Lesotho (2009/10)	AW	358	1,107	3.09	1,133	1.02	1,293	1.14
Liberia (2013)	AW	318	2,352	7.40	2,488	1.06	2,893	1.16
Liberia (2019/20)	AW	322	1,875	5.82	1,943	1.04	2,326	1.20
Madagascar (2003/4)	AW	297	2,042	6.88	2,075	1.02	2,715	1.31
Madagascar (2008/9)	AW	593	4,708	7.94	4,788	1.02	6,206	1.30
Madagascar (2021)	AW	633	3,614	5.71	3,650	1.01	4,377	1.20
Malawi (2000)	AW	558	4,430	7.94	4,513	1.02	5,384	1.19
Malawi (2004/5)	AW	519	4,296	8.28	4,354	1.01	5,275	1.21
Malawi (2010)	AW	846	7,274	8.60	7,367	1.01	8,508	1.15
Malawi (2015/16)	AW	843	5,602	6.65	5,660	1.01	6,286	1.11
Mali (2001)	AW	402	1,598	3.98	4,951	3.10	5,856	1.18
Mali (2006)	AW	407	5,223	12.83	5,724	1.10	7,138	1.25
Mali (2012/13)	AW	413	3,863	9.35	4,160	1.08	5,367	1.29
Mali (2018)	AW	345	3,649	10.58	3,958	1.08	5,081	1.28
Mozambique (2003/4)	AW	597	3,799	6.36	4,026	1.06	4,938	1.23
Mozambique (2011)	AW	604	3,956	6.55	4,082	1.03	4,867	1.19
Namibia (2006/7)	AW	470	1,504	3.20	1,585	1.05	1,839	1.16
Niger (2006)	AW	341	3,059	8.97	3,422	1.12	3,984	1.16
Niger (2012)	AW	474	4,549	9.60	5,036	1.11	6,140	1.22
Nigeria (2008)	AW	884	10,442	11.81	11,480	1.10	14,800	1.29
Nigeria (2013)	AW	893	10,956	12.27	12,061	1.10	14,879	1.23

Nigeria (2018)	AW	1,384	12,647	9.14	13,961	1.10	18,197	1.30
Rwanda (2000)	AW	442	3,169	7.17	3,188	1.01	4,206	1.32
Rwanda (2005)	AW	462	3,367	7.29	3,380	1.00	4,349	1.29
Rwanda (2010/11)	AW	491	3,526	7.18	3,542	1.00	4,344	1.23
Rwanda (2014/15)	AW	488	2,781	5.70	2,797	1.01	3,335	1.19
Rwanda (2019/20)	AW	496	2,922	5.89	2,927	1.00	3,461	1.18
Senegal (2005)	AW	372	3,129	8.41	4,004	1.28	4,783	1.19
Senegal (2010/11)	AW	390	3,795	9.73	5,021	1.32	6,459	1.29
Senegal (2015)	AW	213	1,930	9.06	2,584	1.34	3,045	1.18
Senegal (2016)	AW	213	1,872	8.79	2,448	1.31	2,872	1.17
Senegal (2018)	AW	212	1,897	8.95	2,510	1.32	2,939	1.17
Senegal (2019)	AW	211	1,747	8.28	2,238	1.28	2,602	1.16
Sierra Leone (2008)	AW	338	1,789	5.29	1,896	1.06	2,217	1.17
Sierra Leone (2013)	AW	434	4,001	9.22	4,275	1.07	4,976	1.16
Tanzania (2004/5)	AW	469	3,292	7.02	3,474	1.06	4,524	1.30
Tanzania (2010)	AW	461	2,578	5.59	2,715	1.05	3,242	1.19
Tanzania (2015/16)	AW	589	3,558	6.04	3,758	1.06	4,782	1.27
Uganda (2000/1)	AW	294	2,870	9.76	2,978	1.04	4,222	1.42
Uganda (2006)	AW	365	3,269	8.96	3,369	1.03	4,456	1.32
Uganda (2011)	AW	403	3,140	7.79	3,222	1.03	4,279	1.33
Zambia (2007)	AW	317	2,322	7.32	2,384	1.03	2,885	1.21
Zambia (2013/14)	AW	720	5,066	7.04	5,209	1.03	6,364	1.22
Zambia (2018/19)	AW	539	3,304	6.13	3,382	1.02	3,898	1.15
Zimbabwe (2010/11)	AW	389	1,683	4.33	1,718	1.02	1,937	1.13
Zimbabwe (2015)	AW	388	1,787	4.61	1,820	1.02	2,029	1.11
**North Africa, Western Asia & Europe**								
Egypt (2000)	EMW	489	3,478	7.11	3,670	1.06	4,388	1.20
Egypt (2003)	EMW	749	2,142	2.86	2,240	1.05	2,615	1.17
Egypt (2005)	EMW	671	4,225	6.30	4,478	1.06	5,233	1.17
Egypt (2008)	EMW	1,076	3,139	2.92	3,253	1.04	3,673	1.13
Egypt (2014)	EMW	830	4,954	5.97	5,066	1.02	5,832	1.15
Jordan (2002)	EMW	490	2,426	4.95	2,461	1.01	3,327	1.35
Jordan (2009)	EMW	909	3,935	4.33	3,965	1.01	5,389	1.36
Jordan (2012)	EMW	798	4,223	5.29	4,269	1.01	5,651	1.32
Jordan (2017/18)	EMW	933	4,094	4.39	4,135	1.01	5,313	1.28
Morocco (2003/4)	AW	461	2,048	4.44	2,128	1.04	2,546	1.20
Türkiye (2003/4)	EMW	505	1,576	3.12	1,627	1.03	2,135	1.31
Yemen (2013)	AW	774	5,960	7.70	6,335	1.06	8,485	1.34
**Central, South & Southeast Asia**								
Bangladesh (2004)	EMW	359	2,251	6.27	2,300	1.02	2,727	1.19
Bangladesh (2007)	EMW	349	1,609	4.61	1,648	1.02	1,853	1.12
Bangladesh (2011)	EMW	559	2,177	3.89	2,235	1.03	2,480	1.11
Bangladesh (2014)	EMW	526	1,718	3.27	1,742	1.01	1,899	1.09
Bangladesh (2017/18)	EMW	617	2,163	3.51	2,203	1.02	2,427	1.10
Cambodia (2010/11)	AW	592	2,640	4.46	2,662	1.01	3,091	1.16
India (2005/6)	AW	3,594	17,971	5.00	18,541	1.03	23,029	1.24
India (2015/16)	AW	24,500	77,540	3.16	79,558	1.03	93,613	1.18
India (2019/21)	AW	24,734	71,359	2.89	73,051	1.02	86,095	1.18
Indonesia (2002/3)	EMW	1,230	4,217	3.43	4,253	1.01	4,760	1.12
Indonesia (2007)	EMW	1,463	4,467	3.05	4,501	1.01	5,092	1.13

Indonesia (2012)	AW	1,488	3,838	2.58	3,876	1.01	4,305	1.11
Indonesia (2017)	AW	1,623	4,014	2.47	4,064	1.01	4,488	1.10
Myanmar (2015/16)	AW	367	1,368	3.73	1,380	1.01	1,685	1.22
Nepal (2006)	AW	256	2,170	8.48	2,252	1.04	2,762	1.23
Nepal (2011)	AW	279	1,806	6.47	1,851	1.02	2,225	1.20
Nepal (2016/17)	AW	353	1,418	4.02	1,464	1.03	1,679	1.15
Nepal (2021/22)	AW	425	1,620	3.81	1,659	1.02	1,915	1.15
Pakistan (2012/13)	EMW	496	4,533	9.14	4,968	1.10	7,056	1.42
Pakistan (2017/18)	EMW	456	3,866	8.48	4,290	1.11	5,849	1.36
Philippines (2003)	AW	737	2,388	3.24	2,410	1.01	3,100	1.29
Philippines (2022)	AW	885	1,917	2.17	1,931	1.01	2,211	1.15
Timor-Leste (2009/10)	AW	454	3,895	8.58	3,963	1.02	5,396	1.36
**Latin America & Caribbean**								
Bolivia (2003/4)	AW	931	3,729	4.01	3,763	1.01	4,856	1.29
Bolivia (2008)	AW	860	2,503	2.91	2,525	1.01	2,988	1.18
Colombia (2005)	AW	2,330	4,496	1.93	4,576	1.02	5,526	1.21
Dominican Republic (2002)	AW	1,187	3,871	3.26	3,914	1.01	4,933	1.26
Guatemala (2014/15)	AW	822	4,033	4.91	4,134	1.03	5,052	1.22
Honduras (2005/6)	AW	977	3,832	3.92	3,905	1.02	4,933	1.26
Honduras (2011/12)	AW	1,020	3,260	3.20	3,320	1.02	3,876	1.17
Nicaragua (2001)	AW	561	2,199	3.92	2,274	1.03	2,856	1.26
Peru (2000)	AW	1,187	4,148	3.49	4,201	1.01	5,002	1.19
Peru (2007/8)	AW	796	2,297	2.89	2,327	1.01	2,617	1.12
Peru (2009)	AW	935	2,600	2.78	2,635	1.01	2,930	1.11
**All surveys**		120,224	540,996	4.50	570,145	1.05	692,402	1.21

AW=All woman, EMW=Ever-married woman, HH=Household.
The analysis sample includes all index pregnancies conceived between 7-66 months before the survey month (the analysis period).

E-Table 1b: Number of pregnancies per woman during the analysis period, by survey

**Region**	No. of pregnancies*	Number of women with:					
		1	2	3	4	5	6+
**sub-Saharan Africa**							
Angola (2015/16)	7,942	3,756	1,767	209	5	1	
Benin (2006)	7,871	5,009	1,295	80	8		
Benin (2011/12)	6,538	4,086	1,042	105	12	1	
Benin (2017/18)	7,031	4,124	1,275	107	9		
Burkina Faso (2003)	4,980	3,595	647	29	1		
Burkina Faso (2010)	7,078	4,926	996	48	4		
Burundi (2010/11)	4,359	2,340	909	67			
Ethiopia (2005)	4,833	2,882	837	77	9	2	
Ethiopia (2011)	5,372	3,219	905	97	13		
Ethiopia (2016)	4,983	2,761	877	133	16	1	
Gambia (2013)	3,270	2,425	398	15	1		
Gambia (2019/20)	4,566	2,581	899	55	3	2	
Ghana (2003)	1,630	1,121	223	21			
Ghana (2008)	1,268	863	181	13	1		
Ghana (2014)	2,512	1,706	360	26	2		
Guinea (2005)	2,588	1,949	299	11	2		
Guinea (2018)	3,496	2,417	490	29	3		
Kenya (2003)	2,569	1,497	438	64	1		
Kenya (2008/9)	2,971	1,567	587	67	6	1	
Kenya (2014)	4,054	2,579	665	43	4		
Kenya (2022)	3,713	2,352	554	80	2	1	
Lesotho (2009/10)	1,315	963	160	9		1	
Liberia (2013)	2,950	2,055	408	21	4		
Liberia (2019/20)	2,375	1,550	357	33	3		
Madagascar (2003/4)	2,729	1,499	502	70	4		
Madagascar (2008/9)	6,261	3,467	1,182	126	13		
Madagascar (2021)	4,415	2,955	631	59	4	1	
Malawi (2000)	5,495	3,605	841	61	5	1	
Malawi (2004/5)	5,359	3,423	861	66	4		
Malawi (2010)	8,680	6,131	1,161	73	2		
Malawi (2015/16)	6,405	4,951	677	28	4		
Mali (2001)	5,935	4,036	847	67	1		
Mali (2006)	7,252	4,311	1,311	94	3	5	
Mali (2012/13)	5,458	3,005	1,023	122	9	1	
Mali (2018)	5,164	2,861	997	92	7	1	
Mozambique (2003/4)	5,049	3,073	890	58	3	2	
Mozambique (2011)	4,960	3,273	744	62	2	1	
Namibia (2006/7)	1,863	1,333	227	24	1		
Niger (2006)	4,061	2,826	554	41	1		
Niger (2012)	6,244	3,909	1,051	71	5		
Nigeria (2008)	15,031	8,235	2,968	253	23		1
Nigeria (2013)	15,113	9,230	2,633	182	13	2	1
Nigeria (2018)	18,526	9,850	3,691	387	32	1	

Rwanda (2000)	4,256	2,235	852	88	12	1
Rwanda (2005)	4,396	2,432	886	57	4	1
Rwanda (2010/11)	4,403	2,755	716	68	3	
Rwanda (2014/15)	3,373	2,261	497	38	1	
Rwanda (2019/20)	3,499	2,393	497	36	1	
Senegal (2005)	4,864	3,191	768	43	2	
Senegal (2010/11)	6,574	3,606	1,283	126	6	
Senegal (2015)	3,107	2,087	474	20	3	
Senegal (2016)	2,932	1,988	437	22	1	
Senegal (2018)	2,996	2,053	429	27	1	
Senegal (2019)	2,652	1,843	377	17	1	
Sierra Leone (2008)	2,256	1,566	303	24	3	
Sierra Leone (2013)	5,057	3,527	714	34		
Tanzania (2004/5)	4,609	2,448	927	90	8	1
Tanzania (2010)	3,286	2,181	500	32	1	1
Tanzania (2015/16)	4,854	2,756	916	78	8	
Uganda (2000/1)	4,281	1,825	1,013	131	8	1
Uganda (2006)	4,521	2,317	958	89	4	1
Uganda (2011)	4,337	2,205	925	86	6	
Zambia (2007)	2,935	1,863	492	28	1	
Zambia (2013/14)	6,471	4,031	1,097	78	3	
Zambia (2018/19)	3,954	2,850	497	30	5	
Zimbabwe (2010/11)	1,967	1,491	207	18	2	
Zimbabwe (2015)	2,062	1,595	209	15	1	
**North Africa, Western Asia & Europe**						
Egypt (2000)	4,471	2,938	667	61	4	
Egypt (2003)	2,654	1,855	357	27	1	
Egypt (2005)	5,327	3,680	747	51		
Egypt (2008)	3,736	2,801	423	27	2	
Egypt (2014)	5,922	4,258	762	44	2	
Jordan (2002)	3,374	1,665	687	101	8	
Jordan (2009)	5,465	2,683	1,082	183	16	1
Jordan (2012)	5,736	2,995	1,097	162	14	1
Jordan (2017/18)	5,367	3,032	985	107	11	
Morocco (2003/4)	2,585	1,709	385	30	4	
Türkiye (2003/4)	2,152	1,190	357	73	6	1
Yemen (2013)	8,553	4,398	1,681	232	23	1
**Central, South & Southeast Asia**						
Bangladesh (2004)	2,750	1,889	374	35	2	
Bangladesh (2007)	1,868	1,444	189	14	1	
Bangladesh (2011)	2,496	1,988	234	12	1	
Bangladesh (2014)	1,911	1,585	145	12		
Bangladesh (2017/18)	2,446	1,971	222	9	1	
Cambodia (2010/11)	3,116	2,243	385	33	1	
India (2005/6)	23,172	14,372	3,730	416	23	
India (2015/16)	94,283	65,931	12,576	1,005	45	1
India (2019/21)	86,735	60,367	11,722	924	38	
Indonesia (2002/3)	4,791	3,750	472	28	2	1
Indonesia (2007)	5,121	3,918	547	35	1	
Indonesia (2012)	4,341	3,448	394	31	3	

Indonesia (2017)	4,511	3,642	397	25			
Myanmar (2015/16)	1,702	1,091	257	31	1		
Nepal (2006)	2,787	1,760	451	39	2		
Nepal (2011)	2,243	1,492	328	29	2		
Nepal (2016/17)	1,690	1,259	184	21			
Nepal (2021/22)	1,927	1,406	238	15			
Pakistan (2012/13)	7,122	3,185	1,456	283	44		
Pakistan (2017/18)	5,924	2,912	1,141	218	19		
Philippines (2003)	3,124	1,778	556	70	6		
Philippines (2022)	2,230	1,655	253	23			
Timor-Leste (2009/10)	5,449	2,606	1,235	116	5	1	
**Latin America & Caribbean**							
Bolivia (2003/4)	4,884	2,755	902	99	7		
Bolivia (2008)	3,003	2,073	426	26			
Colombia (2005)	5,576	3,681	791	103	1		
Dominican Republic (2002)	4,993	2,960	840	104	9	1	
Guatemala (2014/15)	5,089	3,245	825	62	2		
Honduras (2005/6)	4,969	2,924	903	73	5		
Honduras (2011/12)	3,909	2,775	501	44			
Nicaragua (2001)	2,880	1,736	475	58	5		
Peru (2000)	5,039	3,425	715	60	1		
Peru (2007/8)	2,634	2,040	268	18	1		
Peru (2009)	2,952	2,333	287	15			
**All surveys**	700,915	450,638	108,983	87	6	39	2

The analysis period is 7-66 months before the survey month

* Including pregnancies ending in multiple livebirths.

E-Table 2: Pregnancy outcome, by survey

Region<br>Survey	Stillbirth	Currently pregnant	Livebirth*	Single birth	Multiple birth
**sub-Saharan Africa**					
Angola (2015/16)	57	375	7,510	7,270	240
Benin (2006)	81	484	7,306	6,886	420
Benin (2011/12)	36	311	6,191	5,836	355
Benin (2017/18)	53	366	6,612	6,261	351
Burkina Faso (2003)	53	318	4,609	4,454	155
Burkina Faso (2010)	69	448	6,561	6,284	277
Burundi (2010/11)	68	259	4,032	3,952	80
Ethiopia (2005)	35	338	4,460	4,378	82
Ethiopia (2011)	67	359	4,946	4,778	168
Ethiopia (2016)	46	338	4,599	4,461	138
Gambia (2013)	37	237	2,996	2,917	79
Gambia (2019/20)	84	284	4,198	4,039	159
Ghana (2003)	15	117	1,498	1,434	64
Ghana (2008)	14	74	1,180	1,124	56
Ghana (2014)	26	147	2,339	2,238	101
Guinea (2005)	25	232	2,331	2,211	120
Guinea (2018)	51	204	3,241	3,105	136
Kenya (2003)	36	170	2,363	2,285	78
Kenya (2008/9)	28	135	2,808	2,730	78
Kenya (2014)	42	226	3,786	3,692	94
Kenya (2022)	68	238	3,407	3,294	113
Lesotho (2009/10)	15	62	1,238	1,195	43
Liberia (2013)	34	233	2,683	2,570	113
Liberia (2019/20)	29	139	2,207	2,109	98
Madagascar (2003/4)	26	159	2,544	2,516	28
Madagascar (2008/9)	70	320	5,871	5,762	109
Madagascar (2021)	58	262	4,095	4,019	76
Malawi (2000)	53	413	5,029	4,808	221
Malawi (2004/5)	63	353	4,943	4,777	166
Malawi (2010)	113	522	8,045	7,702	343
Malawi (2015/16)	73	387	5,945	5,708	237
Mali (2001)	69	472	5,394	5,237	157
Mali (2006)	87	499	6,666	6,439	227
Mali (2012/13)	34	277	5,147	4,965	182
Mali (2018)	64	280	4,820	4,654	166
Mozambique (2003/4)	72	296	4,681	4,460	221
Mozambique (2011)	43	355	4,562	4,376	186
Namibia (2006/7)	18	126	1,719	1,671	48
Niger (2006)	44	329	3,688	3,536	152
Niger (2012)	88	442	5,714	5,508	206
Nigeria (2008)	114	948	13,969	13,513	456
Nigeria (2013)	163	1,221	13,729	13,262	467
Nigeria (2018)	297	1,174	17,055	16,404	651

Rwanda (2000)	68	265	3,923	3,823	100
Rwanda (2005)	68	265	4,063	3,969	94
Rwanda (2010/11)	67	206	4,130	4,012	118
Rwanda (2014/15)	52	178	3,143	3,067	76
Rwanda (2019/20)	46	173	3,280	3,204	76
Senegal (2005)	86	307	4,471	4,310	161
Senegal (2010/11)	114	351	6,109	5,878	231
Senegal (2015)	66	188	2,853	2,729	124
Senegal (2016)	40	189	2703	2,585	118
Senegal (2018)	48	217	2731	2,619	112
Senegal (2019)	49	185	2418	2,317	101
Sierra Leone (2008)	19	163	2074	1,996	78
Sierra Leone (2013)	37	342	4678	4,516	162
Tanzania (2004/5)	85	284	4240	4,071	169
Tanzania (2010)	57	241	2988	2,900	88
Tanzania (2015/16)	81	260	4513	4,370	143
Uganda (2000/1)	68	230	3983	3,865	118
Uganda (2006)	62	280	4179	4,048	131
Uganda (2011)	71	267	3999	3,883	116
Zambia (2007)	31	198	2706	2,607	99
Zambia (2013/14)	84	340	6047	5,835	212
Zambia (2018/19)	52	266	3636	3,524	112
Zimbabwe (2010/11)	18	167	1782	1,723	59
Zimbabwe (2015)	27	119	1916	1,850	66
**North Africa, Western Asia & Europe**					
Egypt (2000)	73	227	4171	4,007	164
Egypt (2003)	31	138	2485	2,406	79
Egypt (2005)	50	276	5001	4,813	188
Egypt (2008)	29	212	3495	3,369	126
Egypt (2014)	32	357	5533	5,356	177
Jordan (2002)	34	211	3129	3,038	91
Jordan (2009)	54	331	5080	4,928	152
Jordan (2012)	38	287	5411	5,245	166
Jordan (2017/18)	15	358	4994	4,888	106
Morocco (2003/4)	40	163	2382	2,308	74
Türkiye (2003/4)	34	104	2014	1,980	34
Yemen (2013)	130	539	7884	7,748	136
**Central, South & Southeast Asia**					
Bangladesh (2004)	94	148	2508	2,462	46
Bangladesh (2007)	46	123	1699	1,669	30
Bangladesh (2011)	49	184	2263	2,231	32
Bangladesh (2014)	44	152	1715	1,691	24
Bangladesh (2017/18)	50	155	2241	2,203	38
Cambodia (2010/11)	21	201	2894	2,845	49
India (2005/6)	358	1387	21427	21,141	286
India (2015/16)	1049	5725	87509	86,162	1347
India (2019/21)	841	4963	80931	79,656	1275
Indonesia (2002/3)	46	318	4427	4,365	62
Indonesia (2007)	48	345	4728	4,670	58
Indonesia (2012)	45	327	3969	3,897	72

Indonesia (2017)	36	351	4124	4,079	45
Myanmar (2015/16)	17	89	1596	1,563	33
Nepal (2006)	68	135	2584	2,534	50
Nepal (2011)	26	113	2104	2,068	36
Nepal (2016/17)	26	102	1562	1,538	24
Nepal (2021/22)	26	107	1794	1,770	24
Pakistan (2012/13)	193	392	6537	6,406	131
Pakistan (2017/18)	137	329	5458	5,309	149
Philippines (2003)	35	191	2898	2,850	48
Philippines (2022)	22	124	2084	2,046	38
Timor-Leste (2009/10)	13	261	5175	5,069	106
**Latin America & Caribbean**					
Bolivia (2003/4)	48	233	4603	4,547	56
Bolivia (2008)	33	191	2779	2,749	30
Colombia (2005)	44	264	5268	5,169	99
Dominican Republic (2002)	54	267	4672	4,554	118
Guatemala (2014/15)	49	294	4746	4,672	74
Honduras (2005/6)	56	251	4662	4,591	71
Honduras (2011/12)	37	233	3639	3,573	66
Nicaragua (2001)	26	158	2696	2,648	48
Peru (2000)	44	293	4702	4,629	73
Peru (2007/8)	24	199	2411	2,377	34
Peru (2009)	21	198	2733	2,689	44
**All surveys**	8,300	42,616	649,999	633,029	16,970

* Including multiple livebirths.

E-Table 3: Percentiles of inter-pregnancy intervals (months), by survey

Region<br>Survey	P25	Median	P75	IQR
**sub-Saharan Africa**				
Angola (2015/16)	13	18	25	12
Benin (2006)	14	21	28	14
Benin (2011/12)	15	21	29	14
Benin (2017/18)	15	21	28	13
Burkina Faso (2003)	16	23	30	14
Burkina Faso (2010)	16	22	29	13
Burundi (2010/11)	12	18	25	13
Ethiopia (2005)	13	20	29	16
Ethiopia (2011)	12	18	27	15
Ethiopia (2016)	11	18	26	15
Gambia (2013)	15	20	26	11
Gambia (2019/20)	15	21	28	13
Ghana (2003)	14	21	29	15
Ghana (2008)	13.5	20	30	16.5
Ghana (2014)	13	20	28	15
Guinea (2005)	16	25	30.5	14.5
Guinea (2018)	14	21	29	15
Kenya (2003)	11	17	23	12
Kenya (2008/9)	11	17	25	14
Kenya (2014)	12	18	25	13
Kenya (2022)	10	16	24	14
Lesotho (2009/10)	14	22	31	17
Liberia (2013)	13	19	27	14
Liberia (2019/20)	13	20	29	16
Madagascar (2003/4)	11	17	26	15
Madagascar (2008/9)	12	18	26	14
Madagascar (2021)	10	17	26	16
Malawi (2000)	14	20	27	13
Malawi (2004/5)	15	21	29	14
Malawi (2010)	14	21	28	14
Malawi (2015/16)	15	24	33	18
Mali (2001)	13	19	27	14
Mali (2006)	13	18	25	12
Mali (2012/13)	13	19	28	15
Mali (2018)	12	18	26	14
Mozambique (2003/4)	14	20	27	13
Mozambique (2011)	15	21	27	12
Namibia (2006/7)	14	21	30	16
Niger (2006)	13	19	26	13
Niger (2012)	13	18	25	12
Nigeria (2008)	12	18	26	14
Nigeria (2013)	13	18	25	12
Nigeria (2018)	13	18	26	13

Rwanda (2000)	10	17	25	15
Rwanda (2005)	11	17	23	12
Rwanda (2010/11)	12	18	26	14
Rwanda (2014/15)	12	20	30	18
Rwanda (2019/20)	12	20	30	18
Senegal (2005)	13	18	25	12
Senegal (2010/11)	14	20	27	13
Senegal (2015)	14	19	26	12
Senegal (2016)	14	19	26	12
Senegal (2018)	14	20	27	13
Senegal (2019)	14	20	27	13
Sierra Leone (2008)	13	20	28	15
Sierra Leone (2013)	14	20	28	14
Tanzania (2004/5)	13	19	26	13
Tanzania (2010)	13	18	24	11
Tanzania (2015/16)	12	18	26	14
Uganda (2000/1)	11	16	23	12
Uganda (2006)	11	16	23	12
Uganda (2011)	11	17	23	12
Zambia (2007)	14	19	26	12
Zambia (2013/14)	14	20	28	14
Zambia (2018/19)	15	21	29	14
Zimbabwe (2010/11)	15	24	34	19
Zimbabwe (2015)	13	21	31	18
**North Africa, Western Asia & Europe**				
Egypt (2000)	7	15	23	16
Egypt (2003)	8	16	24	16
Egypt (2005)	8	16	24	16
Egypt (2008)	9	17	25	16
Egypt (2014)	8	15	24	16
Jordan (2002)	6	12	20	14
Jordan (2009)	6	12	20	14
Jordan (2012)	6	13	21	15
Jordan (2017/18)	7	14	24	17
Morocco (2003/4)	10	18	29	19
Türkiye (2003/4)	7	14	22	15
Yemen (2013)	7	14	23	16
**Central, South & Southeast Asia**				
Bangladesh (2004)	10	17	27	17
Bangladesh (2007)	9.5	17	26	17
Bangladesh (2011)	10	18	29	19
Bangladesh (2014)	9	17	29	20
Bangladesh (2017/18)	10	19	31	21
Cambodia (2010/11)	10	17	26	16
India (2005/6)	9	15	23	14
India (2015/16)	9	15	23	14
India (2019/21)	10	16	25	15
Indonesia (2002/3)	11	18	29	18
Indonesia (2007)	9	17	28	19
Indonesia (2012)	8	16	27	19

Indonesia (2017)	8	17	29	21
Myanmar (2015/16)	11	18	29	18
Nepal (2006)	11	17	26	15
Nepal (2011)	11	17	26	15
Nepal (2016/17)	9	16	26	17
Nepal (2021/22)	10	16	26	16
Pakistan (2012/13)	7	14	21	14
Pakistan (2017/18)	8	15	23	15
Philippines (2003)	7	13	21	14
Philippines (2022)	9	15	25	16
Timor-Leste (2009/10)	12	16	23	11
**Latin America & Caribbean**				
Bolivia (2003/4)	10	16	23	13
Bolivia (2008)	10	15	22	12
Colombia (2005)	10	16	25	15
Dominican Republic (2002)	7	14	22	15
Guatemala (2014/15)	12	18	27	15
Honduras (2005/6)	12	18	27	15
Honduras (2011/12)	11	18	28	17
Nicaragua (2001)	9	15	22	13
Peru (2000)	11	17	25	14
Peru (2007/8)	11	17	25	14
Peru (2009)	11	18	27	16

P25=25%; Median=50%; P75=75% of distribution. IQR=Inter-quartile range.

E-Table 4: Perinatal mortality rates (PNMRs) with 95% confidence interval (CI), per 1000 births

Region<br>Survey	PNMR	95% CI		
**sub-Saharan Africa**				
Angola (2015/16)	26.6	(23.2	-	30.5)
Benin (2006)	31.2	(27.5	-	35.3)
Benin (2011/12)	26.0	(22.3	-	30.2)
Benin (2017/18)	31.7	(27.8	-	36.2)
Burkina Faso (2003)	25.9	(21.7	-	30.8)
Burkina Faso (2010)	30.3	(26.5	-	34.6)
Burundi (2010/11)	36.6	(31.3	-	42.7)
Ethiopia (2005)	29.5	(25.0	-	34.8)
Ethiopia (2011)	42.4	(37.2	-	48.3)
Ethiopia (2016)	34.4	(29.6	-	40.0)
Gambia (2013)	27.5	(22.3	-	33.8)
Gambia (2019/20)	46.0	(40.2	-	52.6)
Ghana (2003)	42.3	(33.3	-	53.6)
Ghana (2008)	36.2	(27.0	-	48.3)
Ghana (2014)	34.0	(27.5	-	42.1)
Guinea (2005)	43.6	(36.2	-	52.6)
Guinea (2018)	45.6	(39.0	-	53.2)
Kenya (2003)	44.9	(37.4	-	53.9)
Kenya (2008/9)	34.5	(28.4	-	41.8)
Kenya (2014)	27.1	(22.4	-	32.7)
Kenya (2022)	37.0	(31.3	-	43.8)
Lesotho (2009/10)	42.5	(32.7	-	55.2)
Liberia (2013)	38.4	(31.9	-	46.3)
Liberia (2019/20)	51.1	(42.8	-	61.0)
Madagascar (2003/4)	24.7	(19.4	-	31.4)
Madagascar (2008/9)	26.5	(22.8	-	30.9)
Madagascar (2021)	29.3	(24.6	-	34.8)
Malawi (2000)	38.2	(33.3	-	43.8)
Malawi (2004/5)	33.5	(29.0	-	38.9)
Malawi (2010)	31.9	(28.4	-	35.9)
Malawi (2015/16)	31.2	(27.1	-	35.8)
Mali (2001)	45.0	(39.8	-	50.8)
Mali (2006)	46.7	(41.9	-	51.9)
Mali (2012/13)	36.2	(31.4	-	41.6)
Mali (2018)	40.5	(35.3	-	46.3)

Mozambique (2003/4)	35.7	(30.9	-	41.3)
Mozambique (2011)	36.7	(31.7	-	42.5)
Namibia (2006/7)	27.5	(20.9	-	36.3)
Niger (2006)	32.8	(27.6	-	39.0)
Niger (2012)	31.9	(27.7	-	36.6)
Nigeria (2008)	39.1	(36.1	-	42.4)
Nigeria (2013)	40.3	(37.2	-	43.7)
Nigeria (2018)	48.1	(45.1	-	51.4)
Rwanda (2000)	49.3	(43.1	-	56.5)
Rwanda (2005)	42.4	(36.7	-	48.9)
Rwanda (2010/11)	35.2	(30.1	-	41.2)
Rwanda (2014/15)	29.2	(24.0	-	35.6)
Rwanda (2019/20)	28.8	(23.7	-	35.0)
Senegal (2005)	36.1	(31.2	-	41.9)
Senegal (2010/11)	39.7	(35.2	-	44.8)
Senegal (2015)	36.7	(30.5	-	44.0)
Senegal (2016)	30.8	(25.0	-	37.9)
Senegal (2018)	37.3	(30.9	-	44.9)
Senegal (2019)	36.3	(29.8	-	44.4)
Sierra Leone (2008)	36.2	(29.0	-	45.0)
Sierra Leone (2013)	39.1	(34.0	-	45.0)
Tanzania (2004/5)	40.4	(35.0	-	46.7)
Tanzania (2010)	35.4	(29.5	-	42.5)
Tanzania (2015/16)	35.2	(30.3	-	40.9)
Uganda (2000/1)	36.4	(31.1	-	42.6)
Uganda (2006)	32.1	(27.3	-	37.8)
Uganda (2011)	34.6	(29.5	-	40.6)
Zambia (2007)	30.3	(24.6	-	37.4)
Zambia (2013/14)	31.6	(27.5	-	36.2)
Zambia (2018/19)	34.6	(29.2	-	40.9)
Zimbabwe (2010/11)	34.2	(26.8	-	43.5)
Zimbabwe (2015)	30.6	(23.9	-	39.2)
**North Africa Western Asia & Europe**				
Egypt (2000)	32.1	(27.3	-	37.8)
Egypt (2003)	26.8	(21.2	-	33.8)
Egypt (2005)	25.8	(21.8	-	30.5)
Egypt (2008)	20.0	(15.9	-	25.1)
Egypt (2014)	16.4	(13.4	-	20.0)
Jordan (2002)	22.7	(18.1	-	28.5)
Jordan (2009)	22.9	(19.2	-	27.4)
Jordan (2012)	16.7	(13.7	-	20.5)
Jordan (2017/18)	10.4	(7.9	-	13.6)
Morocco (2003/4)	35.3	(28.7	-	43.3)

Türkiye (2003/4)	28.5	(22.2	-	36.6)
Yemen (2013)	33.9	(30.2	-	38.1)
**Central, South & Southeast Asia**				
Bangladesh (2004)	56.9	(48.8	-	66.4)
Bangladesh (2007)	49.3	(40.2	-	60.4)
Bangladesh (2011)	40.4	(33.2	-	49.1)
Bangladesh (2014)	43.5	(35.1	-	53.9)
Bangladesh (2017/18)	40.1	(32.9	-	48.8)
Cambodia (2010/11)	29.4	(23.9	-	36.1)
India (2005/6)	40.4	(37.9	-	43.1)
India (2015/16)	35.2	(34.0	-	36.4)
India (2019/21)	29.5	(28.4	-	30.7)
Indonesia (2002/3)	25.7	(21.5	-	30.7)
Indonesia (2007)	27.0	(22.8	-	31.9)
Indonesia (2012)	28.8	(24.1	-	34.4)
Indonesia (2017)	19.8	(16.0	-	24.4)
Myanmar (2015/16)	32.3	(24.8	-	42.1)
Nepal (2006)	50.1	(42.5	-	59.0)
Nepal (2011)	40.2	(32.7	-	49.4)
Nepal (2016/17)	36.2	(28.1	-	46.5)
Nepal (2021/22)	29.4	(22.7	-	38.2)
Pakistan (2012/13)	67.6	(61.9	-	73.8)
Pakistan (2017/18)	54.8	(49.2	-	61.0)
Philippines (2003)	25.5	(20.5	-	31.8)
Philippines (2022)	23.6	(18.0	-	31.0)
Timor-Leste (2009/10)	20.1	(16.6	-	24.3)
**Latin America & Caribbean**				
Bolivia (2003/4)	28.3	(24.0	-	33.5)
Bolivia (2008)	27.4	(22.0	-	34.0)
Colombia (2005)	15.0	(12.1	-	18.7)
Dominican Republic (2002)	23.9	(20.0	-	28.7)
Guatemala (2014/15)	20.9	(17.3	-	25.3)
Honduras (2005/6)	22.5	(18.7	-	27.1)
Honduras (2011/12)	21.2	(17.0	-	26.3)
Nicaragua (2001)	20.1	(15.5	-	26.0)
Peru (2000)	22.4	(18.6	-	27.0)
Peru (2007/8)	18.6	(14.0	-	24.6)
Peru (2009)	15.8	(11.8	-	21.2)

*Note the rates are based on women who had at least one index pregnancy during the analysis period.

E-Figure 1: Survey-specific Kaplan-Meier survival probabilities, by IPI group (in months)

E-Figure 1.1: Angola (2015/16)

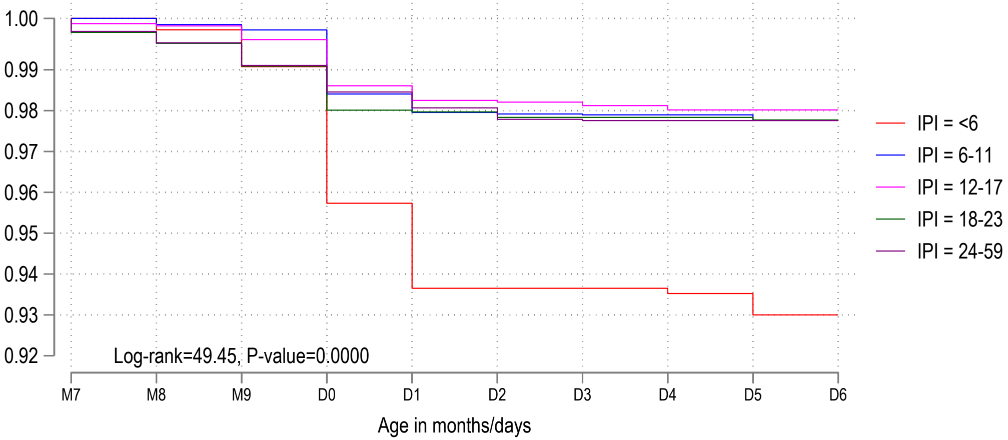

Number at risk

IPI = <6	680	670	658	647	625	611	611	611	610	607
IPI = 6-11	983	977	967	959	943	939	939	939	939	937
IPI = 12-17	1888	1866	1828	1812	1791	1785	1784	1782	1780	1780
IPI = 18-23	1584	1552	1509	1486	1469	1469	1467	1467	1467	1466
IPI = 24-59	2151	2071	2001	1963	1950	1942	1937	1936	1936	1936

E-Figure 1.2: Benin (2006)

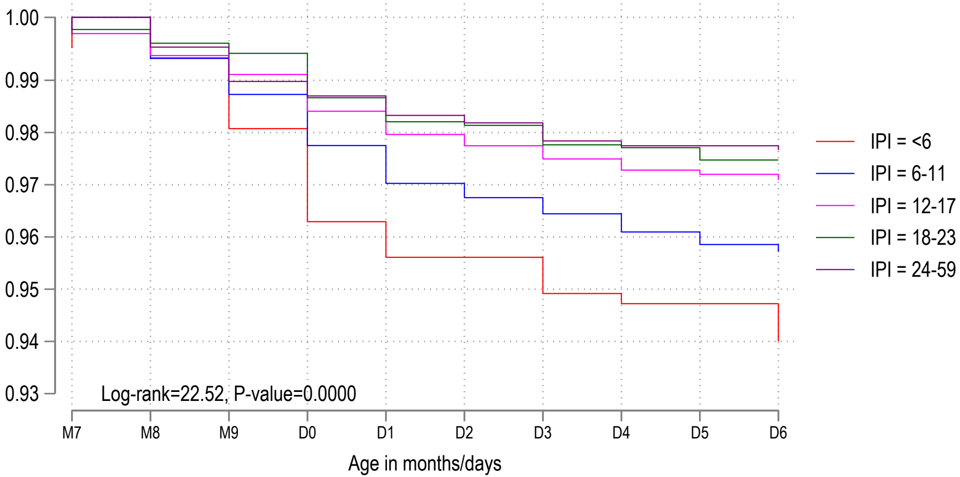

Number at risk

IPI = <6	459	451	440	428	420	417	417	414	413	413
IPI = 6-11	795	774	758	745	738	733	731	728	726	724
IPI = 12-17	1548	1522	1485	1468	1457	1451	1447	1444	1440	1439
IPI = 18-23	1940	1894	1843	1821	1806	1797	1796	1789	1788	1783
IPI = 24-59	3028	2899	2795	2741	2733	2723	2719	2710	2707	2707

E-Figure 1.3: Benin (2011/12)

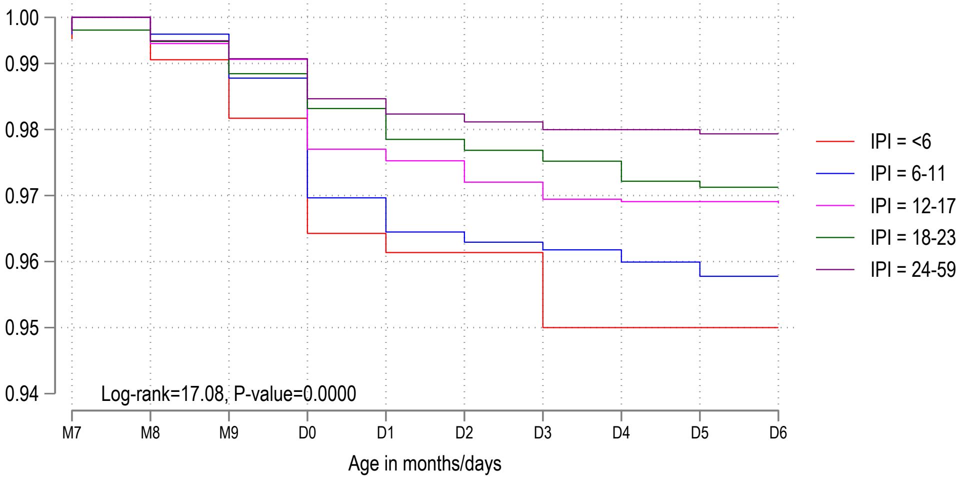

Number at risk

IPI = <6	326	323	320	307	302	301	301	297	297	297
IPI = 6-11	655	634	628	621	609	606	605	604	603	602
IPI = 12-17	1338	1317	1302	1277	1260	1257	1253	1250	1249	1249
IPI = 18-23	1403	1371	1342	1326	1319	1313	1311	1308	1304	1303
IPI = 24-59	2633	2552	2488	2441	2426	2420	2418	2415	2415	2413

E-Figure 1.4: Benin (2017/18)

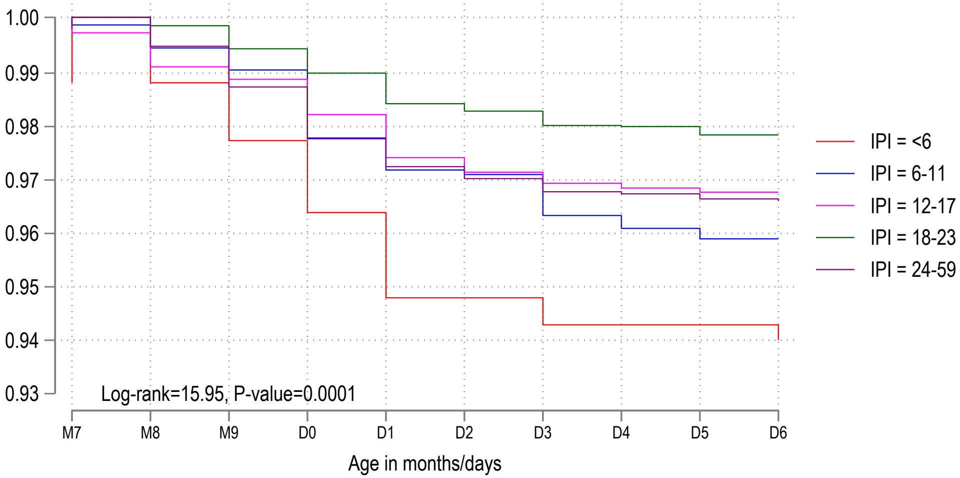

Number at risk

IPI = <6	333	327	321	316	311	306	306	305	305	305
IPI = 6-11	683	672	659	657	648	644	644	639	637	636
IPI = 12-17	1501	1477	1450	1441	1432	1420	1416	1413	1412	1411
IPI = 18-23	1736	1704	1667	1646	1638	1629	1626	1622	1621	1619
IPI = 24-59	2783	2679	2593	2550	2525	2511	2506	2499	2498	2496

E-Figure 1.5: Burkina Faso (2003)

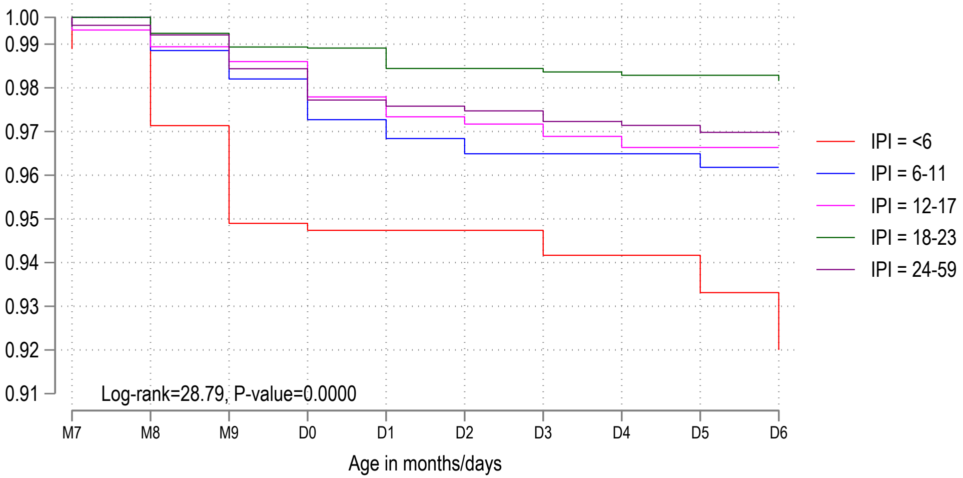

Number at risk

IPI = <6	243	236	229	224	223	223	223	222	222	220
IPI = 6-11	492	482	475	467	462	460	459	459	459	457
IPI = 12-17	908	892	862	857	850	846	844	842	840	840
IPI = 18-23	1101	1080	1063	1050	1050	1045	1045	1044	1043	1043
IPI = 24-59	2457	2358	2244	2206	2190	2187	2184	2179	2177	2173

E-Figure 1.6: Burkina Faso (2010)

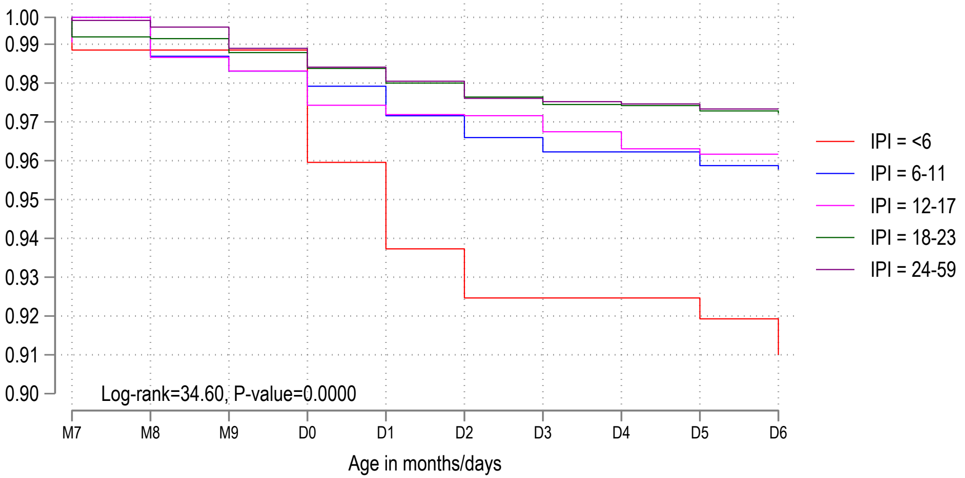

Number at risk

IPI = <6	234	230	221	221	215	210	207	207	207	206
IPI = 6-11	647	637	624	621	618	614	610	608	608	606
IPI = 12-17	1409	1375	1334	1327	1315	1312	1311	1306	1300	1298
IPI = 18-23	1793	1737	1697	1676	1670	1663	1657	1654	1653	1651
IPI = 24-59	3219	3112	2960	2927	2912	2902	2889	2886	2884	2881

E-Figure 1.7: Burundi (2010/11)

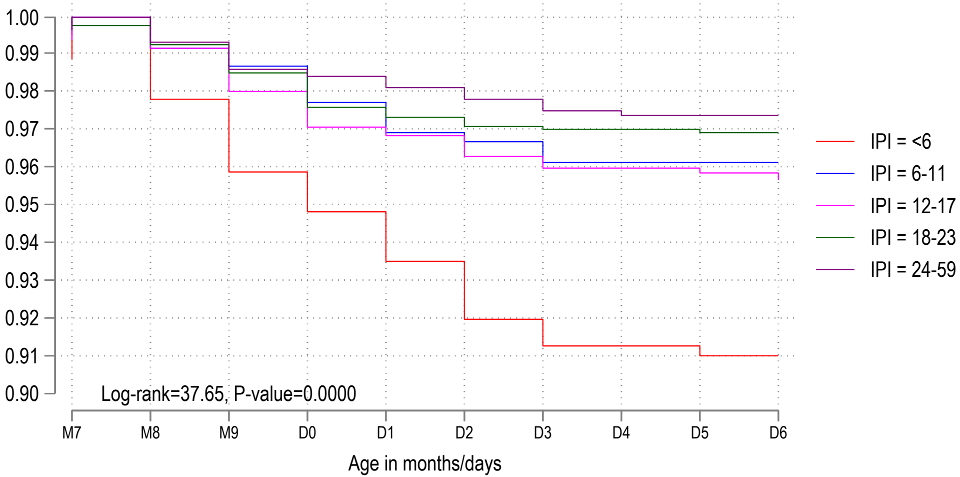

Number at risk

IPI = <6	445	434	418	408	404	398	392	389	389	388
IPI = 6-11	584	574	564	561	556	551	550	547	547	547
IPI = 12-17	1094	1062	1030	1014	1005	1002	997	994	994	992
IPI = 18-23	1061	1033	980	968	959	956	954	953	953	952
IPI = 24-59	1341	1293	1229	1216	1213	1210	1206	1202	1201	1201

E-Figure 1.8: Ethiopia (2005)

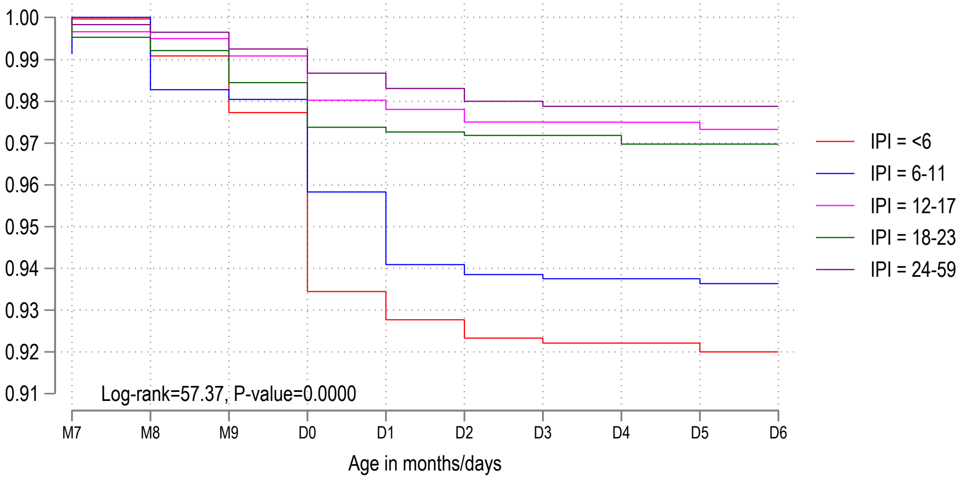

Number at risk

IPI = <6	410	401	387	380	363	361	359	358	358	358
IPI = 6-11	764	738	722	713	697	684	682	681	681	681
IPI = 12-17	1157	1131	1103	1086	1074	1072	1069	1069	1068	1067
IPI = 18-23	1117	1087	1055	1033	1022	1021	1020	1020	1018	1018
IPI = 24-59	2272	2195	2089	2040	2028	2020	2014	2012	2012	2012

E-Figure 1.9: Ethiopia (2011)

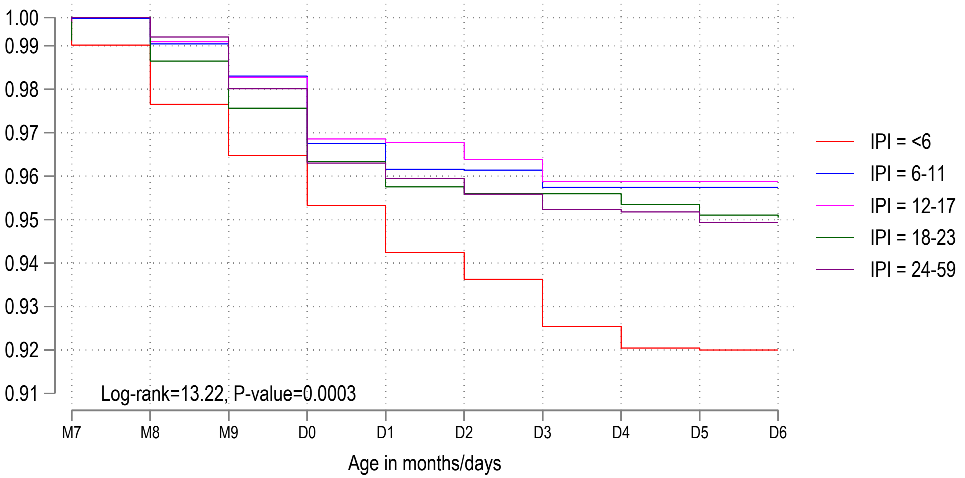

Number at risk

IPI = <6	529	513	501	491	485	480	477	471	469	468
IPI = 6-11	753	749	731	714	703	698	698	695	695	695
IPI = 12-17	1056	1034	1009	999	985	984	980	975	975	975
IPI = 18-23	1065	1023	984	968	956	950	949	949	946	944
IPI = 24-59	1984	1894	1822	1769	1739	1732	1726	1719	1718	1714

E-Figure 1.10: Ethiopia (2016)

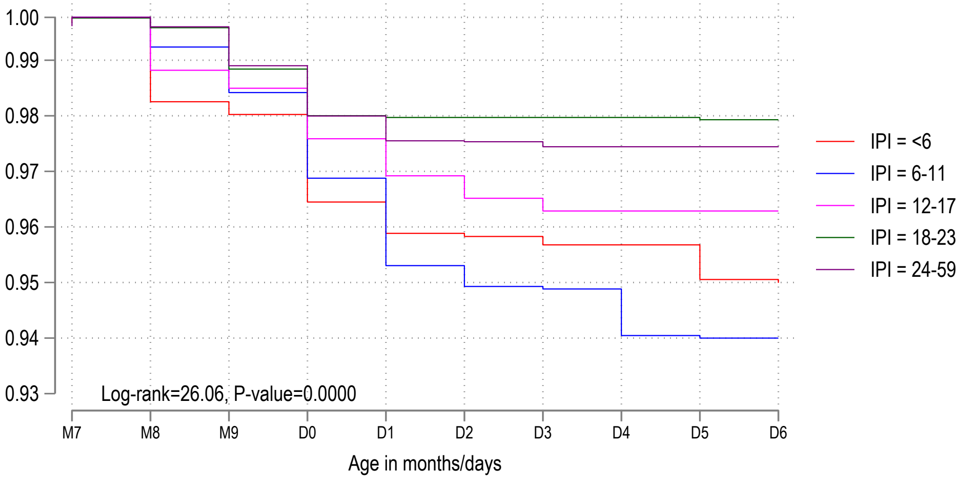

Number at risk

IPI = <6	517	503	492	490	483	480	480	479	479	476
IPI = 6-11	642	635	620	609	599	590	587	587	582	582
IPI = 12-17	1081	1075	1045	1031	1022	1015	1011	1008	1008	1008
IPI = 18-23	914	884	845	820	813	813	813	813	813	812
IPI = 24-59	1775	1711	1651	1605	1590	1583	1582	1581	1581	1581

E-Figure 1.11: Gambia (2013)

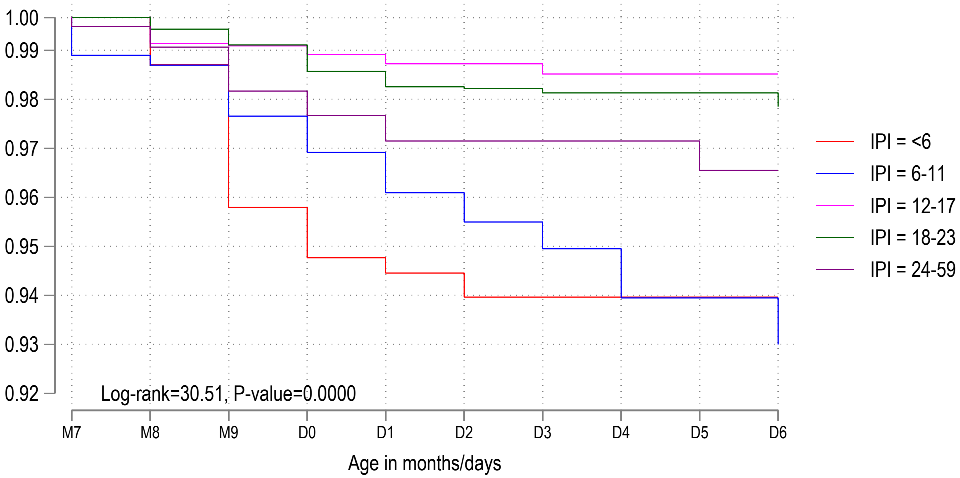

Number at risk

IPI = <6	155	153	149	144	142	142	141	141	141	141
IPI = 6-11	294	283	276	270	268	265	264	262	259	259
IPI = 12-17	718	699	674	668	667	665	665	664	664	664
IPI = 18-23	878	862	836	821	817	814	814	813	813	813
IPI = 24-59	1146	1093	1030	1008	1003	998	998	998	998	992

E-Figure 1.12: Gambia (2019/20)

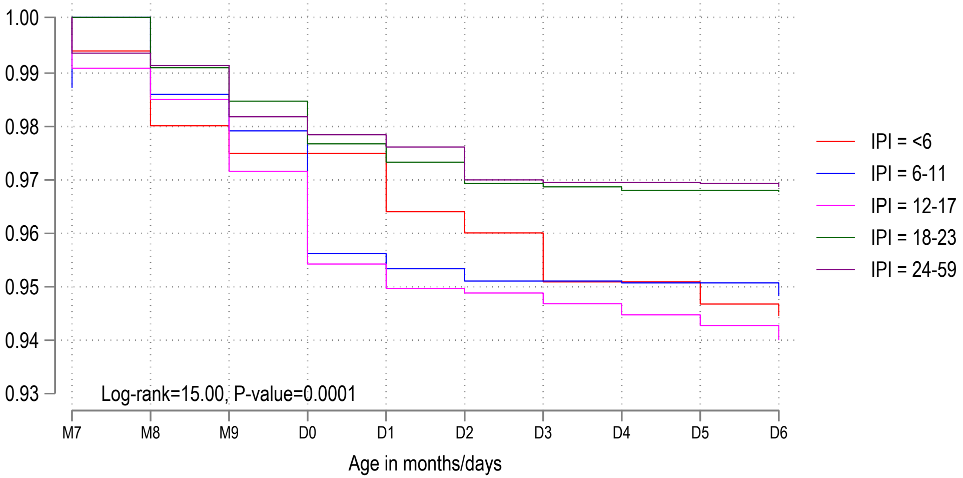

Number at risk

IPI = <6	261	253	243	238	238	235	234	232	232	229
IPI = 6-11	410	402	389	380	371	370	369	369	369	369
IPI = 12-17	819	797	777	752	738	735	734	733	731	730
IPI = 18-23	960	936	912	892	885	882	878	878	877	877
IPI = 24-59	1661	1603	1546	1508	1502	1499	1489	1489	1489	1488

E-Figure 1.13: Ghana (2003)

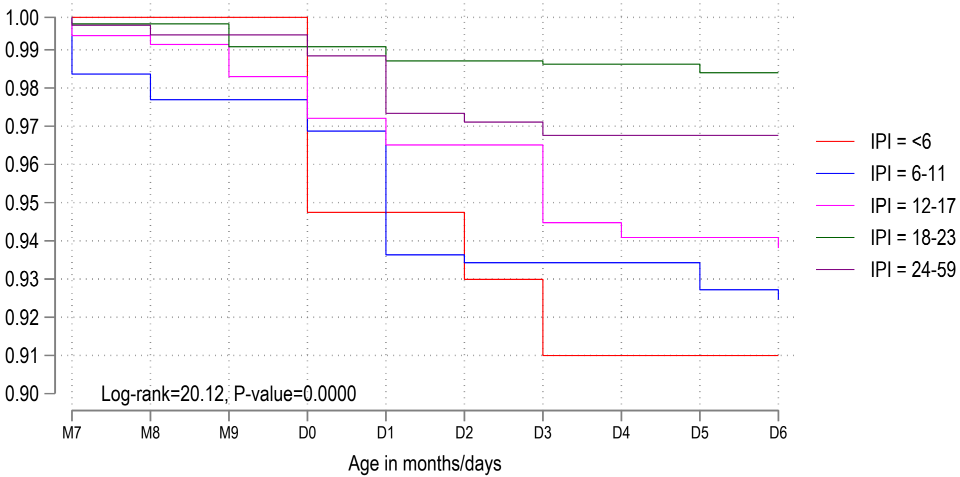

Number at risk

IPI = <6	126	122	121	121	115	115	113	111	111	111
IPI = 6-11	157	149	147	145	144	139	138	138	138	137
IPI = 12-17	294	286	280	276	273	271	271	265	264	264
IPI = 18-23	330	329	320	311	311	310	310	310	310	309
IPI = 24-59	606	581	555	539	536	528	527	525	525	525

E-Figure 1.14: Ghana (2008)

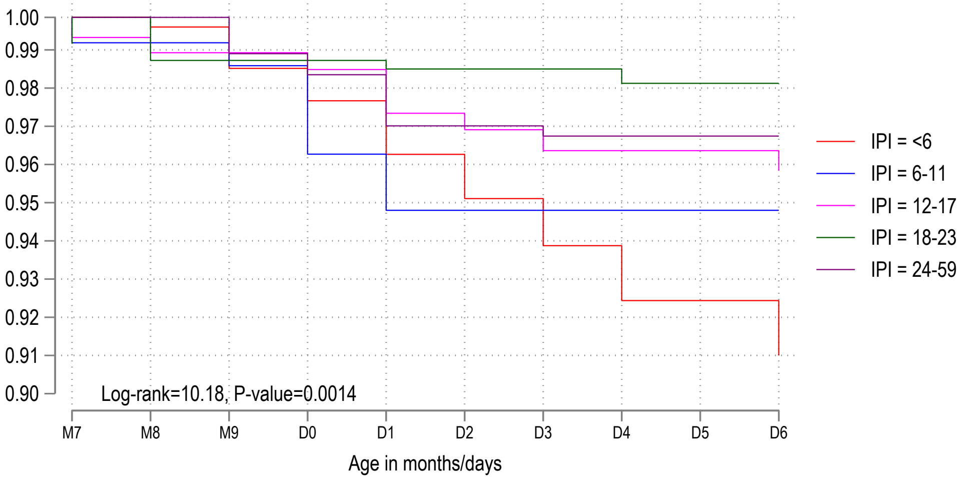

Number at risk

IPI = <6	93	92	89	86	85	84	83	82	81	81
IPI = 6-11	140	136	136	135	131	129	129	129	129	129
IPI = 12-17	256	251	245	245	244	241	240	239	239	239
IPI = 18-23	261	250	244	241	241	241	241	241	240	240
IPI = 24-59	481	463	450	440	437	431	431	430	430	430

E-Figure 1.15: Ghana (2014)

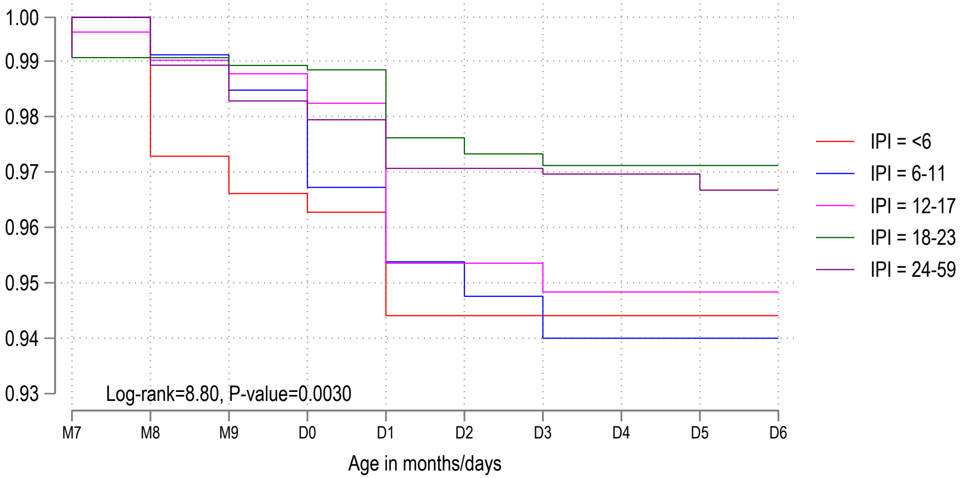

Number at risk

IPI = <6	226	219	211	208	208	204	204	204	204	204
IPI = 6-11	353	348	342	339	333	329	327	324	324	324
IPI = 12-17	533	522	505	502	499	484	484	482	482	482
IPI = 18-23	513	499	487	485	485	479	477	476	476	476
IPI = 24-59	850	819	779	771	768	761	761	760	760	758

E-Figure 1.16: Guinea (2005)

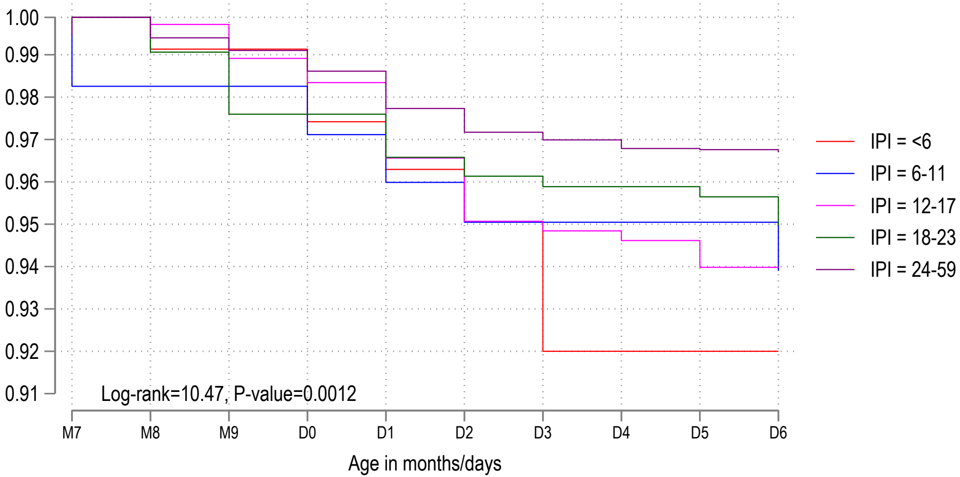

Number at risk

IPI = <6	105	97	91	88	87	86	85	82	82	82
IPI = 6-11	170	165	162	160	158	156	155	155	155	155
IPI = 12-17	468	460	441	432	430	422	416	415	414	411
IPI = 18-23	470	451	441	427	427	422	420	419	419	418
IPI = 24-59	1411	1361	1286	1256	1250	1239	1232	1230	1227	1227

E-Figure 1.17: Guinea (2018)

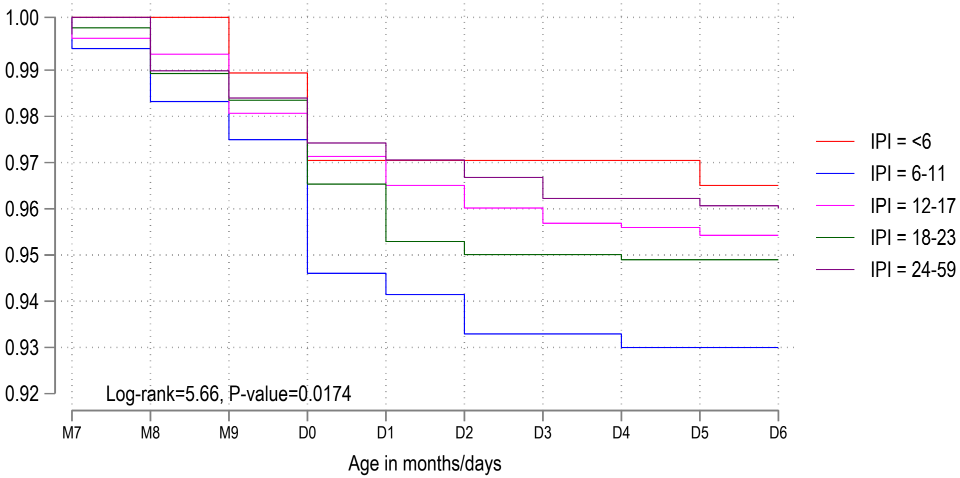

Number at risk

IPI = <6	221	218	216	212	208	208	208	208	208	207
IPI = 6-11	290	282	273	269	261	259	257	257	256	256
IPI = 12-17	866	857	843	828	820	814	810	807	807	805
IPI = 18-23	621	607	588	578	568	560	559	559	558	558
IPI = 24-59	1461	1412	1354	1310	1297	1292	1287	1281	1281	1279

E-Figure 1.18: Kenya (2003)

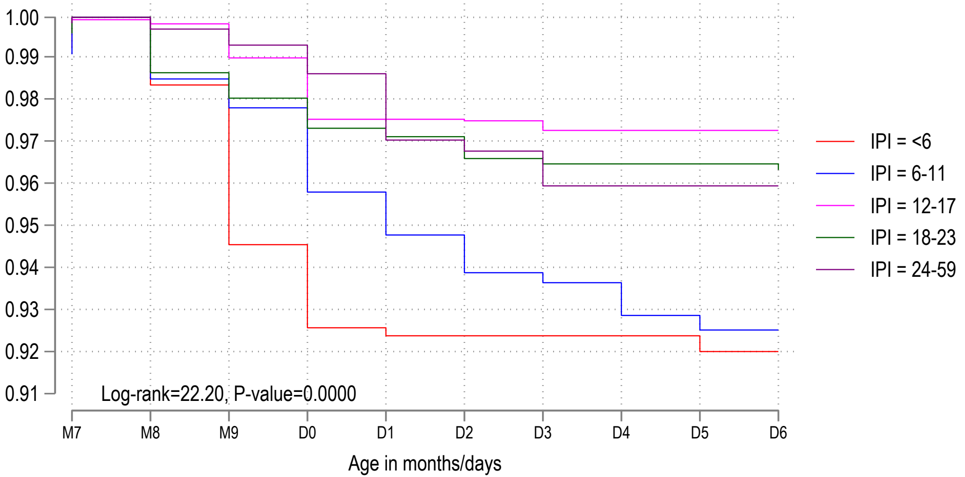

Number at risk

IPI = <6	275	272	264	253	247	247	247	247	247	246
IPI = 6-11	463	443	432	426	417	412	409	408	404	403
IPI = 12-17	649	634	620	610	601	601	601	600	600	600
IPI = 18-23	609	596	566	557	553	552	549	549	549	549
IPI = 24-59	659	641	604	591	587	577	576	571	571	571

E-Figure 1.19: Kenya (2008/9)

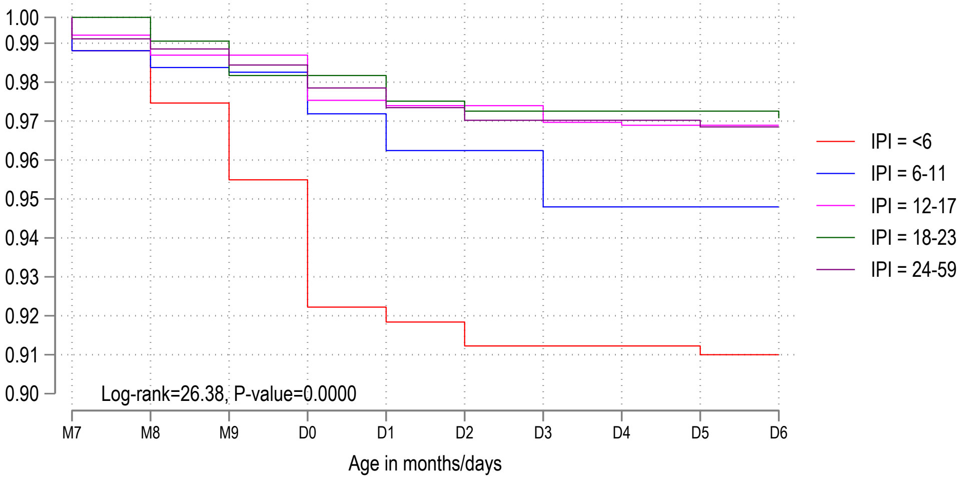

Number at risk

IPI = <6	276	268	261	255	247	246	244	244	244	243
IPI = 6-11	455	446	440	440	435	431	431	424	424	424
IPI = 12-17	695	681	663	661	653	652	652	649	649	649
IPI = 18-23	569	565	542	534	534	530	529	529	529	529
IPI = 24-59	872	851	822	813	808	804	801	801	801	800

E-Figure 1.20: Kenya (2014)

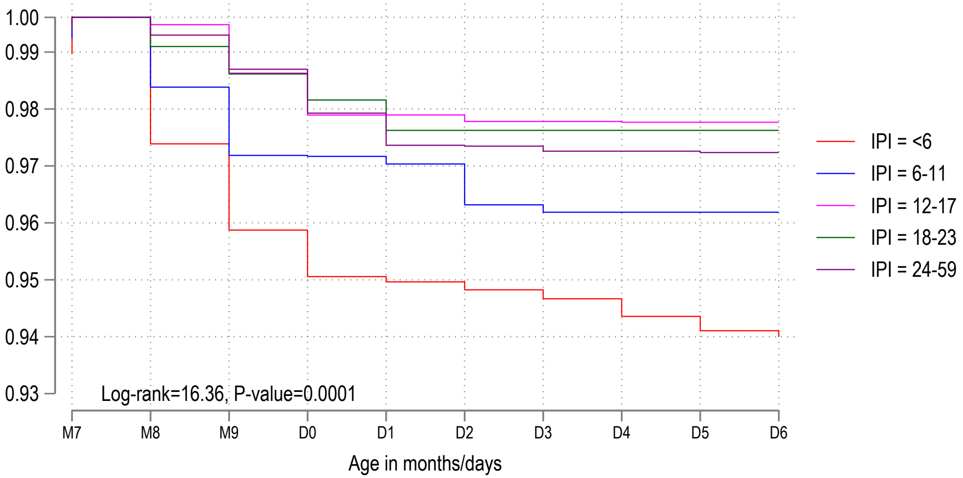

Number at risk

IPI = <6	333	322	312	302	299	299	298	298	297	296
IPI = 6-11	518	505	494	485	485	484	481	480	480	480
IPI = 12-17	848	830	813	805	799	799	798	798	798	798
IPI = 18-23	747	733	714	711	708	704	704	704	704	704
IPI = 24-59	1056	1008	956	943	935	930	930	929	929	929

E-Figure 1.21: Kenya (2022)

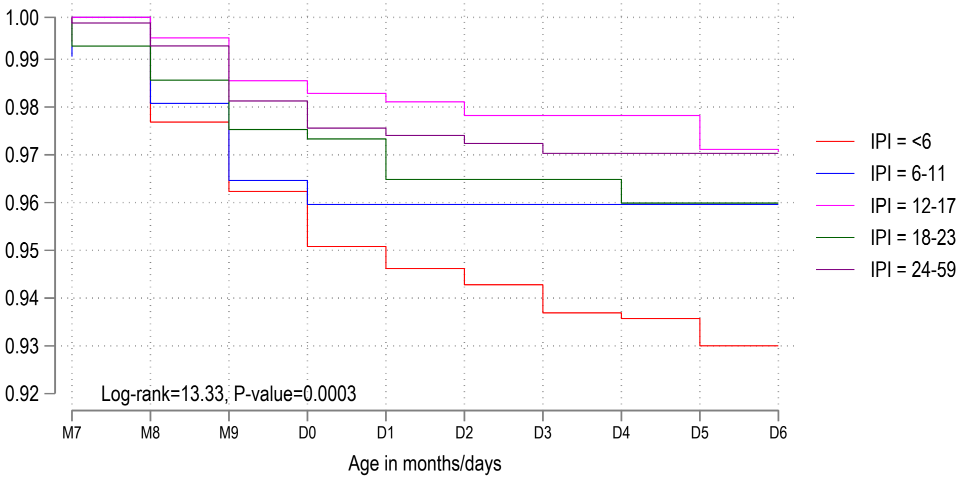

Number at risk

IPI = <6	372	367	355	348	344	343	341	339	339	337
IPI = 6-11	479	468	457	447	444	444	444	444	444	444
IPI = 12-17	599	584	577	566	564	563	562	562	562	558
IPI = 18-23	493	483	470	459	458	454	454	454	452	452
IPI = 24-59	854	809	743	723	719	718	716	715	715	715

E-Figure 1.22: Lesotho (2009/10)

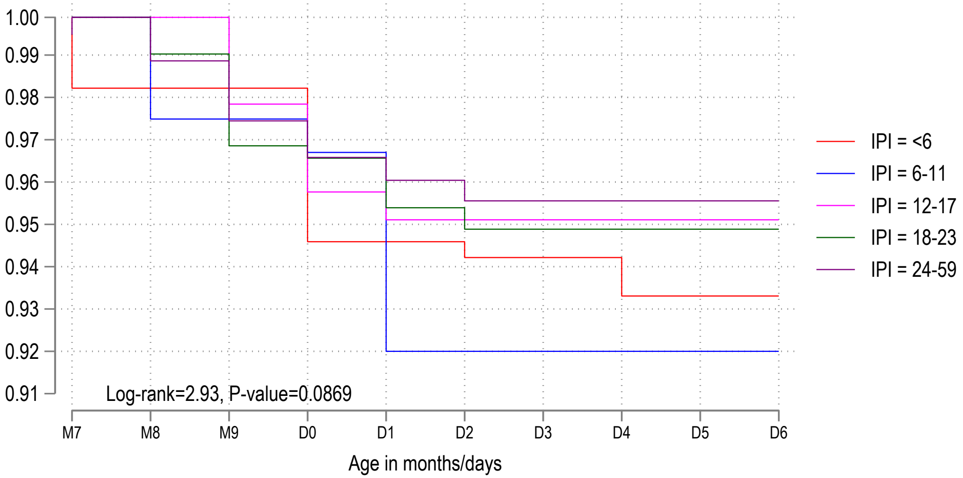

Number at risk

IPI = <6	99	96	96	95	92	92	91	91	90	90
IPI = 6-11	120	120	116	114	113	108	108	108	108	108
IPI = 12-17	224	220	217	211	207	205	205	205	205	205
IPI = 18-23	186	183	181	176	175	173	172	172	172	172
IPI = 24-59	535	515	498	487	483	480	478	478	478	477

E-Figure 1.23: Liberia (2013)

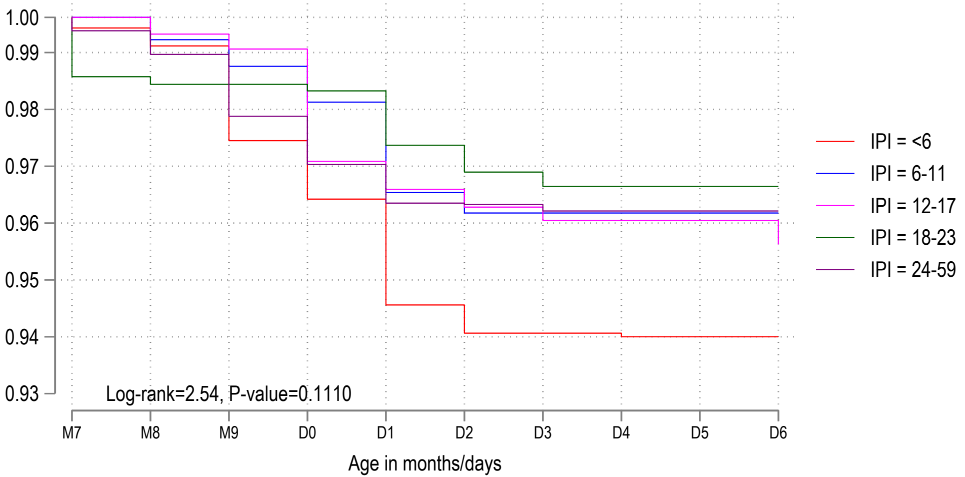

Number at risk

IPI = <6	165	161	158	150	148	146	145	145	145	145
IPI = 6-11	310	302	293	291	289	284	283	283	283	283
IPI = 12-17	508	498	483	479	469	467	465	464	464	464
IPI = 18-23	482	468	457	446	446	441	439	438	438	438
IPI = 24-59	852	817	757	716	709	705	704	704	704	704

E-Figure 1.24: Liberia (2019/20)

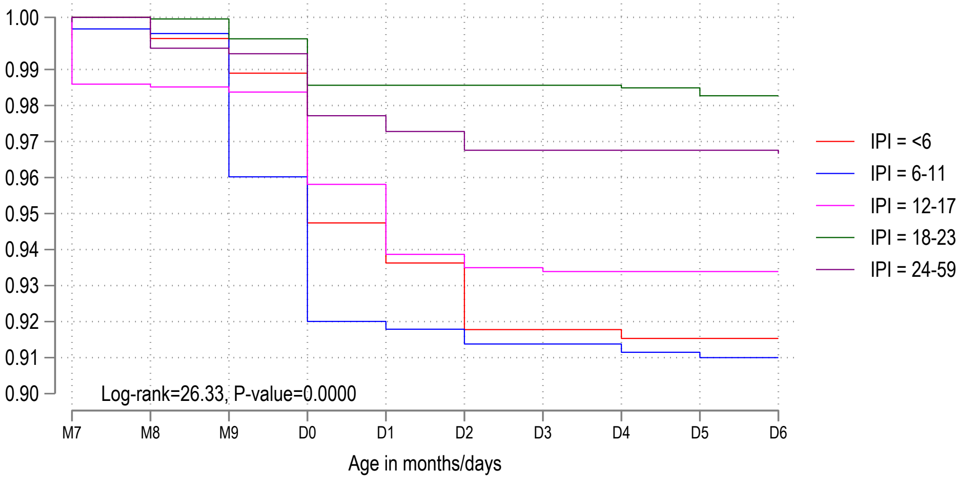

Number at risk

IPI = <6	152	140	138	136	130	129	126	126	126	126
IPI = 6-11	233	230	229	216	207	206	205	205	205	204
IPI = 12-17	402	392	387	383	373	365	364	363	363	363
IPI = 18-23	426	422	412	401	395	395	395	395	395	394
IPI = 24-59	832	802	781	757	744	740	736	736	736	736

E-Figure 1.25: Madagascar (2003/4)

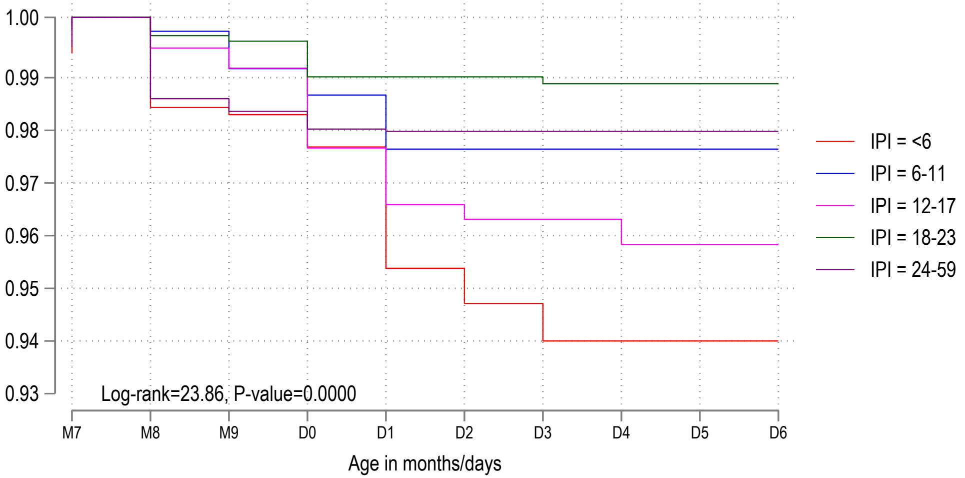

Number at risk

IPI = <6	363	353	346	345	343	335	333	330	330	330
IPI = 6-11	509	496	486	478	476	471	471	471	471	471
IPI = 12-17	847	815	792	781	769	761	758	758	755	755
IPI = 18-23	566	555	540	531	527	527	527	526	526	526
IPI = 24-59	1034	1001	967	940	937	937	937	937	937	937

E-Figure 1.26: Madagascar (2008/9)

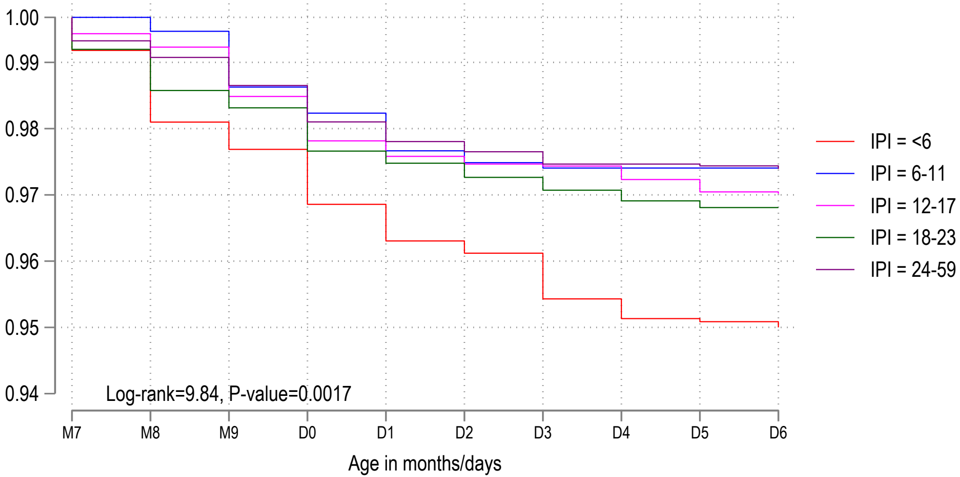

Number at risk

IPI = <6	537	526	513	507	502	500	499	495	493	492
IPI = 6-11	1025	1015	997	984	981	975	973	972	972	972
IPI = 12-17	1619	1588	1550	1532	1521	1518	1516	1515	1512	1509
IPI = 18-23	1351	1315	1275	1264	1255	1253	1250	1248	1246	1244
IPI = 24-59	1858	1792	1722	1704	1694	1689	1686	1683	1683	1683

E-Figure 1.27: Madagascar (2021)

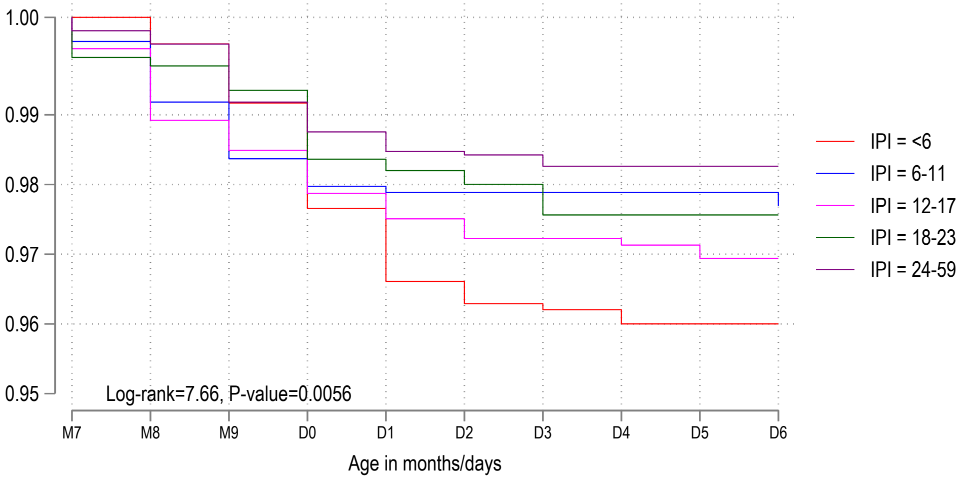

Number at risk

IPI = <6	551	544	530	523	515	509	508	507	506	506
IPI = 6-11	783	774	750	741	738	738	738	738	738	738
IPI = 12-17	961	946	915	906	900	897	894	894	893	892
IPI = 18-23	716	701	674	665	658	657	656	653	653	653
IPI = 24-59	1309	1263	1197	1174	1169	1166	1165	1164	1164	1164

E-Figure 1.28: Malawi (2000)

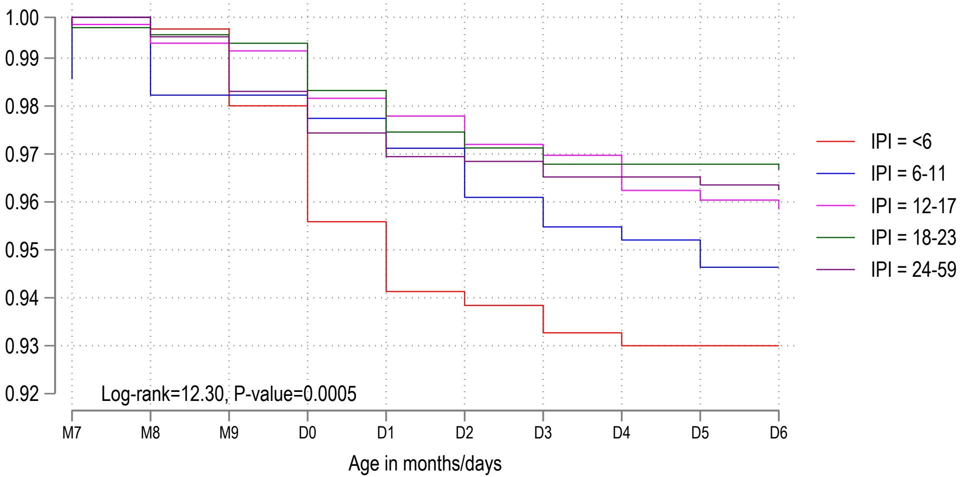

Number at risk

IPI = <6	338	326	316	310	302	297	297	295	294	294
IPI = 6-11	692	667	652	651	648	643	637	633	631	627
IPI = 12-17	1363	1330	1293	1278	1265	1260	1252	1250	1240	1238
IPI = 18-23	1290	1238	1200	1176	1165	1155	1151	1147	1147	1147
IPI = 24-59	1997	1897	1818	1771	1755	1746	1745	1739	1739	1736

E-Figure 1.29: Malawi (2004/5)

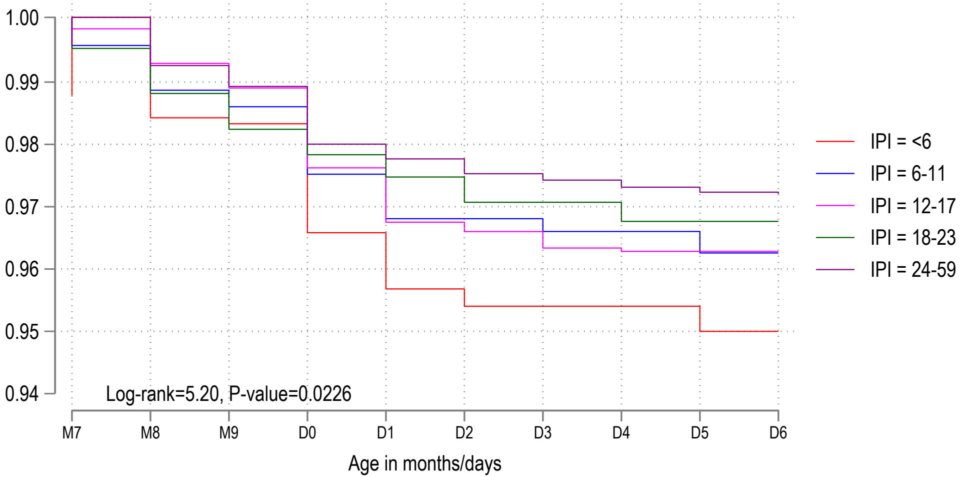

Number at risk

IPI = <6	310	302	294	293	288	285	284	284	284	283
IPI = 6-11	538	524	509	503	497	494	494	493	493	491
IPI = 12-17	1035	1017	993	976	964	955	954	951	950	949
IPI = 18-23	1089	1057	1019	1004	999	996	992	992	989	989
IPI = 24-59	2242	2152	2054	2023	2004	1999	1994	1992	1990	1988

E-Figure 1.30: Malawi (2010)

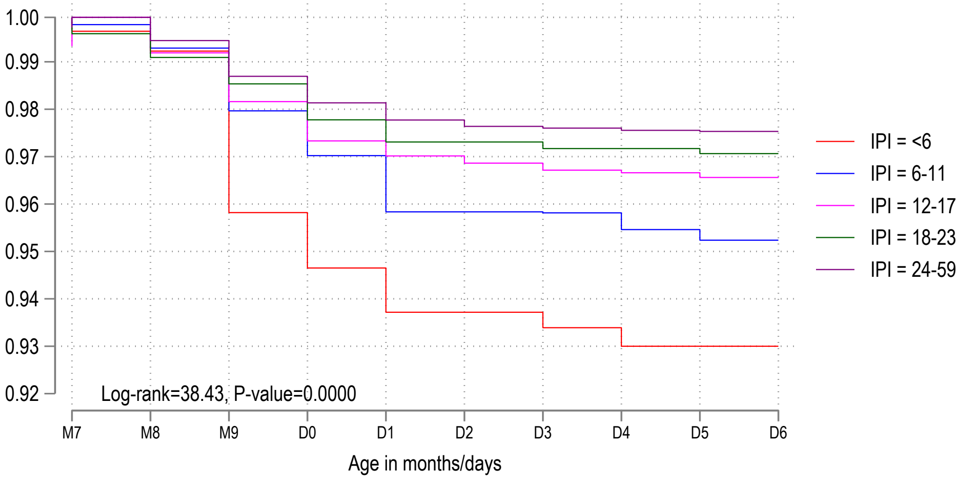

Number at risk

IPI = <6	599	590	578	554	548	542	542	540	538	538
IPI = 6-11	949	935	920	903	894	883	883	883	880	878
IPI = 12-17	1823	1787	1758	1730	1715	1710	1707	1704	1703	1700
IPI = 18-23	1785	1727	1677	1659	1646	1638	1638	1636	1636	1634
IPI = 24-59	3299	3170	3065	3008	2991	2980	2976	2975	2973	2973

E-Figure 1.31: Malawi (2015/16)

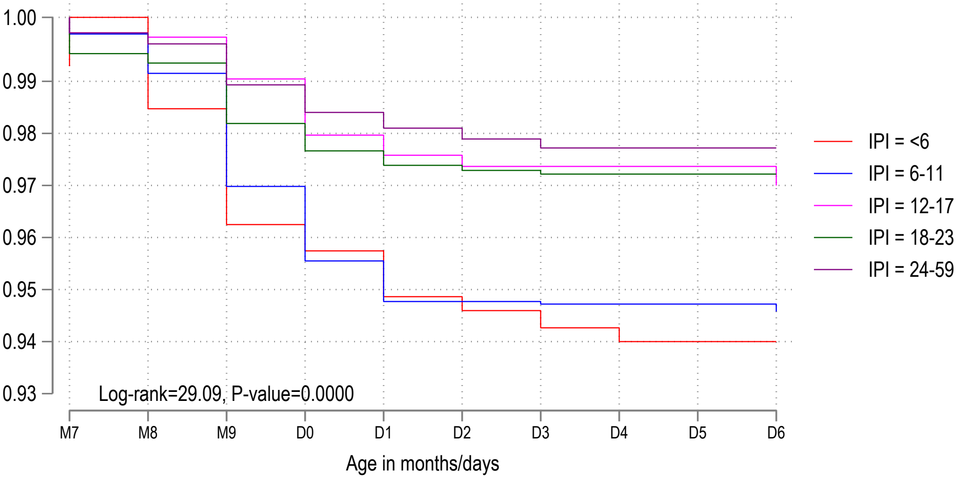

Number at risk

IPI = <6	450	437	428	412	409	406	404	403	402	402
IPI = 6-11	616	608	594	576	568	563	563	563	563	563
IPI = 12-17	949	937	921	903	893	890	888	888	888	888
IPI = 18-23	1195	1171	1146	1120	1114	1111	1110	1109	1109	1109
IPI = 24-59	3276	3159	3038	2970	2955	2945	2939	2934	2934	2934

E-Figure 1.32: Mali (2001)

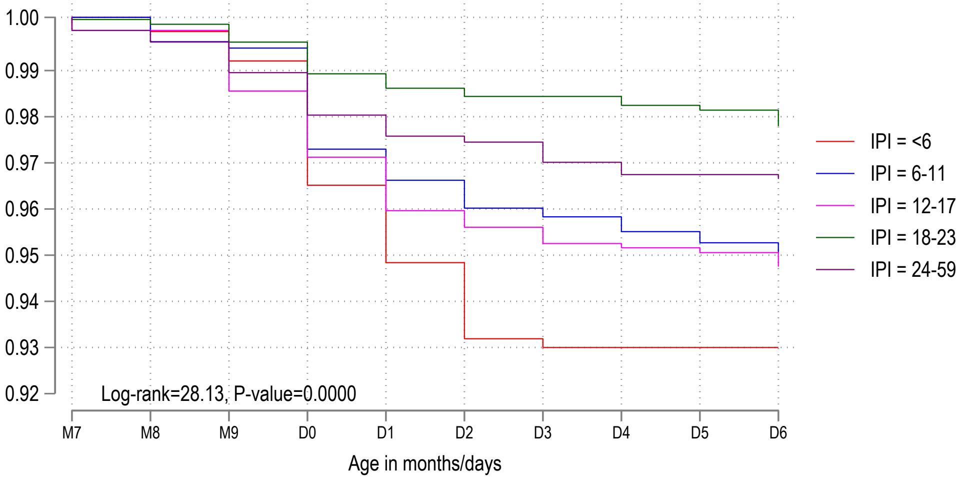

Number at risk

IPI = <6	499	486	477	468	455	447	439	439	439	439
IPI = 6-11	737	719	698	690	675	670	666	665	663	661
IPI = 12-17	1454	1424	1400	1369	1349	1333	1328	1323	1322	1321
IPI = 18-23	1241	1206	1166	1148	1140	1136	1134	1134	1132	1131
IPI = 24-59	2136	2028	1903	1852	1835	1826	1824	1816	1811	1811

E-Figure 1.33: Mali (2006)

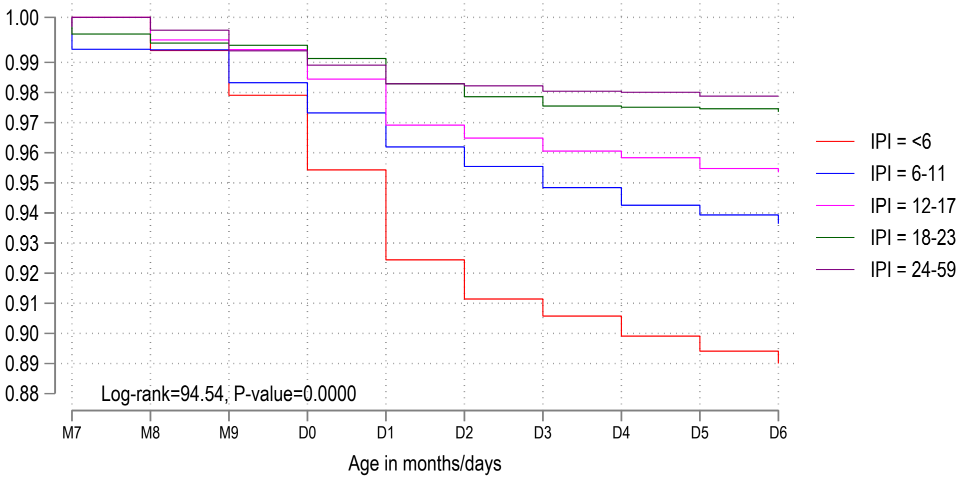

Number at risk

IPI = <6	578	560	533	518	505	489	482	479	475	473
IPI = 6-11	986	956	938	916	907	896	890	883	878	875
IPI = 12-17	1997	1958	1910	1882	1864	1835	1826	1818	1814	1807
IPI = 18-23	1633	1584	1537	1516	1509	1496	1490	1485	1484	1483
IPI = 24-59	2240	2149	2056	1996	1986	1974	1972	1969	1968	1965

E-Figure 1.34: Mali (2012/13)

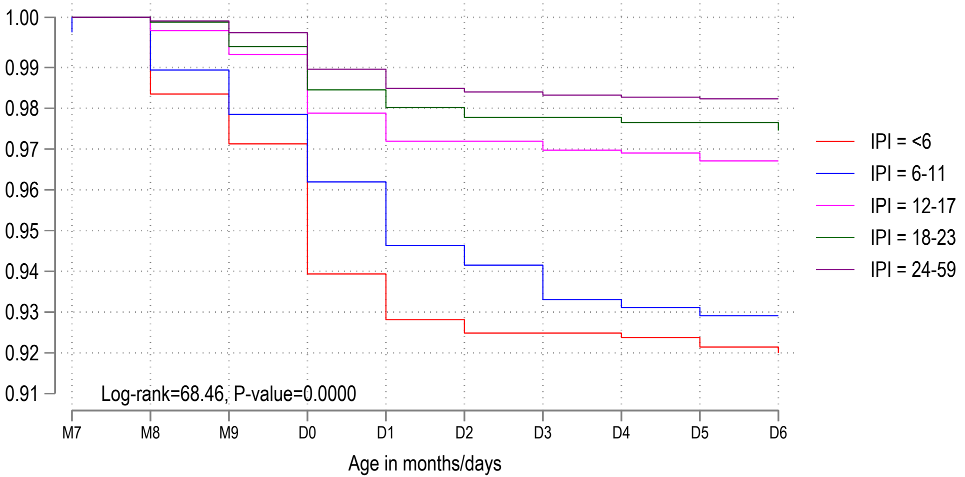

Number at risk

IPI = <6	428	424	415	407	393	388	387	387	387	386
IPI = 6-11	692	679	660	646	635	624	621	616	614	613
IPI = 12-17	1247	1234	1208	1193	1176	1168	1168	1165	1164	1162
IPI = 18-23	1151	1134	1105	1093	1082	1077	1074	1074	1073	1073
IPI = 24-59	1991	1920	1860	1827	1811	1802	1800	1799	1798	1797

E-Figure 1.35: Mali (2018)

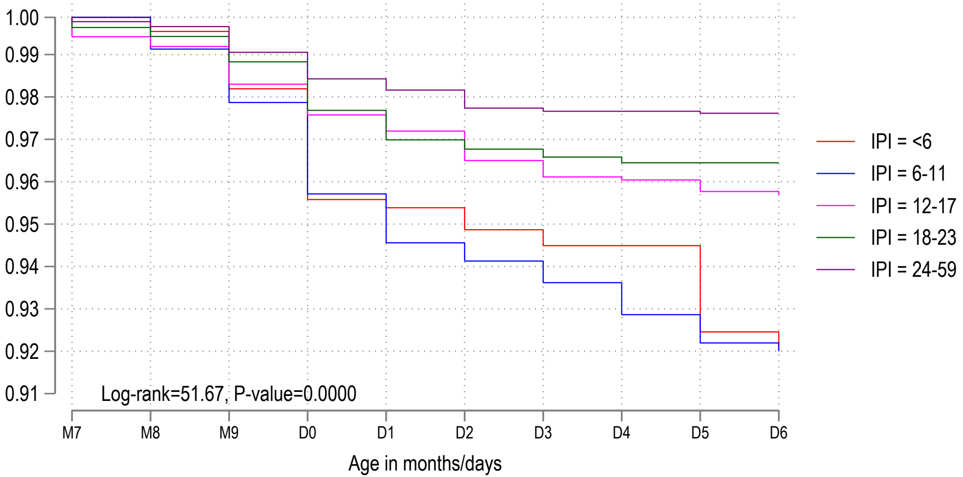

Number at risk

IPI = <6	385	380	372	367	357	357	355	353	353	345
IPI = 6-11	713	706	686	672	658	650	647	643	638	633
IPI = 12-17	1321	1294	1262	1245	1236	1231	1222	1217	1216	1213
IPI = 18-23	1165	1129	1100	1084	1072	1064	1062	1060	1058	1058
IPI = 24-59	1734	1688	1613	1581	1571	1567	1560	1559	1559	1558

E-Figure 1.36: Mozambique (2003/4)

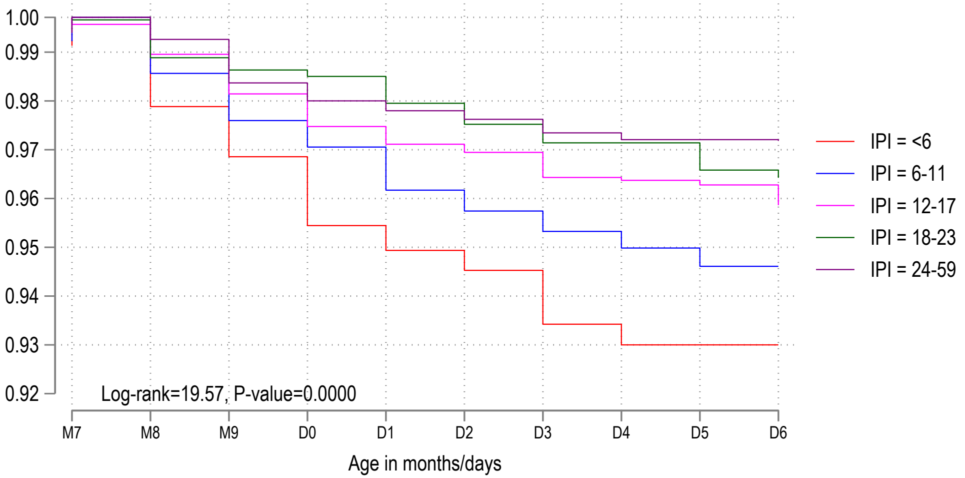

Number at risk

IPI = <6	316	310	301	295	290	289	288	284	283	283
IPI = 6-11	630	619	604	595	592	586	584	581	579	577
IPI = 12-17	1081	1073	1051	1028	1021	1017	1015	1010	1009	1008
IPI = 18-23	1283	1258	1211	1190	1189	1182	1177	1172	1172	1166
IPI = 24-59	1971	1916	1852	1810	1803	1799	1796	1791	1788	1787

E-Figure 1.37: Mozambique (2011)

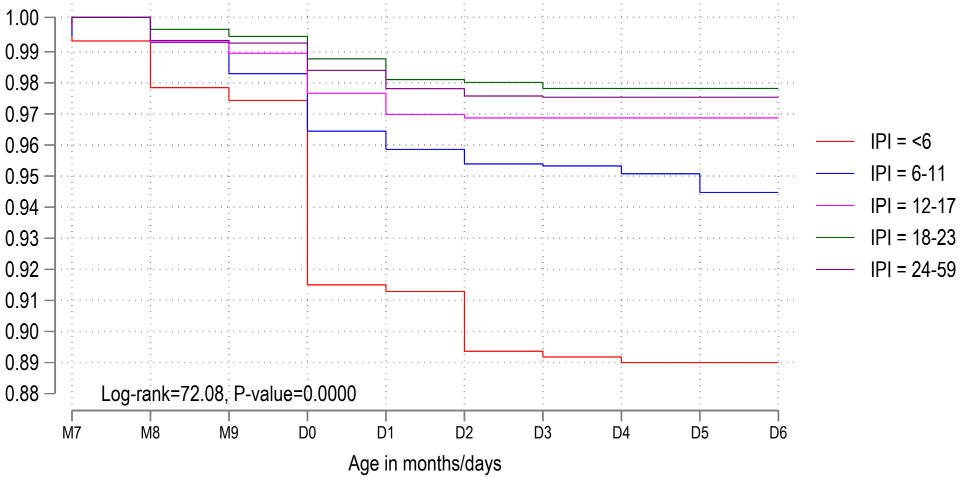

Number at risk

IPI = <6	315	310	299	297	279	278	272	271	271	271
IPI = 6-11	545	531	523	515	505	502	500	499	498	495
IPI = 12-17	1191	1159	1134	1110	1096	1088	1087	1087	1087	1087
IPI = 18-23	1378	1336	1294	1273	1264	1255	1254	1252	1252	1250
IPI = 24-59	2021	1946	1856	1822	1806	1795	1791	1790	1790	1790

E-Figure 1.38: Namibia (2006/7)

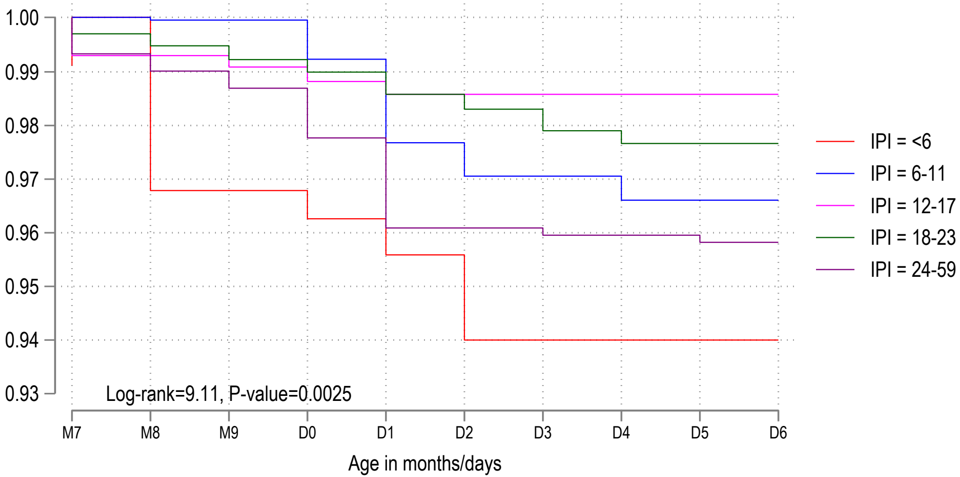

Number at risk

IPI = <6	132	127	123	122	122	121	119	119	119	119
IPI = 6-11	189	187	182	182	181	178	177	177	176	176
IPI = 12-17	335	328	323	318	318	317	317	317	317	317
IPI = 18-23	362	351	345	340	340	338	337	336	335	335
IPI = 24-59	735	702	674	664	657	646	646	645	645	644

E-Figure 1.39: Niger (2006)

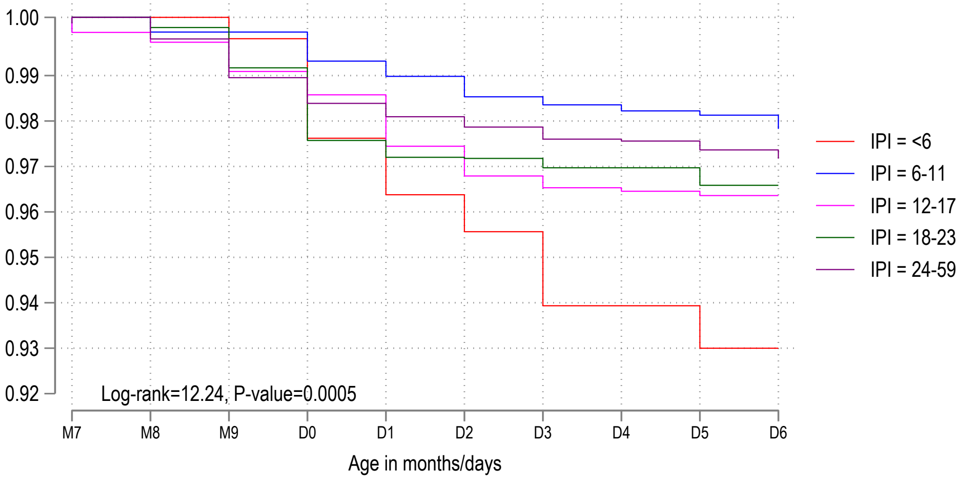

Number at risk

	M7	M8	M9	D0	D1	D2	D3	D4	D5	D6
IPI = <6	268	258	251	245	240	237	235	231	231	228
IPI = 6-11	518	512	498	493	490	488	486	485	485	484
IPI = 12-17	1164	1133	1109	1086	1080	1068	1061	1058	1057	1056
IPI = 18-23	1033	1005	967	935	920	916	916	914	914	911
IPI = 24-59	1519	1442	1367	1323	1315	1311	1308	1305	1304	1302

E-Figure 1.40: Niger (2012)

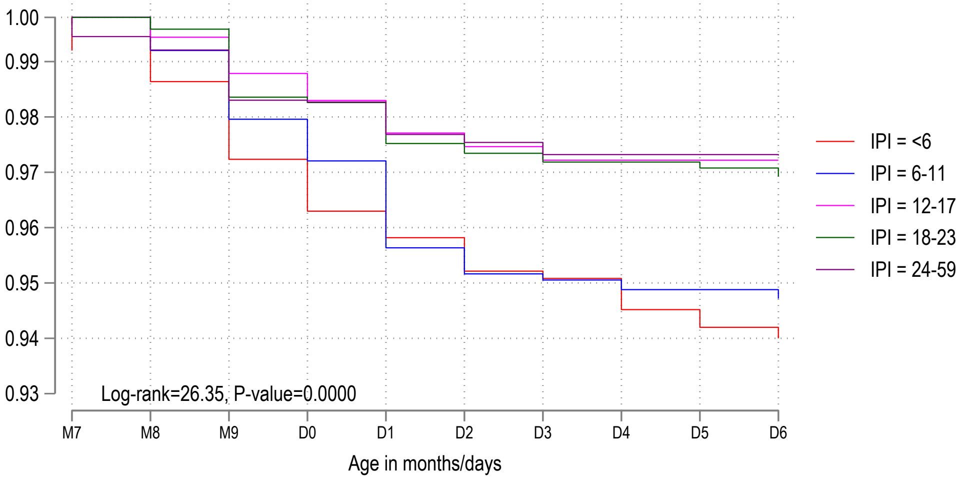

Number at risk

IPI = <6	555	543	532	523	518	515	512	512	509	507
IPI = 6-11	850	837	814	804	797	785	781	780	778	778
IPI = 12-17	1876	1845	1801	1784	1775	1764	1760	1755	1755	1755
IPI = 18-23	1666	1604	1538	1503	1502	1491	1488	1486	1486	1484
IPI = 24-59	1904	1788	1684	1650	1649	1640	1637	1633	1633	1633

E-Figure 1.41: Nigeria (2008)

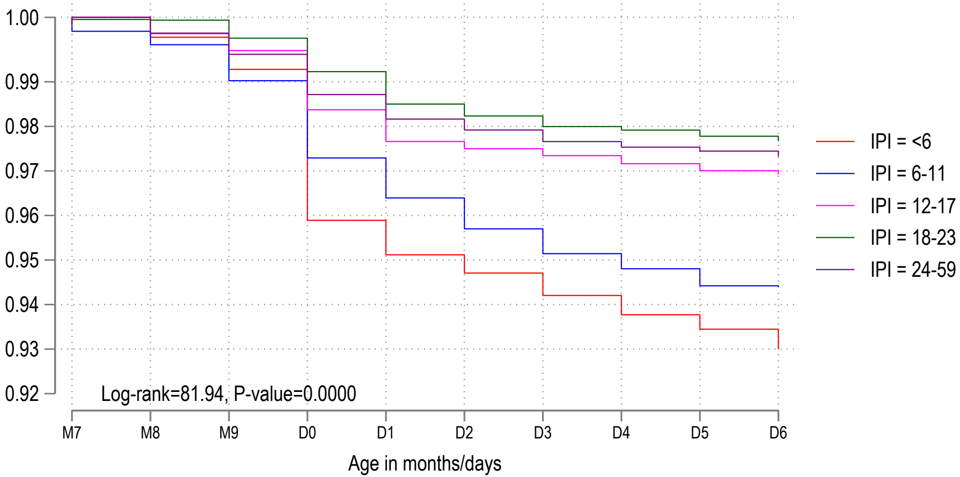

Number at risk

IPI = <6	1243	1219	1187	1172	1132	1123	1118	1112	1107	1103
IPI = 6-11	2082	2043	2007	1981	1946	1928	1914	1903	1896	1888
IPI = 12-17	3889	3817	3739	3676	3627	3601	3595	3589	3582	3576
IPI = 18-23	3175	3086	2996	2951	2929	2907	2899	2892	2890	2886
IPI = 24-59	4386	4200	4035	3954	3918	3896	3887	3876	3871	3868

E-Figure 1.42: Nigeria (2013)

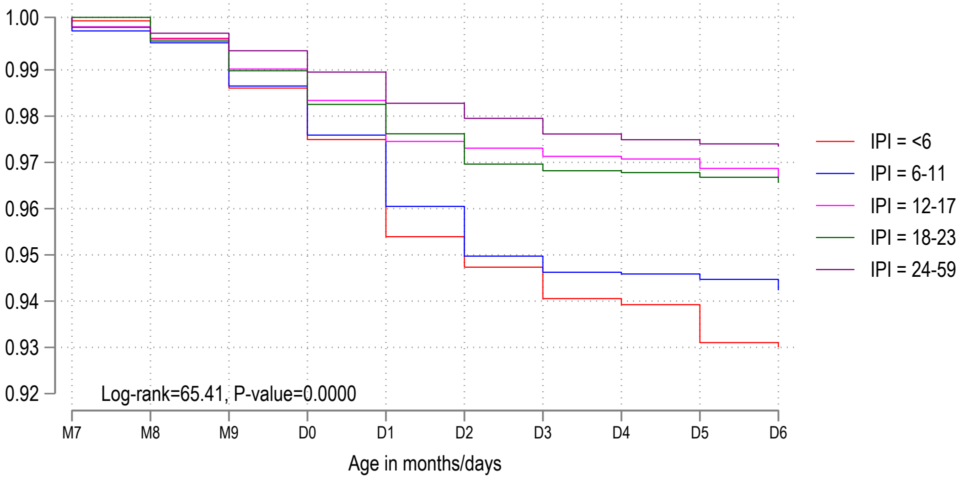

Number at risk

IPI = <6	1089	1054	1022	998	986	965	959	952	950	942
IPI = 6-11	2042	1999	1939	1906	1885	1855	1835	1828	1827	1825
IPI = 12-17	4315	4197	4062	4005	3977	3942	3936	3928	3926	3918
IPI = 18-23	3169	3058	2925	2871	2850	2832	2813	2808	2807	2804
IPI = 24-59	4781	4532	4246	4164	4145	4117	4103	4089	4084	4080

E-Figure 1.43: Nigeria (2018)

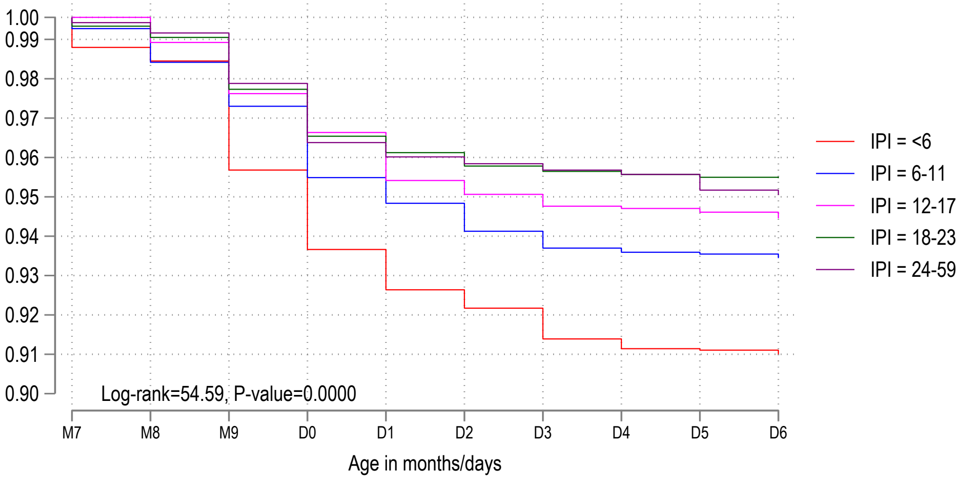

Number at risk

IPI = <6	1527	1490	1465	1406	1376	1361	1354	1343	1339	1338
IPI = 6-11	2532	2483	2414	2362	2318	2303	2286	2275	2273	2271
IPI = 12-17	4819	4734	4636	4533	4488	4431	4415	4401	4398	4394
IPI = 18-23	4120	3987	3849	3755	3710	3694	3681	3676	3673	3670
IPI = 24-59	5784	5562	5334	5163	5084	5065	5056	5047	5041	5019

E-Figure 1.44: Rwanda (2000)

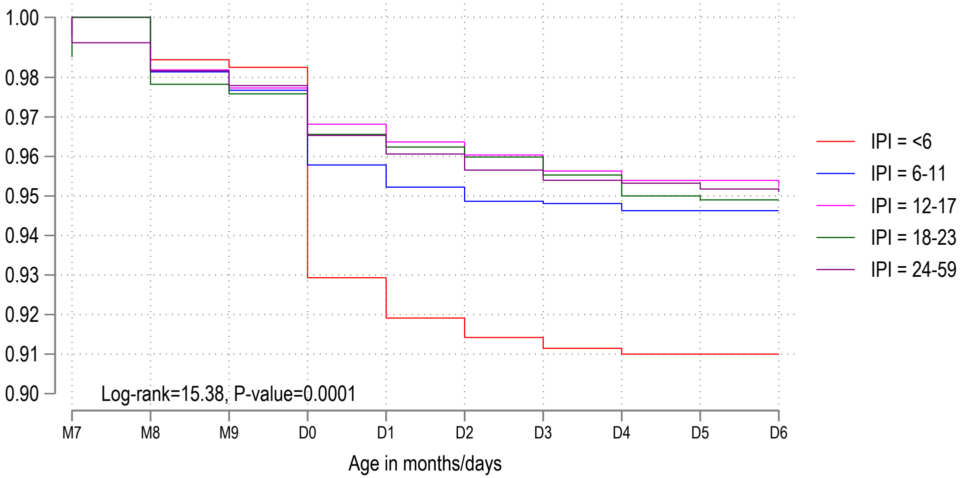

Number at risk

IPI = <6	560	551	526	524	496	490	488	486	485	485
IPI = 6-11	628	613	589	583	572	569	567	566	565	565
IPI = 12-17	1016	996	974	962	953	949	945	941	939	939
IPI = 18-23	834	799	770	759	751	749	747	744	739	739
IPI = 24-59	1365	1312	1248	1229	1213	1207	1202	1199	1198	1196

E-Figure 1.45: Rwanda (2005)

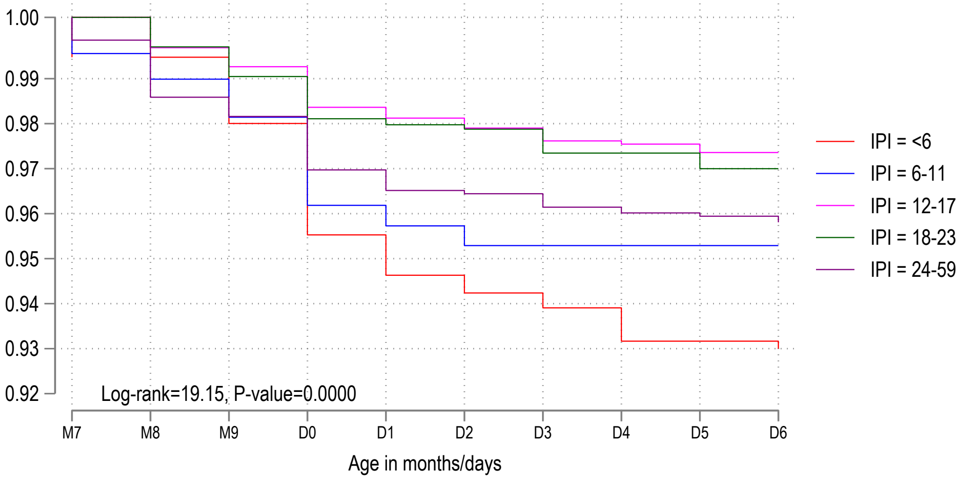

Number at risk

IPI = <6	521	507	493	478	465	461	459	457	454	454
IPI = 6-11	636	615	599	587	575	573	570	570	570	569
IPI = 12-17	1203	1177	1145	1131	1120	1118	1115	1112	1111	1109
IPI = 18-23	1006	979	952	942	933	932	931	926	926	922
IPI = 24-59	1070	1033	974	964	952	948	947	944	943	942

E-Figure 1.46: Rwanda (2010/11)

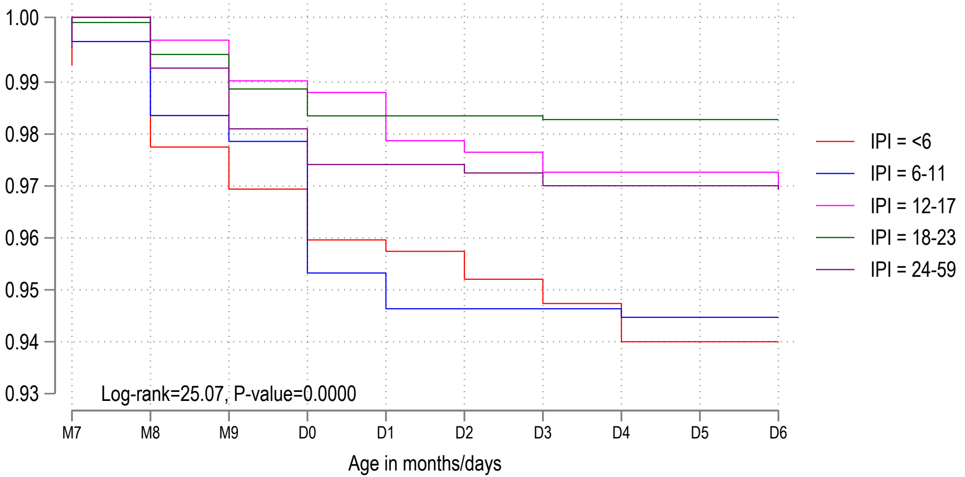

Number at risk

IPI = <6	444	437	423	415	410	409	407	405	402	402
IPI = 6-11	630	619	604	598	583	578	578	578	577	577
IPI = 12-17	1015	1001	984	970	967	958	956	952	952	952
IPI = 18-23	1024	1002	973	966	961	961	961	960	960	960
IPI = 24-59	1388	1335	1281	1257	1248	1248	1246	1243	1243	1243

E-Figure 1.47: Rwanda (2014/15)

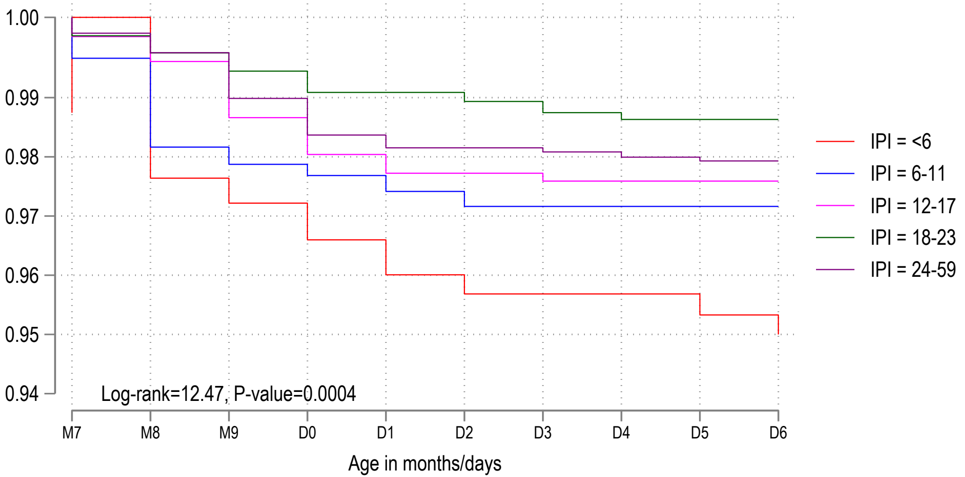

Number at risk

IPI = <6	352	338	326	321	319	317	316	316	316	315
IPI = 6-11	442	433	416	415	414	413	412	412	412	412
IPI = 12-17	626	612	603	597	593	591	591	590	590	590
IPI = 18-23	668	657	640	634	632	632	631	630	629	629
IPI = 24-59	1352	1302	1252	1232	1224	1221	1221	1221	1220	1219

E-Figure 1.48: Rwanda (2019/20)

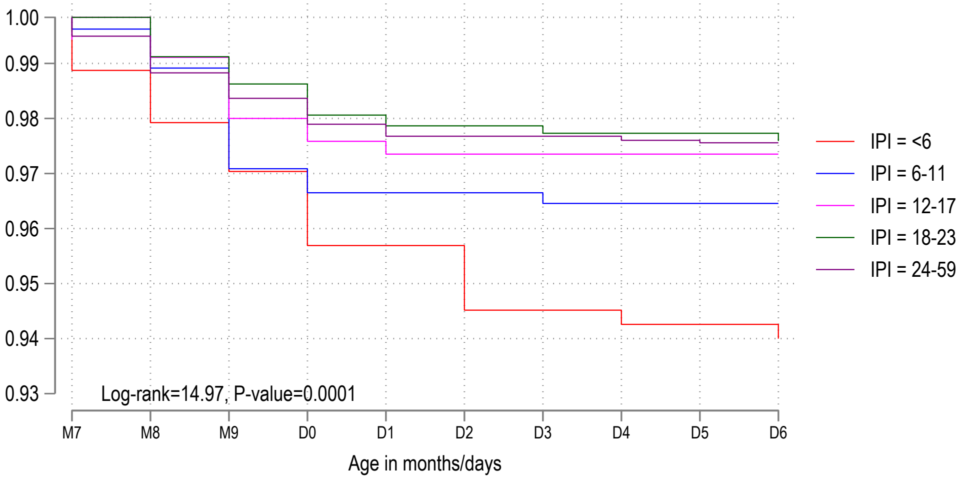

Number at risk

IPI = <6	373	361	350	346	342	342	337	337	336	336
IPI = 6-11	487	474	458	447	445	445	445	444	444	444
IPI = 12-17	709	691	680	669	666	664	664	664	664	664
IPI = 18-23	653	642	629	622	618	617	617	616	616	616
IPI = 24-59	1423	1377	1333	1318	1312	1309	1309	1309	1308	1307

E-Figure 1.49: Senegal (2005)

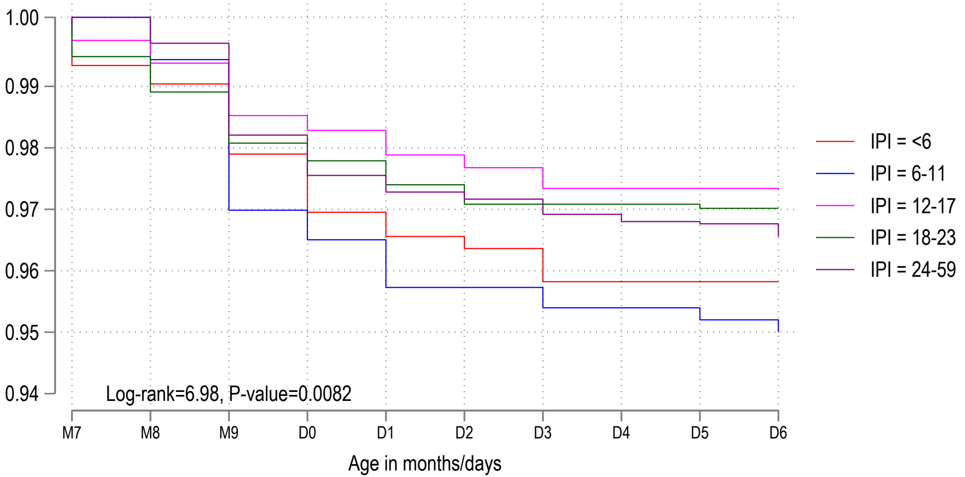

Number at risk

IPI = <6	384	371	365	358	355	353	353	351	351	351
IPI = 6-11	586	572	557	539	537	532	532	530	530	529
IPI = 12-17	1218	1195	1170	1141	1138	1133	1131	1127	1127	1127
IPI = 18-23	1043	1009	980	961	959	955	952	952	952	951
IPI = 24-59	1427	1369	1311	1275	1266	1263	1261	1258	1256	1256

E-Figure 1.50: Senegal (2010/11)

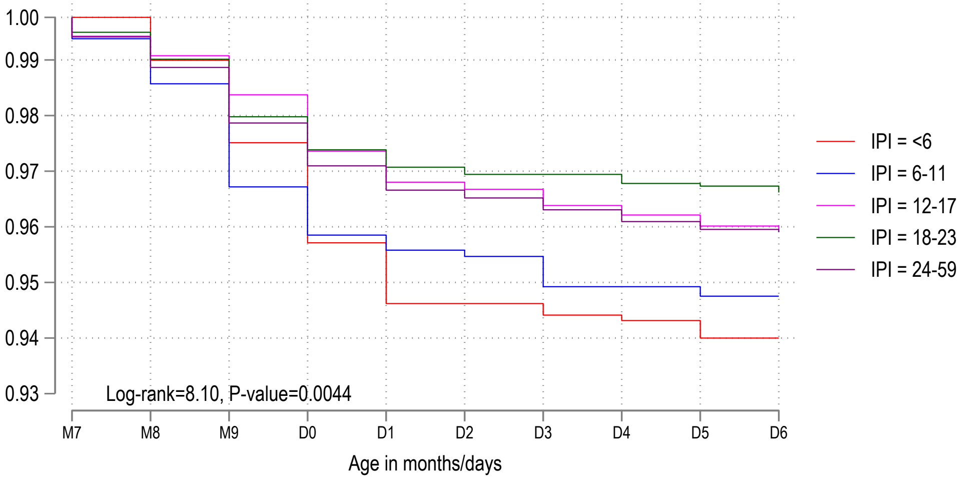

Number at risk

IPI = <6	494	487	477	462	453	448	448	447	447	445
IPI = 6-11	641	626	613	599	593	591	591	587	587	586
IPI = 12-17	1412	1385	1354	1333	1319	1312	1310	1306	1304	1301
IPI = 18-23	1350	1306	1261	1225	1217	1214	1212	1212	1210	1209
IPI = 24-59	2063	1994	1924	1872	1858	1849	1847	1843	1839	1836

E-Figure 1.51: Senegal (2015)

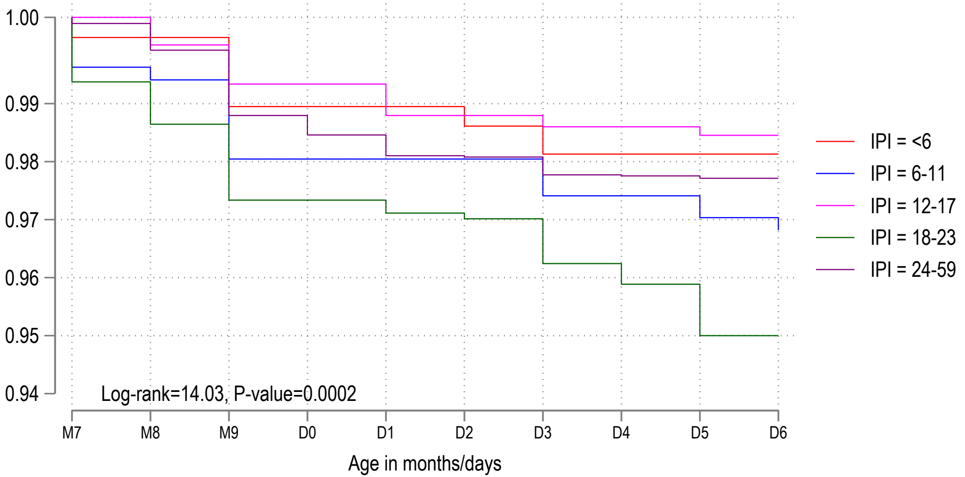

Number at risk

	M7	M8	M9	D0	D1	D2	D3	D4	D5	D6
IPI = <6	196	194	193	187	187	187	187	186	186	186
IPI = 6-11	318	308	303	296	296	296	296	294	294	293
IPI = 12-17	580	566	546	540	540	537	537	536	536	535
IPI = 18-23	687	670	646	635	635	634	633	628	626	620
IPI = 24-59	934	900	872	842	840	837	836	834	834	833

E-Figure 1.52: Senegal (2016)

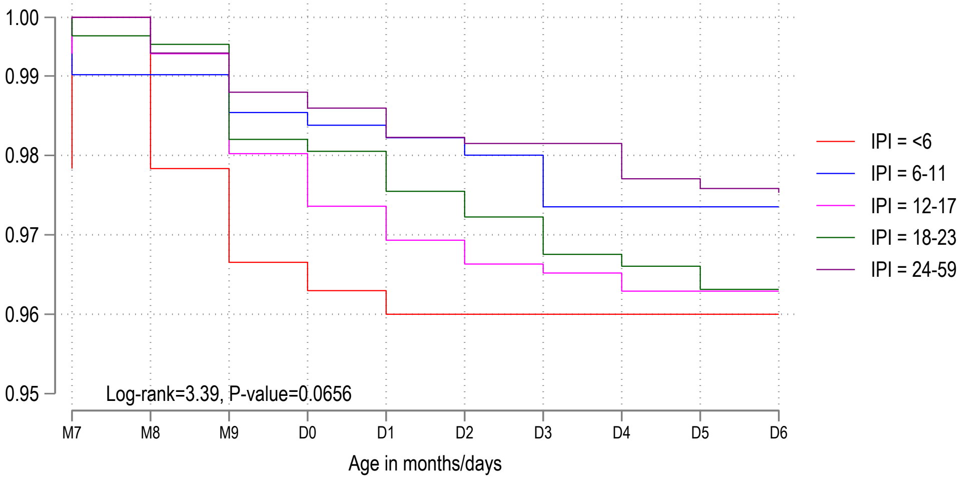

Number at risk

IPI = <6	216	198	196	189	189	188	188	188	188	188
IPI = 6-11	269	260	257	252	251	251	250	248	248	248
IPI = 12-17	608	589	580	570	566	563	562	561	560	560
IPI = 18-23	596	585	567	546	545	543	541	538	537	536
IPI = 24-59	858	826	780	751	749	746	746	746	742	741

E-Figure 1.53: Senegal (2018)

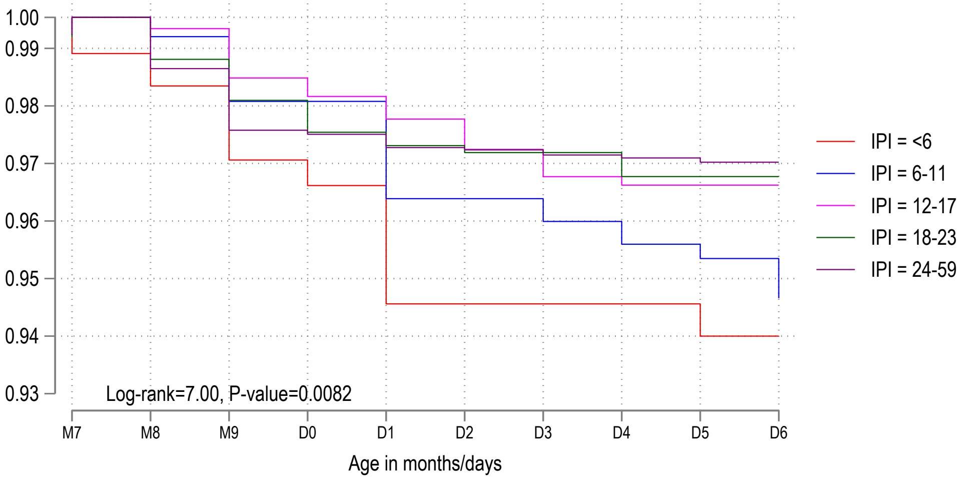

Number at risk

IPI = <6	182	177	175	171	170	167	167	167	167	166
IPI = 6-11	264	251	248	244	244	240	240	239	238	237
IPI = 12-17	596	574	558	544	542	540	537	534	534	534
IPI = 18-23	601	580	563	556	553	552	551	551	549	549
IPI = 24-59	927	884	843	812	812	810	810	809	808	807

E-Figure 1.54: Senegal (2019)

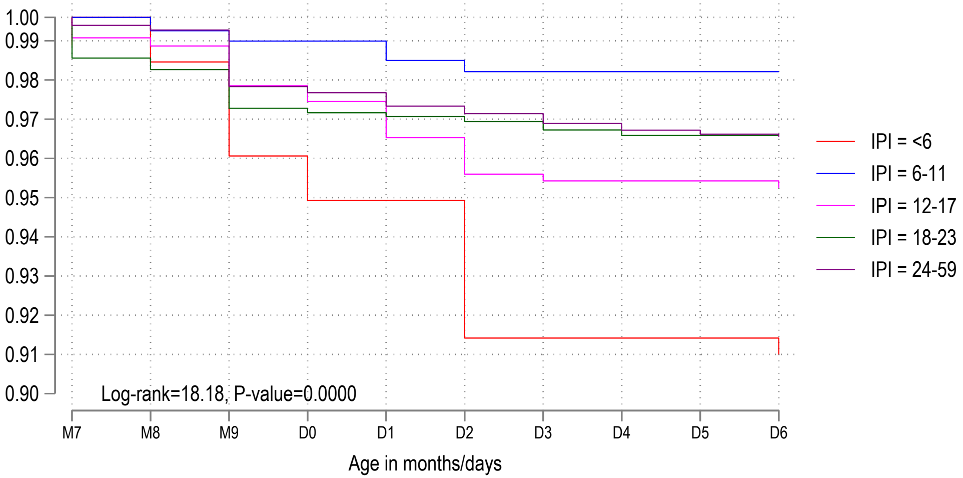

Number at risk

	M7	M8	M9	D0	D1	D2	D3	D4	D5	D6
IPI = <6	184	179	175	170	168	168	162	162	162	162
IPI = 6-11	253	248	240	232	232	231	230	230	230	230
IPI = 12-17	515	504	495	481	479	475	470	469	469	469
IPI = 18-23	593	579	566	549	548	547	547	545	545	545
IPI = 24-59	843	814	778	741	740	737	736	734	732	732

E-Figure 1.55: Sierra Leone (2008)

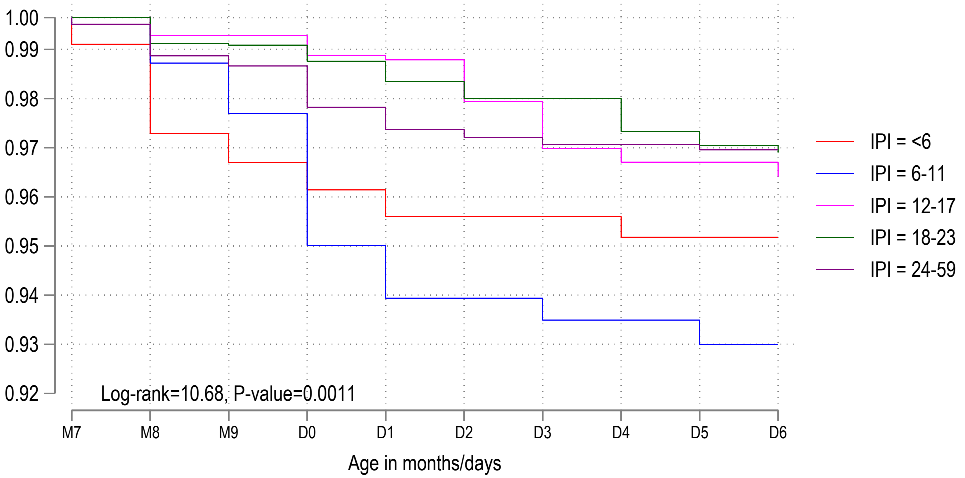

Number at risk

IPI = <6	170	168	164	163	162	161	161	161	160	160
IPI = 6-11	264	260	253	247	240	237	237	236	236	235
IPI = 12-17	567	560	541	541	539	538	534	528	527	527
IPI = 18-23	466	455	437	429	428	426	425	425	422	420
IPI = 24-59	880	845	795	787	780	776	775	774	774	773

E-Figure 1.56: Sierra Leone (2013)

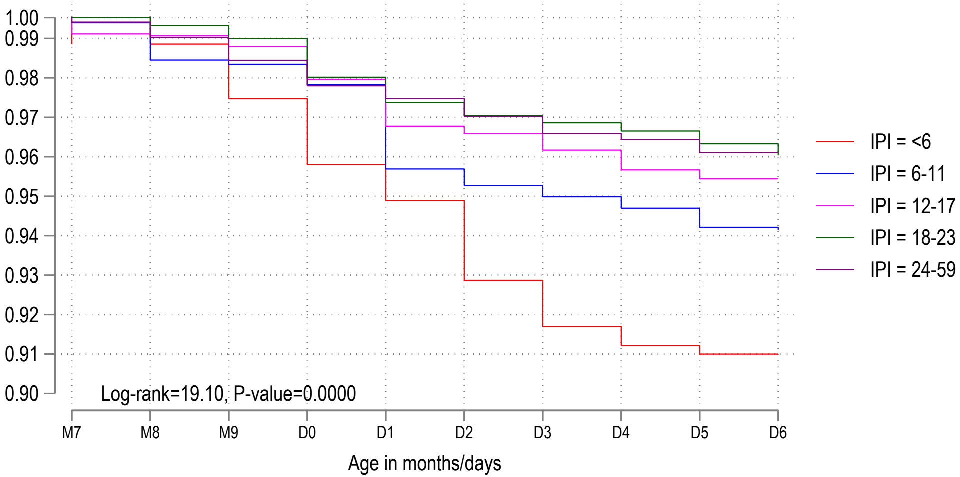

Number at risk

IPI = <6	280	273	266	261	257	254	249	246	245	244
IPI = 6-11	545	537	528	522	520	508	506	505	503	501
IPI = 12-17	1205	1190	1174	1157	1148	1134	1131	1127	1121	1118
IPI = 18-23	1211	1184	1144	1124	1113	1106	1102	1100	1098	1094
IPI = 24-59	1998	1925	1842	1774	1762	1756	1748	1740	1738	1732

E-Figure 1.57: Tanzania (2004/5)

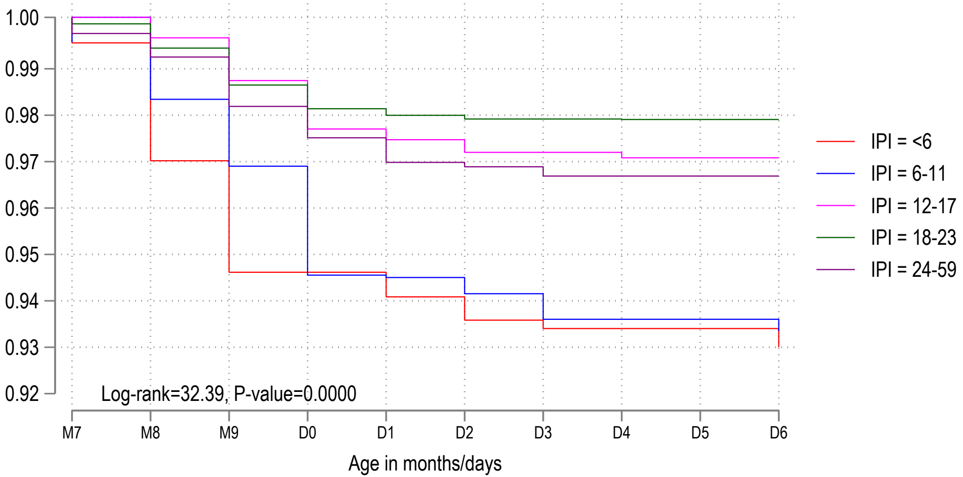

Number at risk

IPI = <6	329	317	301	291	291	290	288	287	287	287
IPI = 6-11	502	489	475	465	453	453	451	449	449	449
IPI = 12-17	1136	1113	1096	1082	1070	1068	1065	1065	1064	1064
IPI = 18-23	1126	1102	1061	1043	1037	1036	1035	1035	1035	1035
IPI = 24-59	1477	1429	1363	1324	1315	1308	1307	1304	1304	1304

E-Figure 1.58: Tanzania (2010)

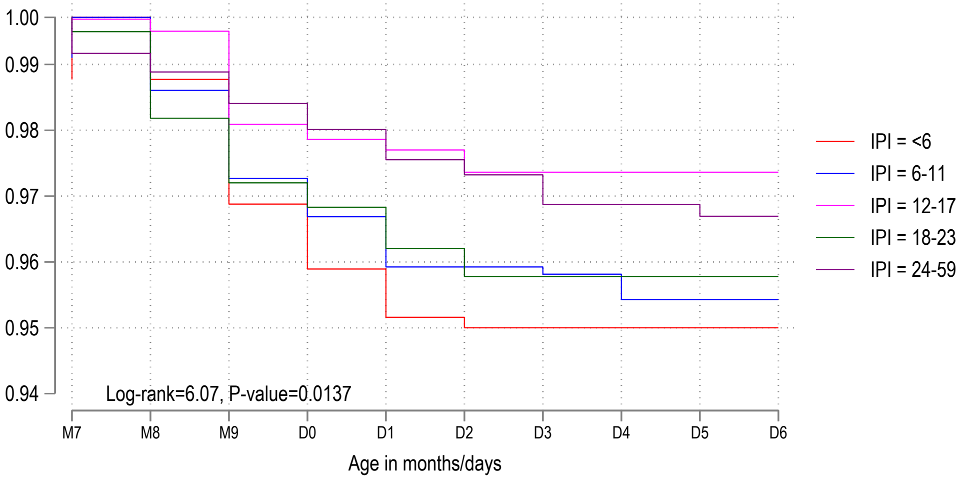

Number at risk

IPI = <6	224	215	210	204	201	200	200	200	200	200
IPI = 6-11	405	394	387	378	375	372	372	372	370	370
IPI = 12-17	839	820	794	776	775	773	771	771	771	771
IPI = 18-23	847	821	782	766	763	758	755	755	755	755
IPI = 24-59	963	926	879	857	854	850	848	844	844	843

E-Figure 1.59: Tanzania (2015/16)

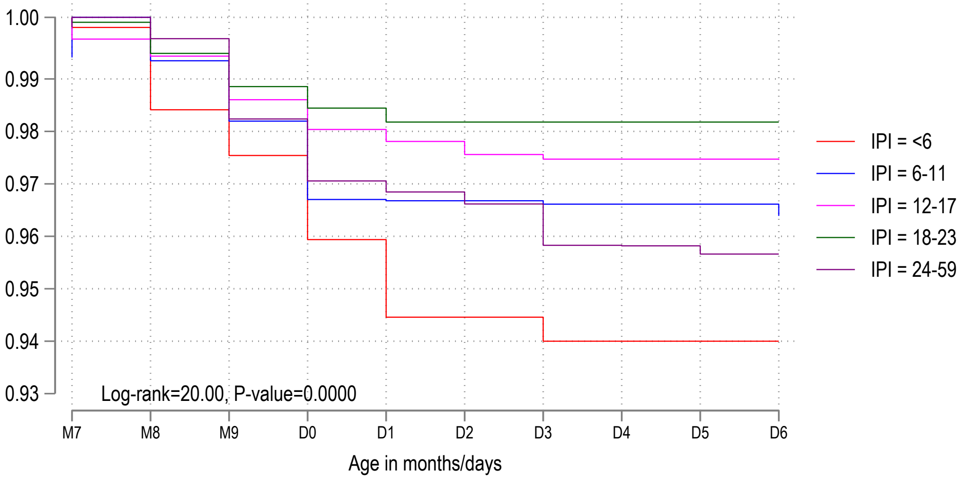

Number at risk

	M7	M8	M9	D0	D1	D2	D3	D4	D5	D6
IPI = <6	406	398	384	376	369	364	364	362	362	362
IPI = 6-11	586	576	563	554	546	546	546	545	545	545
IPI = 12-17	1117	1096	1073	1056	1050	1047	1044	1043	1043	1043
IPI = 18-23	991	966	939	927	923	920	920	920	920	920
IPI = 24-59	1507	1459	1385	1351	1335	1332	1329	1318	1318	1316

E-Figure 1.60: Uganda (2000/1)

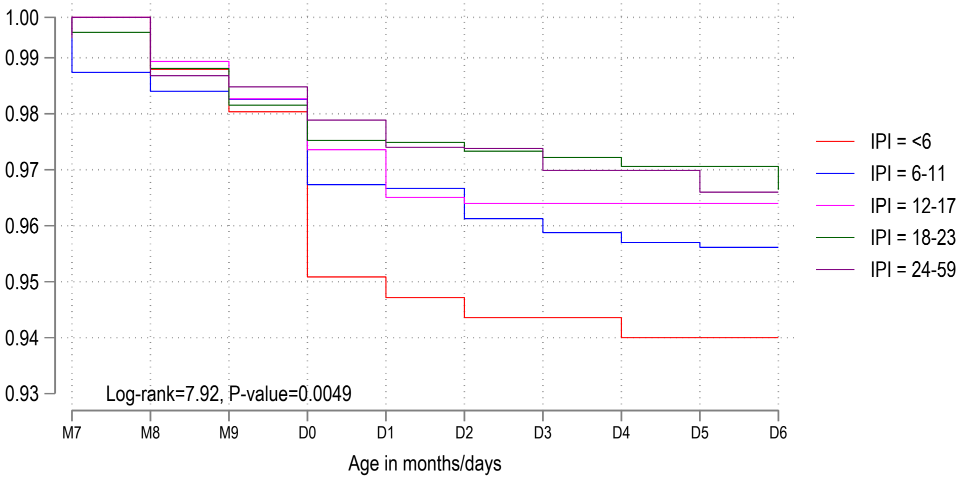

Number at risk

IPI = <6	443	438	430	425	412	411	409	409	407	407
IPI = 6-11	824	797	778	775	763	762	758	756	755	754
IPI = 12-17	1368	1329	1291	1272	1260	1249	1248	1248	1248	1248
IPI = 18-23	1045	1022	991	977	971	970	969	968	966	966
IPI = 24-59	1049	1018	973	962	956	951	951	947	947	943

E-Figure 1.61: Uganda (2006)

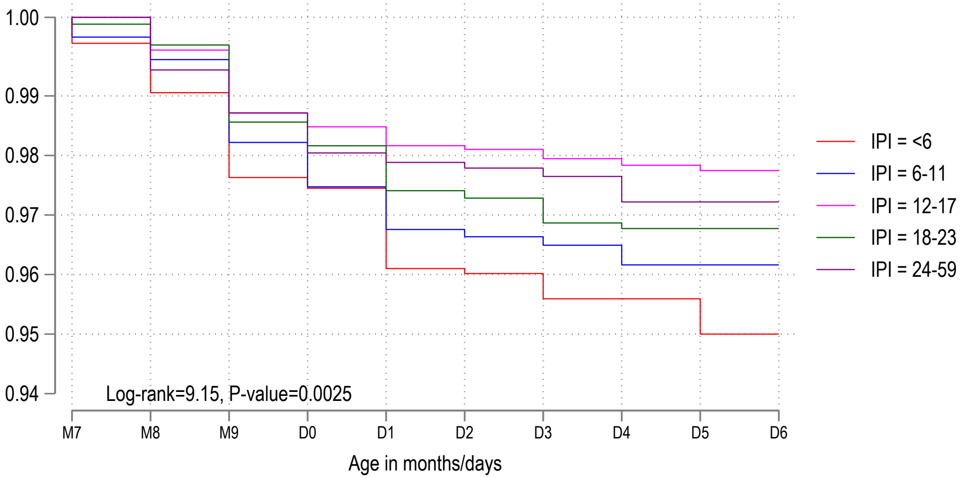

Number at risk

IPI = <6	502	489	479	467	467	460	460	458	458	453
IPI = 6-11	746	731	712	696	691	686	685	684	682	682
IPI = 12-17	1254	1232	1189	1163	1160	1157	1156	1154	1153	1151
IPI = 18-23	1032	999	962	938	934	927	925	921	921	921
IPI = 24-59	1005	963	927	904	898	897	896	895	891	889

E-Figure 1.62: Uganda (2011)

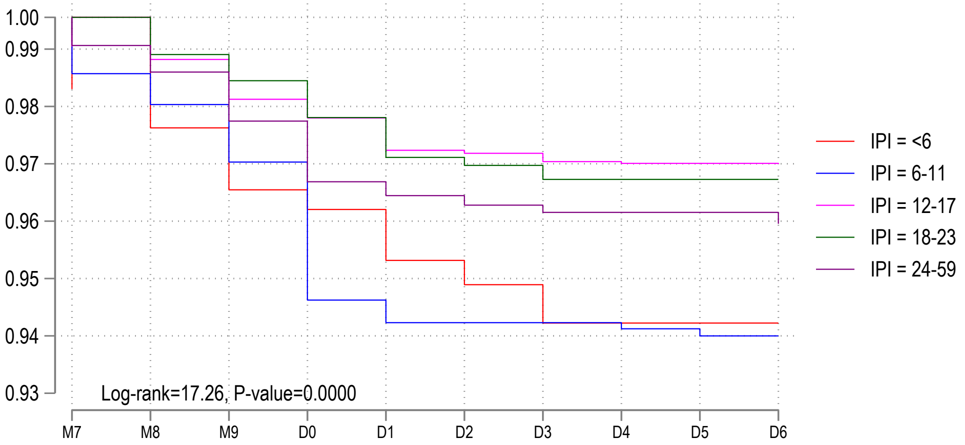

Number at risk

IPI = <6	478	462	439	432	430	426	424	422	422	422
IPI = 6-11	761	743	717	701	684	681	681	681	680	679
IPI = 12-17	1247	1219	1185	1168	1164	1157	1157	1155	1154	1154
IPI = 18-23	963	936	906	894	888	882	881	878	878	878
IPI = 24-59	1057	1005	960	935	925	923	921	920	920	920

E-Figure 1.63: Zambia (2007)

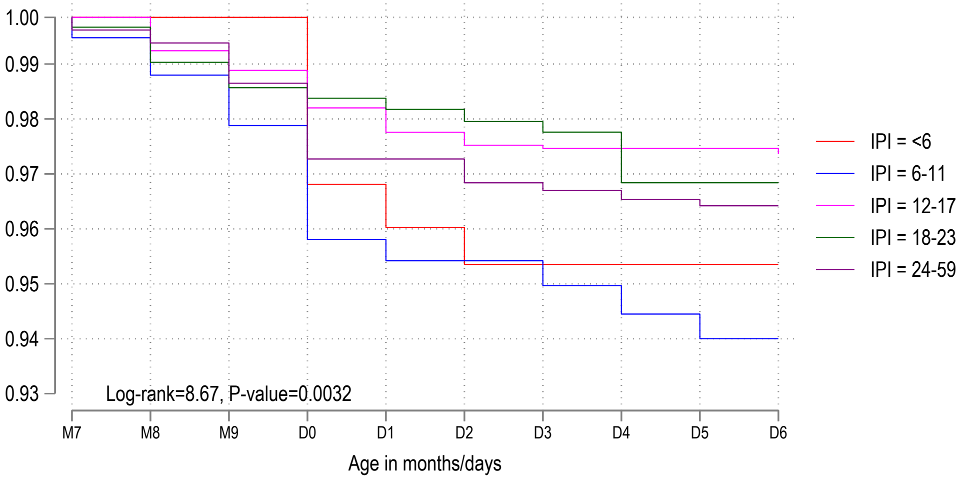

Number at risk

IPI = <6	209	207	200	196	190	188	187	187	187	187
IPI = 6-11	356	348	340	335	328	326	326	325	323	321
IPI = 12-17	698	679	659	650	646	643	641	641	641	641
IPI = 18-23	738	722	699	681	679	678	677	675	669	669
IPI = 24-59	1010	969	938	921	908	908	904	903	902	901

E-Figure 1.64: Zambia (2013/14)

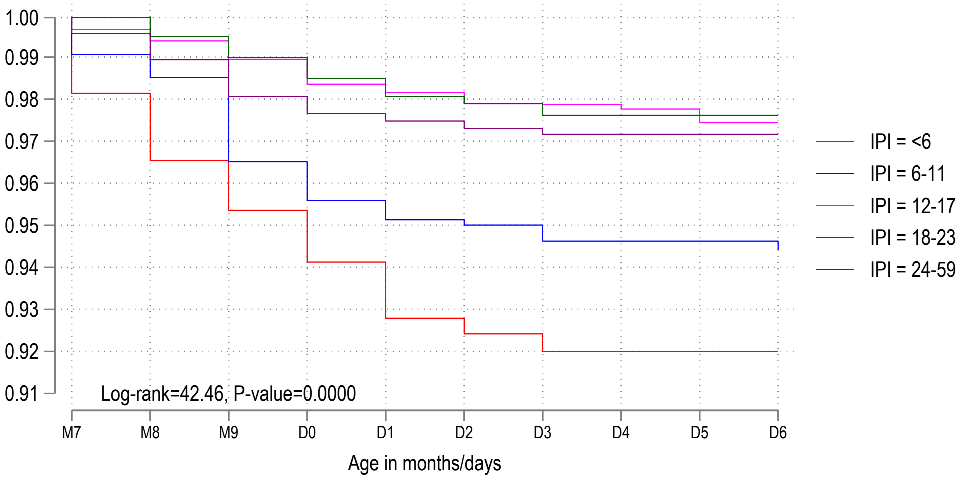

Number at risk

IPI = <6	305	294	286	279	275	271	270	269	269	269
IPI = 6-11	722	709	698	679	673	670	669	666	666	665
IPI = 12-17	1377	1360	1343	1324	1316	1313	1310	1309	1308	1303
IPI = 18-23	1506	1480	1444	1426	1418	1412	1410	1406	1406	1406
IPI = 24-59	2456	2375	2268	2213	2204	2200	2196	2193	2193	2192

E-Figure 1.65: Zambia (2018/19)

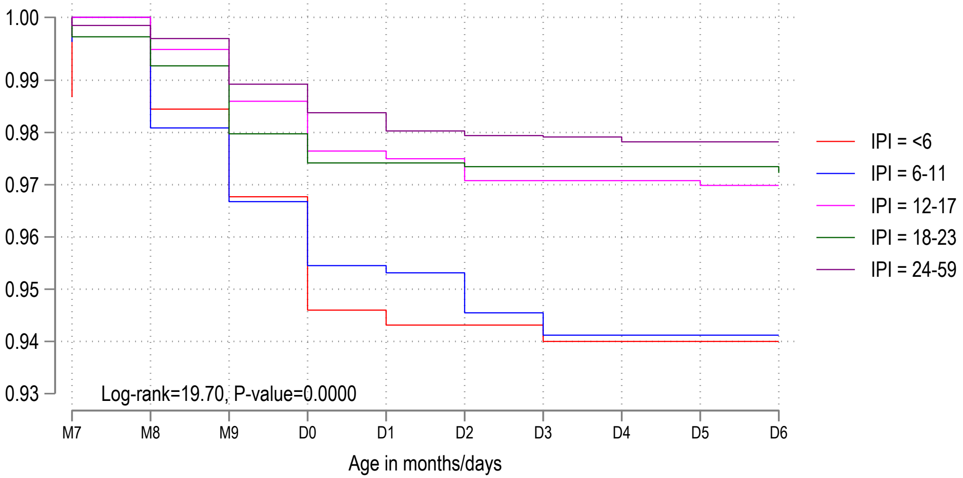

Number at risk

IPI = <6	251	246	241	235	229	229	229	228	228	228
IPI = 6-11	355	346	335	328	324	323	321	319	319	319
IPI = 12-17	801	784	768	749	742	740	737	737	737	737
IPI = 18-23	765	753	729	709	705	705	704	704	704	704
IPI = 24-59	1668	1600	1543	1494	1486	1480	1479	1479	1477	1476

E-Figure 1.66: Zimbabwe (2010/11)

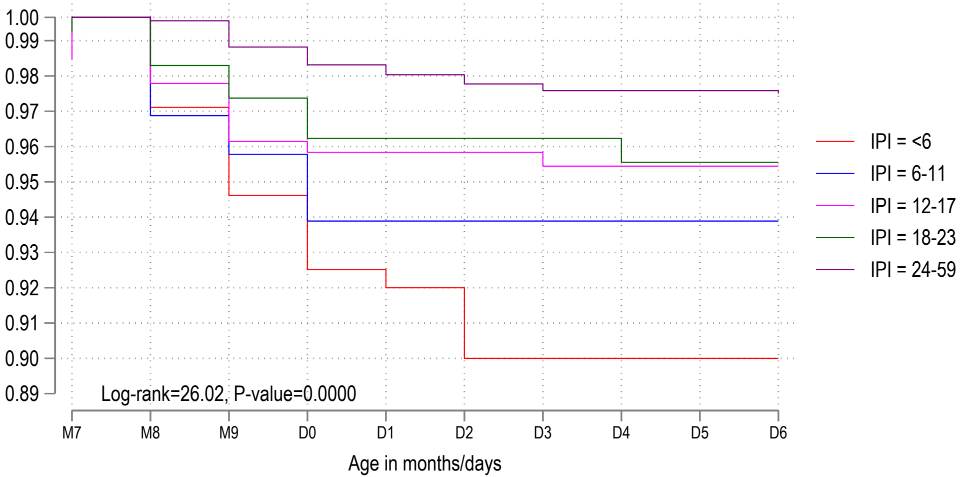

Number at risk

IPI = <6	179	179	171	165	161	161	157	157	157	157
IPI = 6-11	191	186	174	166	163	163	163	163	163	161
IPI = 12-17	275	265	252	244	243	243	243	242	242	242
IPI = 18-23	328	320	307	299	295	295	295	295	293	293
IPI = 24-59	1002	961	917	888	884	881	879	877	877	877

E-Figure 1.67: Zimbabwe (2015)

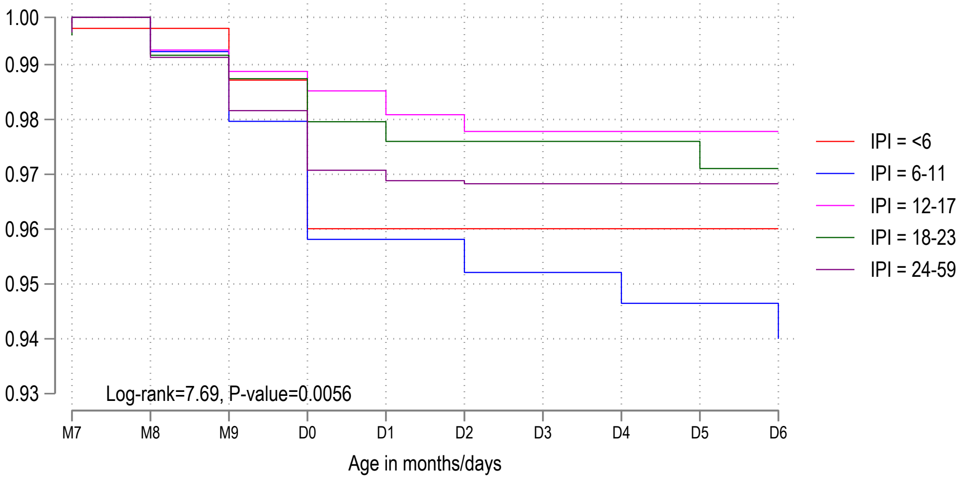

Number at risk

	M7	M8	M9	D0	D1	D2	D3	D4	D5	D6
IPI = <6	198	195	189	182	177	177	177	177	177	177
IPI = 6-11	258	251	247	243	237	237	236	236	234	234
IPI = 12-17	407	406	395	390	389	387	386	386	386	386
IPI = 18-23	435	427	419	411	408	406	406	406	406	404
IPI = 24-59	946	919	880	860	850	848	848	848	848	848

E-Figure 1.68: Egypt (2000)

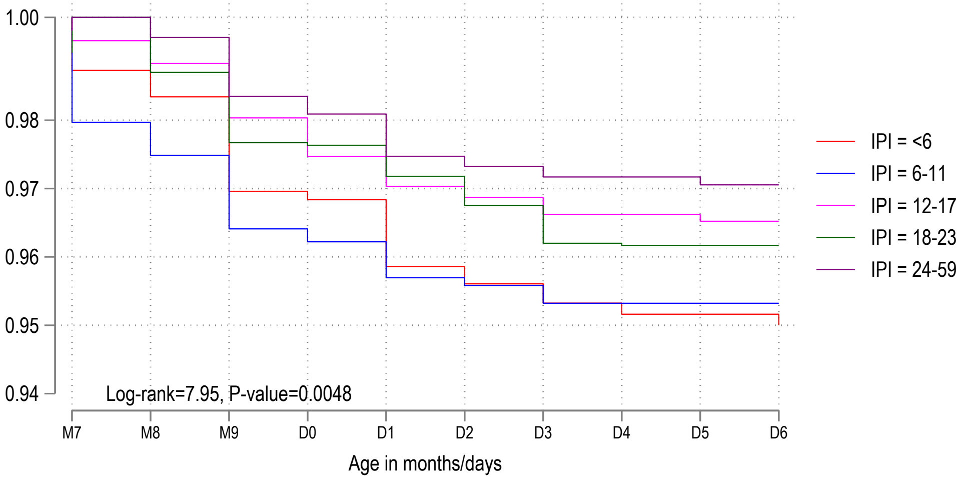

Number at risk

	M7	M8	M9	D0	D1	D2	D3	D4	D5	D6
IPI = <6	858	837	819	808	807	799	797	794	793	792
IPI = 6-11	785	768	744	735	734	730	729	727	727	727
IPI = 12-17	971	960	933	924	919	915	913	911	911	910
IPI = 18-23	748	720	697	690	690	687	684	680	679	679
IPI = 24-59	1030	988	940	932	930	924	922	921	921	918

E-Figure 1.69: Egypt (2003)

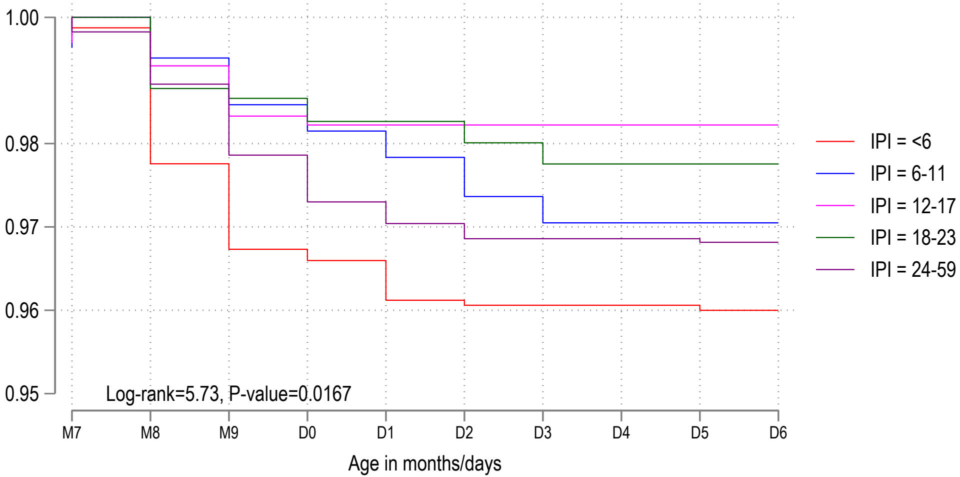

Number at risk

IPI = <6	422	418	406	402	401	399	399	399	399	399
IPI = 6-11	413	403	396	394	393	391	389	388	388	388
IPI = 12-17	492	477	465	462	462	462	462	462	462	462
IPI = 18-23	442	436	419	418	417	417	416	415	415	415
IPI = 24-59	649	618	584	579	575	574	573	573	573	572

E-Figure 1.70: Egypt (2005)

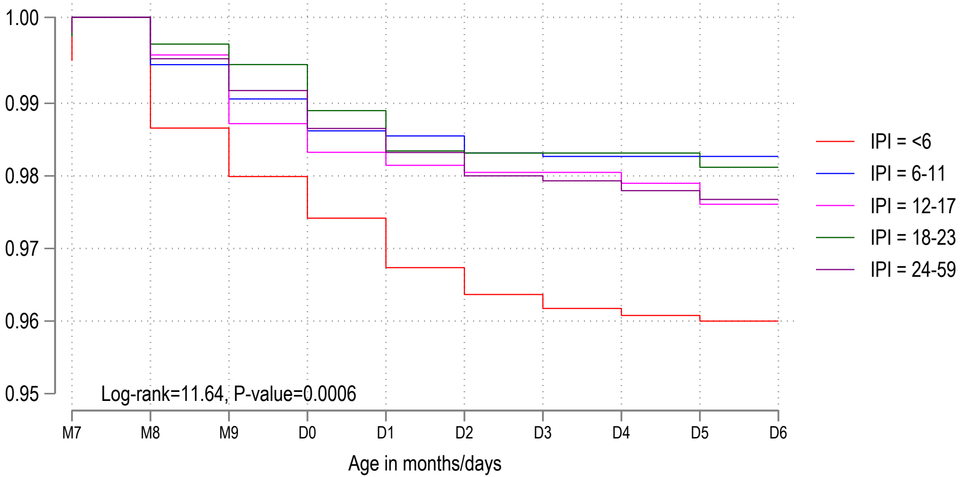

Number at risk

IPI = <6	939	920	901	895	890	883	880	878	877	875
IPI = 6-11	884	873	845	839	836	835	833	833	833	833
IPI = 12-17	1066	1047	1017	1007	1003	1001	1000	1000	999	994
IPI = 18-23	883	866	832	828	822	818	818	818	818	816
IPI = 24-59	1407	1360	1295	1283	1276	1272	1268	1267	1265	1264

E-Figure 1.71: Egypt (2008)

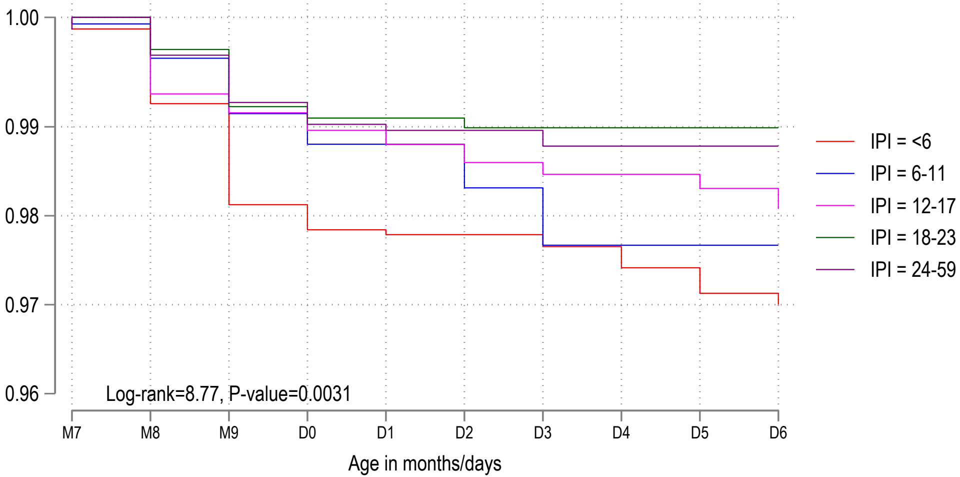

Number at risk

	M7	M8	M9	D0	D1	D2	D3	D4	D5	D6
IPI = <6	627	618	605	597	595	595	595	594	592	591
IPI = 6-11	540	535	516	510	509	509	506	503	503	503
IPI = 12-17	659	642	623	621	619	618	617	616	616	615
IPI = 18-23	643	631	610	604	604	604	603	603	603	603
IPI = 24-59	1074	1025	978	970	968	967	967	965	965	965

E-Figure 1.72: Egypt (2014)

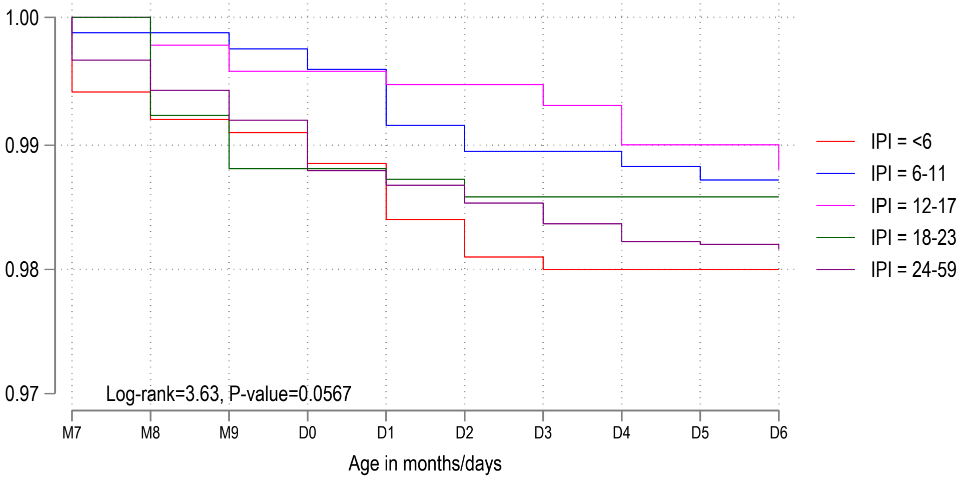

Number at risk

IPI = <6	988	963	947	944	942	938	935	934	934	934
IPI = 6-11	1086	1060	1044	1043	1041	1037	1034	1034	1033	1031
IPI = 12-17	1168	1130	1101	1098	1098	1097	1097	1095	1092	1092
IPI = 18-23	945	918	885	881	881	880	879	879	879	879
IPI = 24-59	1566	1491	1408	1405	1399	1397	1395	1393	1391	1390

E-Figure 1.73: Jordan (2002)

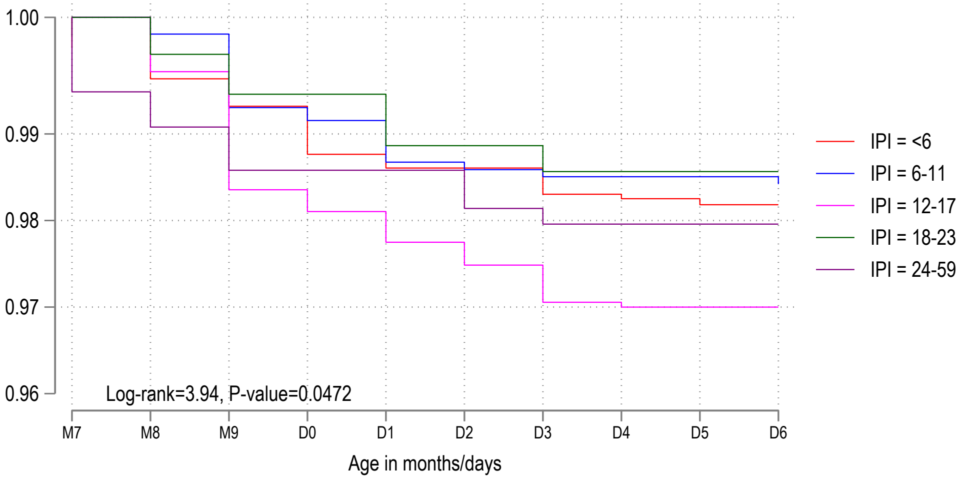

Number at risk

IPI = <6	707	692	674	665	661	660	660	658	658	657
IPI = 6-11	818	801	784	773	772	768	767	767	767	767
IPI = 12-17	652	639	623	604	603	601	599	596	596	596
IPI = 18-23	378	367	355	348	348	346	346	345	345	345
IPI = 24-59	583	558	530	524	524	524	522	521	521	521

E-Figure 1.74: Jordan (2009)

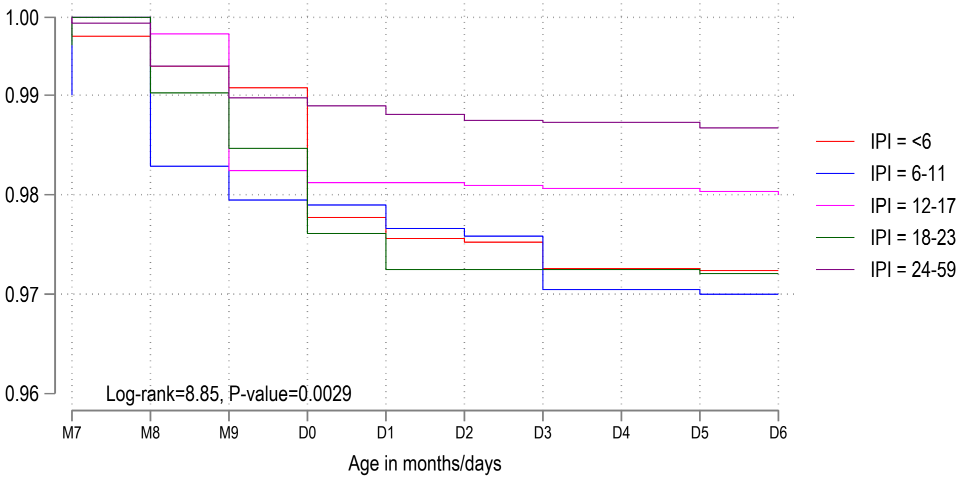

Number at risk

IPI = <6	1236	1211	1191	1188	1172	1170	1169	1166	1166	1166
IPI = 6-11	1226	1185	1147	1139	1138	1135	1134	1128	1128	1128
IPI = 12-17	1021	996	964	950	949	949	949	949	949	948
IPI = 18-23	604	582	548	545	541	539	539	539	539	538
IPI = 24-59	978	946	879	875	874	873	873	873	873	872

E-Figure 1.75: Jordan (2012)

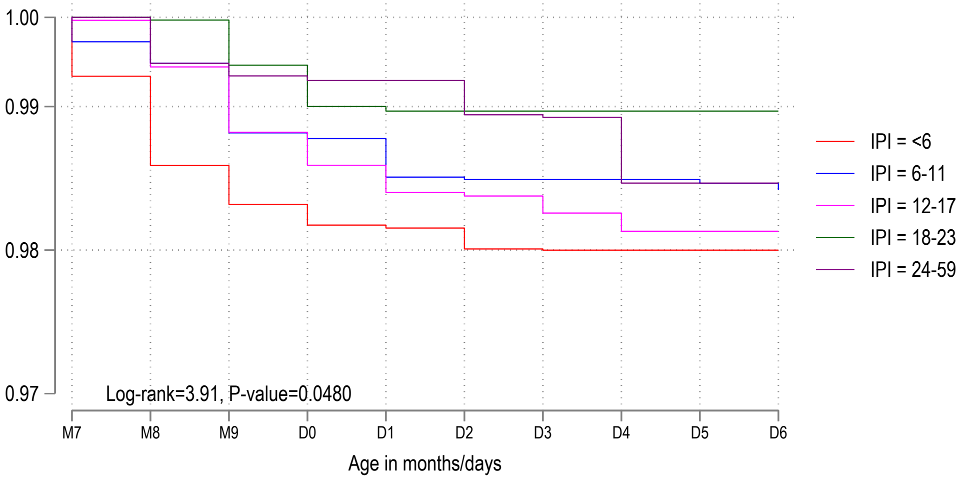

Number at risk

IPI = <6	1185	1167	1125	1116	1115	1115	1113	1113	1113	1112
IPI = 6-11	1325	1307	1263	1249	1249	1245	1245	1245	1245	1245
IPI = 12-17	961	938	900	895	893	892	891	890	889	889
IPI = 18-23	713	695	658	656	654	654	654	654	654	654
IPI = 24-59	1091	1056	996	993	993	993	990	990	986	986

E-Figure 1.76: Jordan (2017/18)

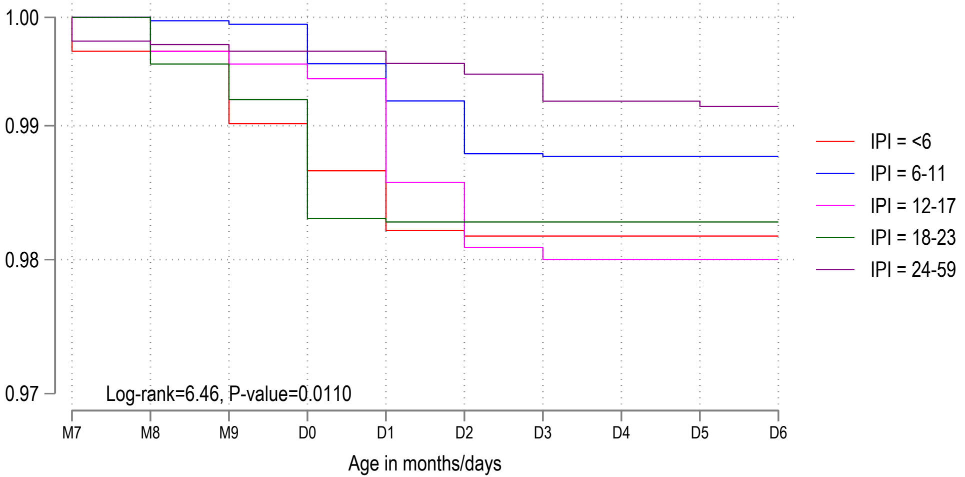

Number at risk

IPI = <6	999	966	955	937	934	929	929	929	929	929
IPI = 6-11	1030	1009	986	973	970	968	964	964	964	964
IPI = 12-17	780	764	750	738	737	732	728	727	727	727
IPI = 18-23	600	589	573	563	558	557	557	557	557	557
IPI = 24-59	1163	1115	1077	1041	1041	1040	1039	1037	1037	1037

E-Figure 1.77: Morocco (2003/4)

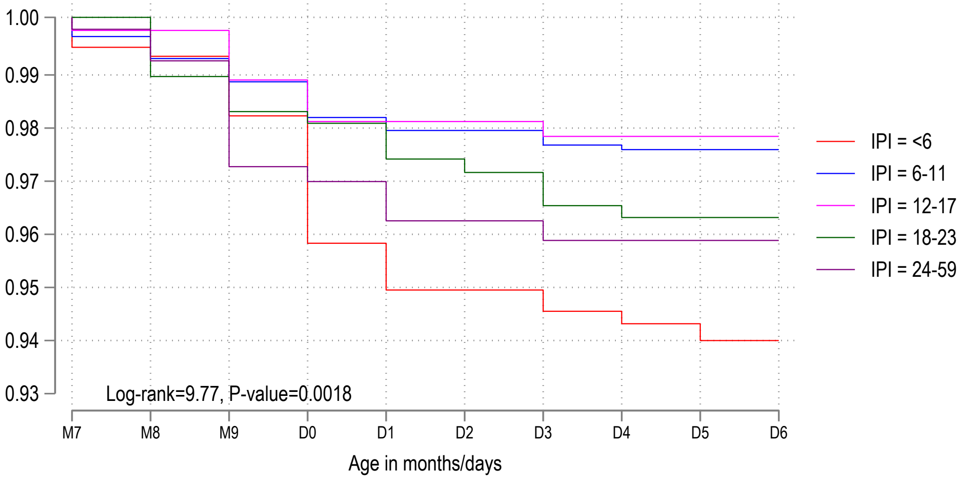

Number at risk

IPI = <6	354	350	343	335	327	324	324	323	322	321
IPI = 6-11	399	387	376	371	369	368	368	367	367	367
IPI = 12-17	440	435	424	411	408	408	408	406	406	406
IPI = 18-23	344	335	324	320	319	317	316	314	313	313
IPI = 24-59	946	916	878	840	838	831	831	828	828	828

E-Figure 1.78: Türkiye (2003/4)

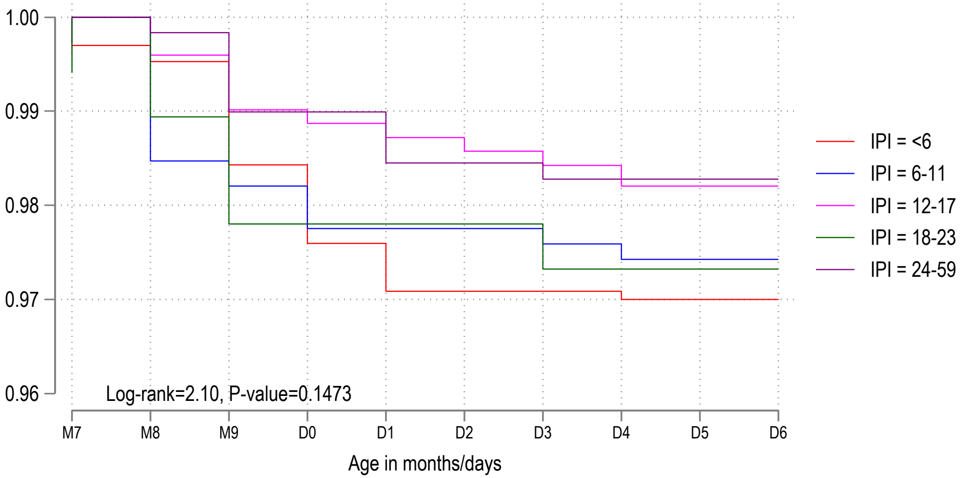

Number at risk

IPI = <6	390	378	373	368	364	363	363	363	362	362
IPI = 6-11	379	371	359	358	356	356	356	356	355	355
IPI = 12-17	410	399	394	390	390	389	388	388	387	387
IPI = 18-23	252	248	240	238	238	238	238	236	236	236
IPI = 24-59	381	367	355	344	344	342	342	342	342	342

E-Figure 1.79: Yemen (2013)

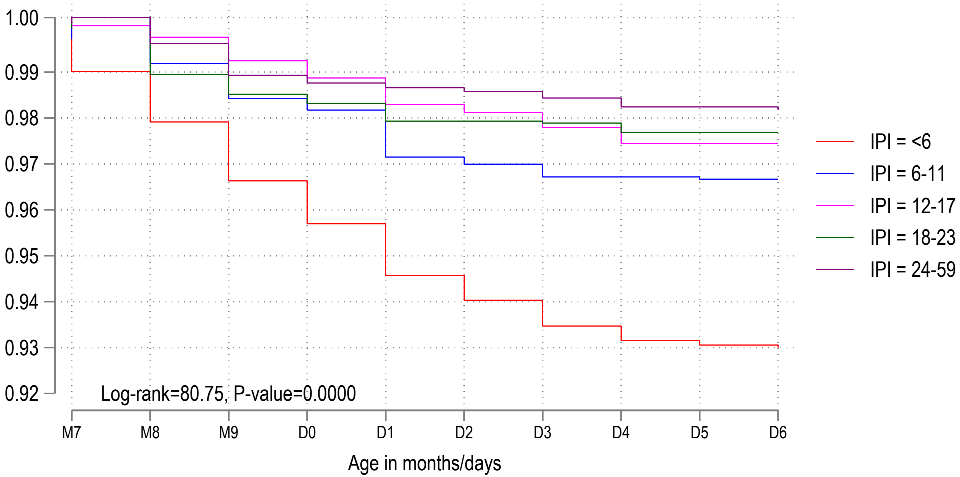

Number at risk

IPI = <6	1591	1554	1504	1466	1452	1434	1426	1418	1413	1411
IPI = 6-11	1749	1709	1651	1629	1625	1608	1605	1601	1601	1600
IPI = 12-17	1927	1892	1851	1823	1816	1805	1802	1796	1789	1789
IPI = 18-23	1192	1149	1098	1080	1078	1074	1074	1074	1071	1071
IPI = 24-59	2021	1951	1854	1793	1790	1788	1786	1784	1780	1780

E-Figure 1.80: Bangladesh (2004)

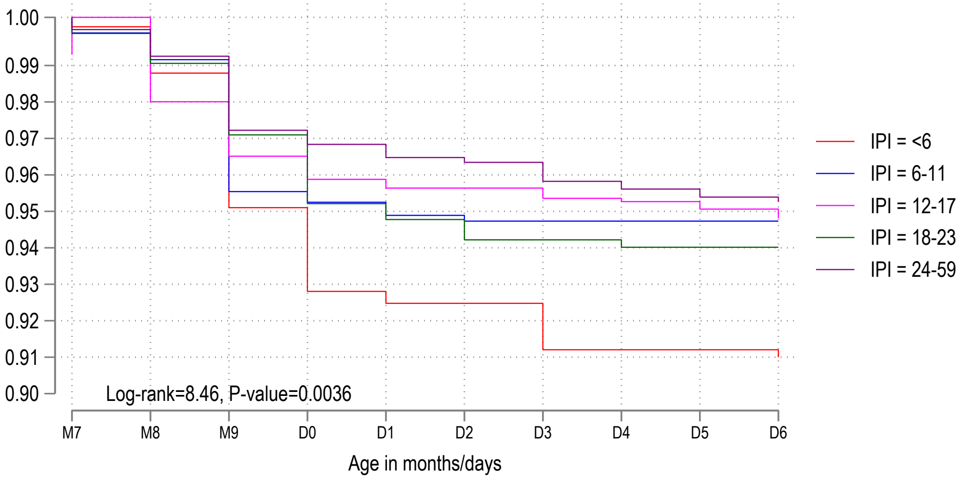

Number at risk

IPI = <6	360	353	342	324	316	315	315	311	311	311
IPI = 6-11	435	426	418	397	396	394	393	393	393	393
IPI = 12-17	654	642	627	613	609	607	607	605	605	603
IPI = 18-23	417	407	392	381	374	372	370	370	369	369
IPI = 24-59	943	919	875	842	839	836	834	830	828	826

E-Figure 1.81: Bangladesh (2007)

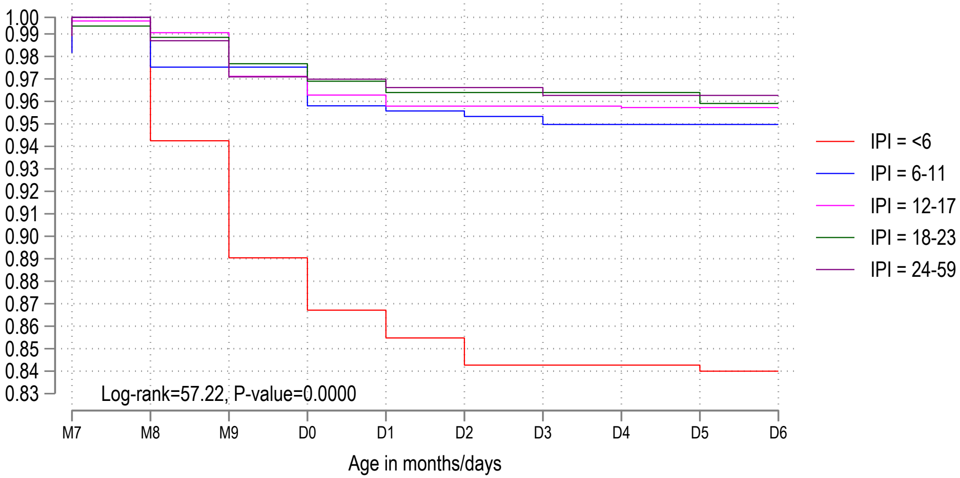

Number at risk

IPI = <6	247	241	227	213	208	205	202	202	202	201
IPI = 6-11	275	268	263	261	256	256	255	254	254	254
IPI = 12-17	389	382	371	360	357	355	355	355	355	355
IPI = 18-23	265	260	249	245	243	241	241	241	241	240
IPI = 24-59	594	554	525	510	510	508	508	506	506	506

E-Figure 1.82: Bangladesh (2011)

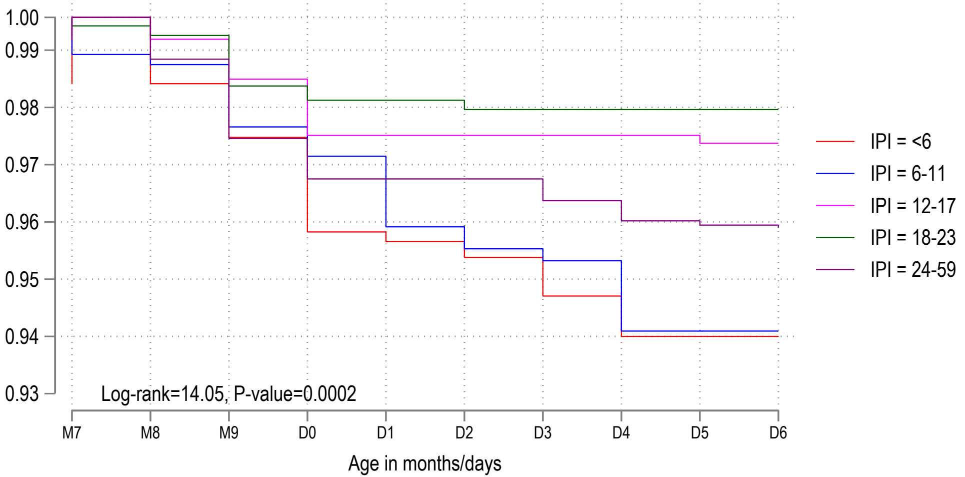

Number at risk

	M7	M8	M9	D0	D1	D2	D3	D4	D5	D6
IPI = <6	325	311	303	300	295	295	294	292	290	290
IPI = 6-11	396	380	373	364	362	357	356	355	350	350
IPI = 12-17	472	464	453	446	441	441	441	441	441	441
IPI = 18-23	392	386	374	368	367	367	366	366	366	366
IPI = 24-59	940	899	840	818	812	812	812	809	806	805

E-Figure 1.83: Bangladesh (2014)

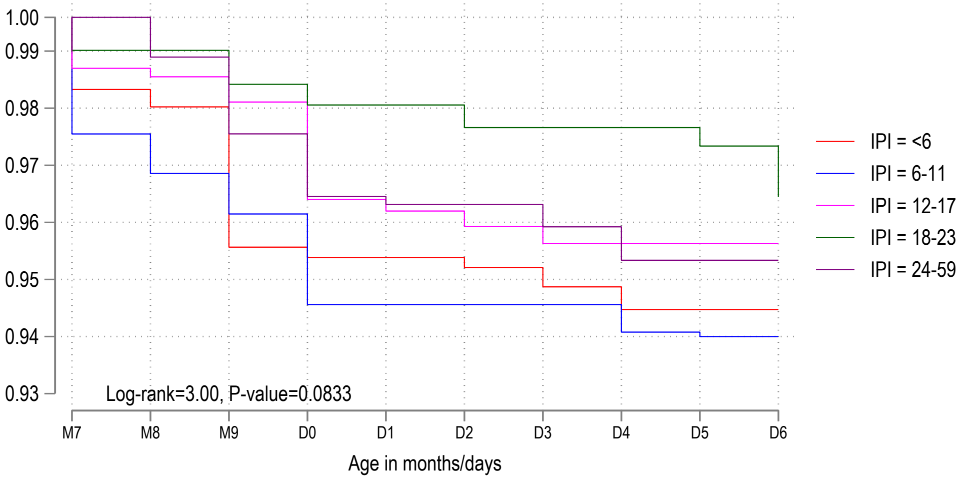

Number at risk										
IPI = <6	284	276	274	267	266	266	266	265	264	264
IPI = 6-11	319	306	294	290	285	285	285	285	283	283
IPI = 12-17	391	380	372	366	359	359	358	357	357	357
IPI = 18-23	312	304	297	287	286	286	285	285	285	284
IPI = 24-59	683	645	600	585	578	577	577	575	572	572

E-Figure 1.84: Bangladesh (2017/18)

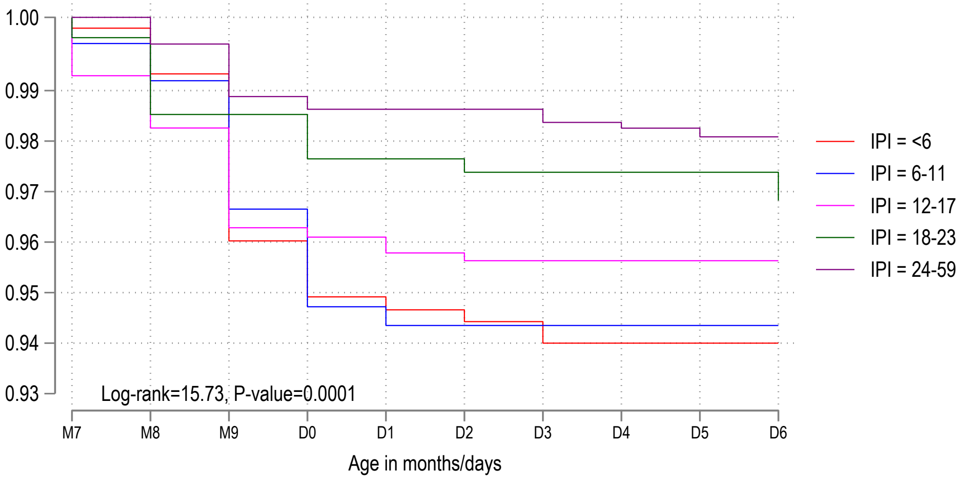

Number at risk

IPI = <6	335	328	323	310	307	306	305	304	304	304
IPI = 6-11	364	358	345	334	327	326	326	326	326	326
IPI = 12-17	406	389	374	366	366	365	364	364	364	364
IPI = 18-23	318	309	298	298	296	296	295	295	295	295
IPI = 24-59	966	928	883	865	863	863	863	860	859	858

E-Figure 1.85: Cambodia (2010/11)

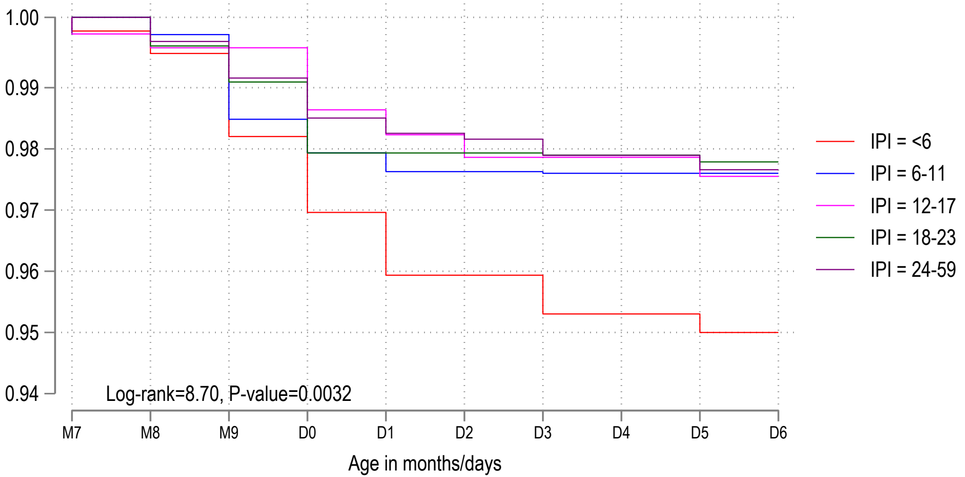

Number at risk

	M7	M8	M9	D0	D1	D2	D3	D4	D5	D6
IPI = <6	414	409	401	391	386	382	382	380	380	379
IPI = 6-11	522	515	505	484	481	480	480	480	480	480
IPI = 12-17	681	658	644	631	625	622	620	620	620	618
IPI = 18-23	514	502	492	484	478	478	478	478	478	477
IPI = 24-59	931	898	854	835	830	828	827	825	825	823

E-Figure 1.86: India (2005/6)

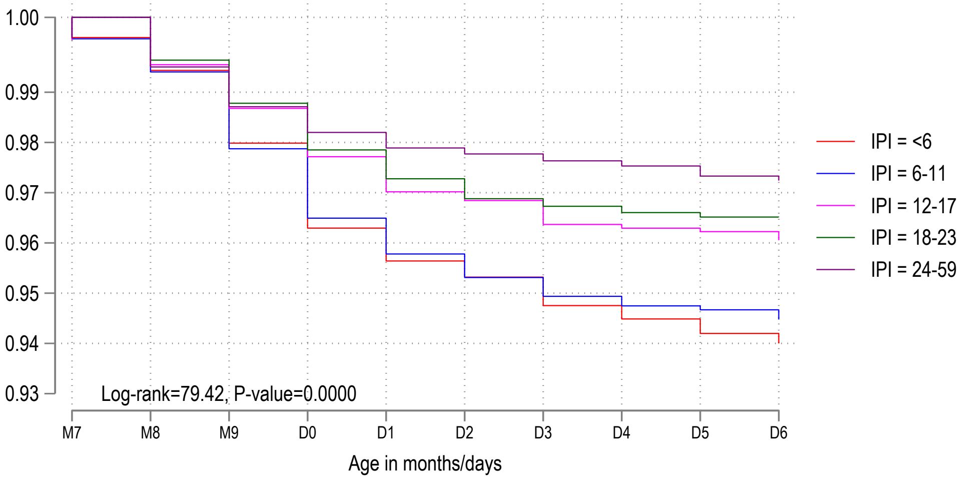

Number at risk

IPI = <6	3832	3762	3686	3578	3516	3492	3481	3460	3450	3439
IPI = 6-11	5082	4986	4872	4719	4652	4618	4595	4577	4568	4563
IPI = 12-17	6744	6606	6471	6312	6250	6205	6194	6164	6159	6151
IPI = 18-23	4402	4282	4173	4025	3987	3963	3947	3941	3936	3932
IPI = 24-59	6720	6496	6281	6057	6026	6007	5999	5991	5985	5972

E-Figure 1.87: India (2015/16)

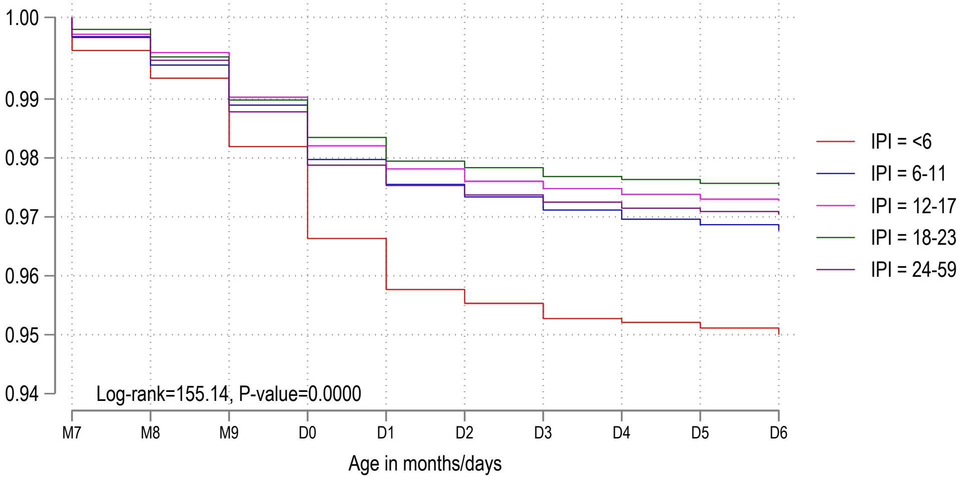

Number at risk

IPI = <6	12635	12360	12103	11847	11658	11553	11525	11494	11486	11472
IPI = 6-11	18385	17972	17531	17230	17069	16995	16958	16919	16892	16874
IPI = 12-17	22810	22298	21718	21335	21156	21071	21027	20999	20978	20959
IPI = 18-23	14979	14609	14195	13918	13829	13772	13756	13735	13728	13719
IPI = 24-59	20390	19561	18684	18123	17957	17893	17863	17841	17822	17810

E-Figure 1.88: India (2019/21)

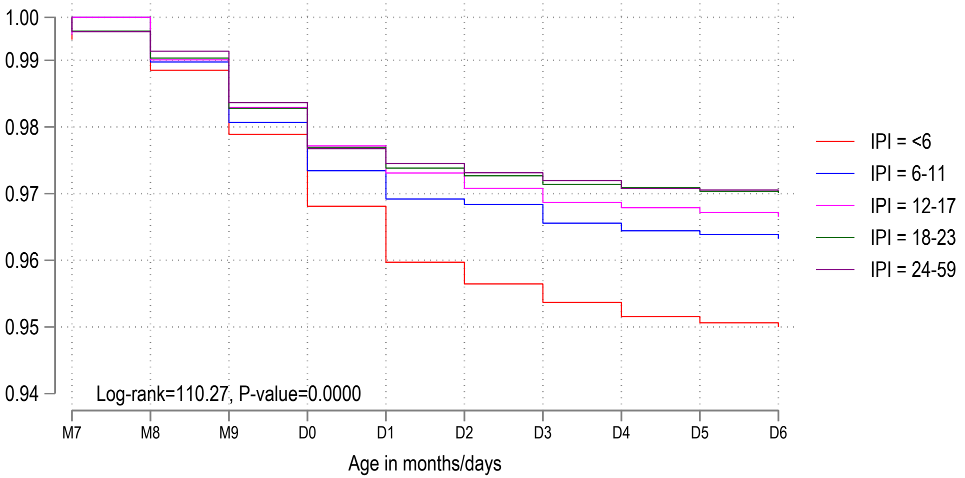

Number at risk

IPI = <6	10432	10261	10029	9835	9728	9643	9611	9583	9562	9552
IPI = 6-11	17441	17107	16700	16386	16266	16195	16182	16135	16116	16107
IPI = 12-17	20988	20563	20135	19796	19681	19599	19553	19511	19494	19479
IPI = 18-23	14405	14075	13697	13452	13372	13330	13314	13296	13289	13282
IPI = 24-59	22898	22069	21203	20645	20501	20454	20425	20400	20375	20371

E-Figure 1.89: Indonesia (2002/3)

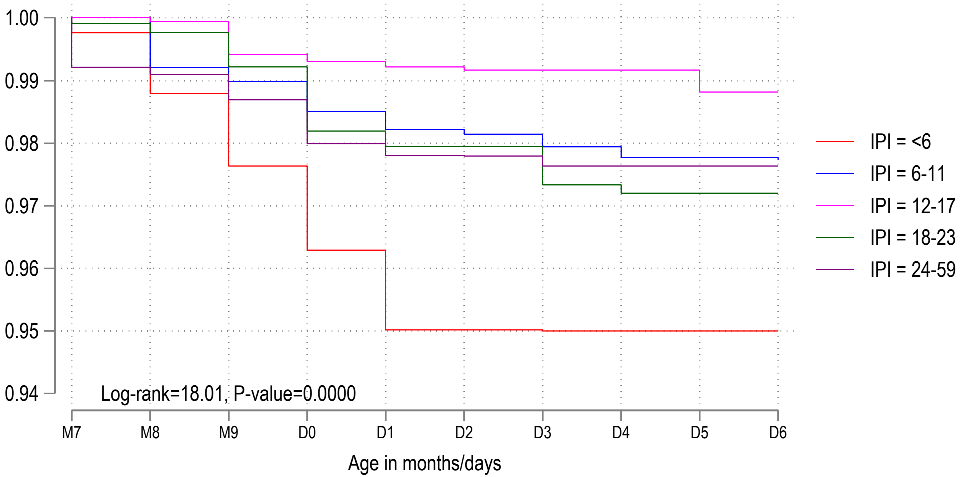

Number at risk

	M7	M8	M9	D0	D1	D2	D3	D4	D5	D6
IPI = <6	492	483	470	450	444	438	438	438	438	438
IPI = 6-11	683	675	662	657	654	652	652	650	649	649
IPI = 12-17	811	790	780	761	761	760	760	760	760	757
IPI = 18-23	578	567	552	542	537	535	535	532	531	531
IPI = 24-59	1539	1468	1411	1370	1360	1357	1357	1355	1355	1355

E-Figure 1.90: Indonesia (2007)

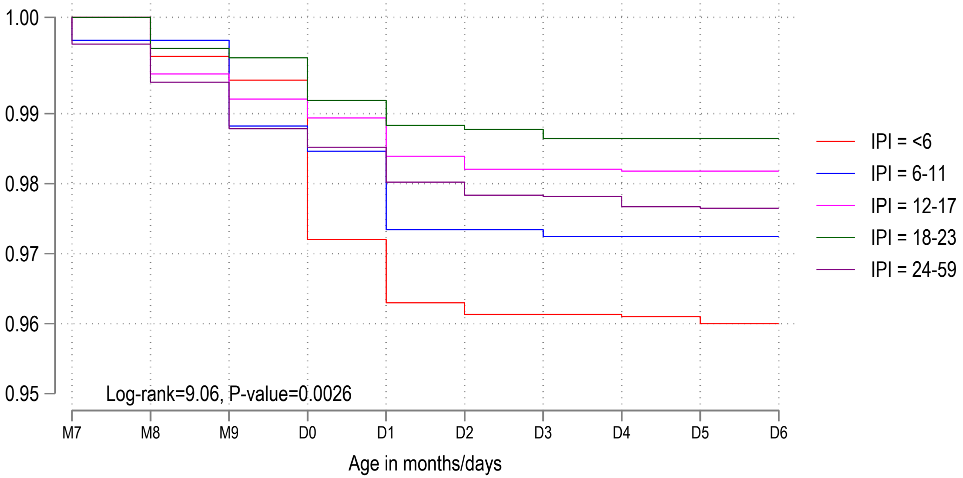

Number at risk

	M7	M8	M9	D0	D1	D2	D3	D4	D5	D6
IPI = <6	675	663	651	645	630	624	623	623	623	622
IPI = 6-11	657	646	637	626	624	617	617	616	616	616
IPI = 12-17	778	764	747	733	731	727	725	725	725	725
IPI = 18-23	551	540	533	522	519	517	516	516	516	516
IPI = 24-59	1294	1213	1150	1113	1110	1104	1102	1102	1100	1100

E-Figure 1.91: Indonesia (2012)

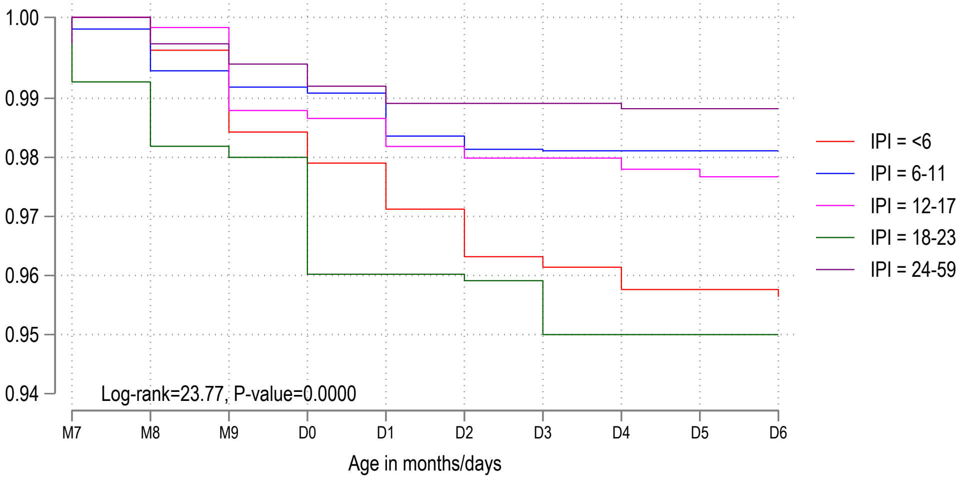

Number at risk

IPI = <6	662	654	636	614	611	606	601	600	597	597
IPI = 6-11	638	618	603	596	596	591	590	590	590	590
IPI = 12-17	690	677	663	647	646	643	641	641	640	639
IPI = 18-23	487	463	449	444	435	435	434	430	430	430
IPI = 24-59	1121	1067	987	957	954	951	951	951	950	950

E-Figure 1.92: Indonesia (2017)

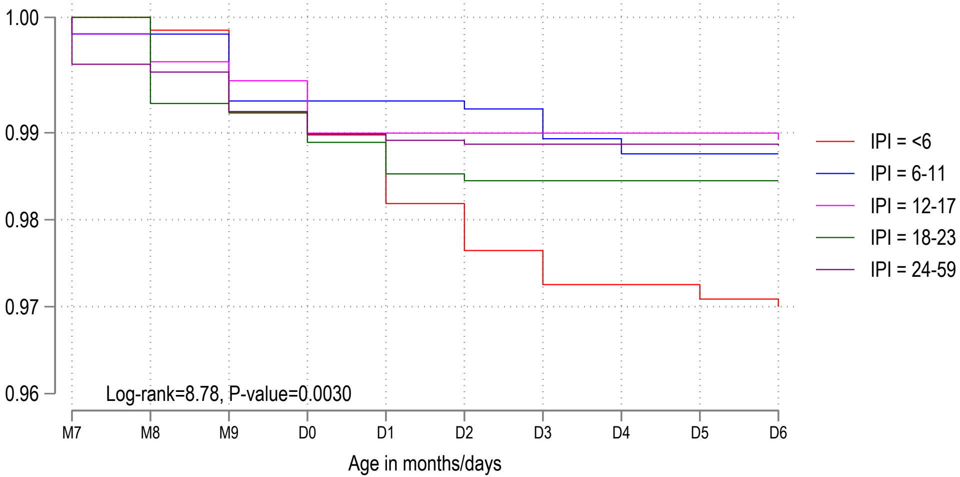

Number at risk

	M7	M8	M9	D0	D1	D2	D3	D4	D5	D6
IPI = <6	660	640	628	615	614	609	605	603	603	601
IPI = 6-11	613	598	585	577	577	577	577	575	574	574
IPI = 12-17	616	606	587	583	580	580	580	580	580	580
IPI = 18-23	494	485	465	460	459	457	457	457	457	457
IPI = 24-59	1396	1332	1247	1219	1216	1215	1214	1214	1214	1214

E-Figure 1.93: Myanmar (2015/16)

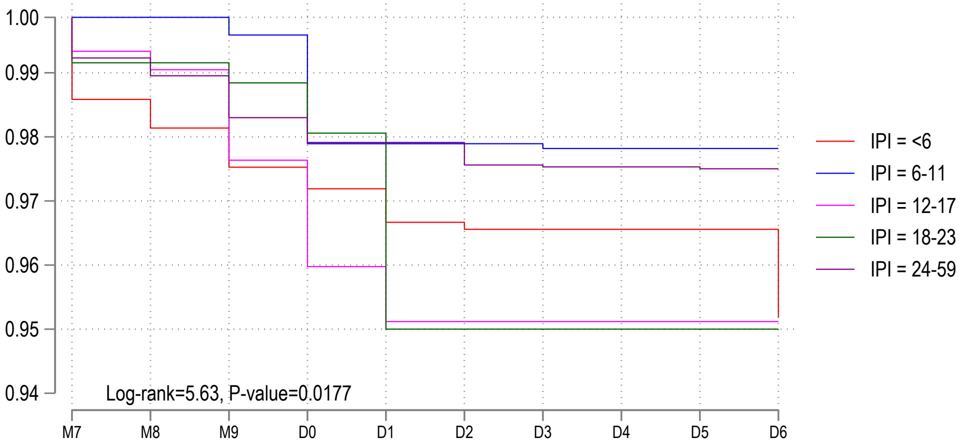

Number at risk

	M7	M8	M9	D0	D1	D2	D3	D4	D5	D6
IPI = <6	127	124	121	121	120	120	120	120	120	120
IPI = 6-11	190	185	182	181	178	178	178	178	178	178
IPI = 12-17	268	265	258	253	249	247	247	247	247	247
IPI = 18-23	186	184	181	179	178	172	172	172	172	172
IPI = 24-59	510	493	472	459	457	457	455	455	455	455

E-Figure 1.94: Nepal (2006)

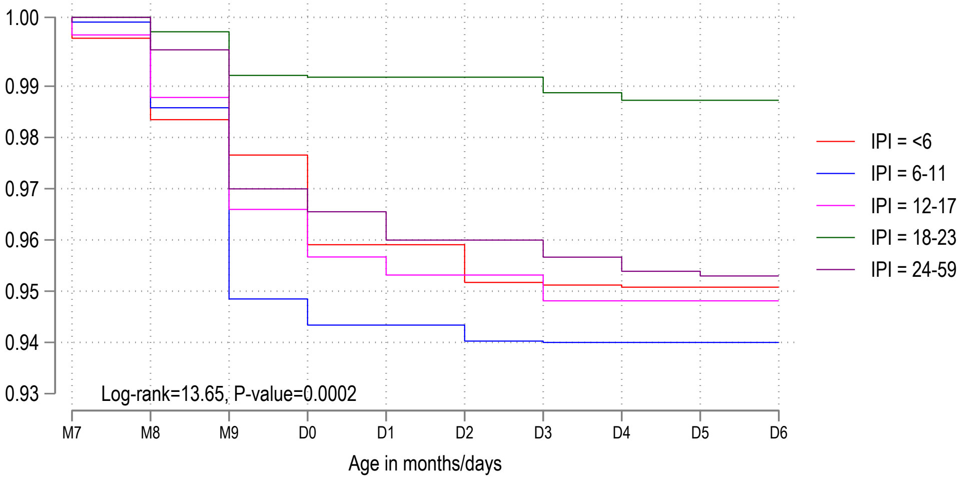

Number at risk

IPI = <6	236	234	223	219	215	215	214	214	214	214
IPI = 6-11	415	411	399	382	380	380	379	379	379	379
IPI = 12-17	671	661	645	627	621	619	619	616	616	616
IPI = 18-23	484	478	471	460	460	460	460	458	458	458
IPI = 24-59	797	775	739	708	705	701	701	698	696	696

E-Figure 1.95: Nepal (2011)

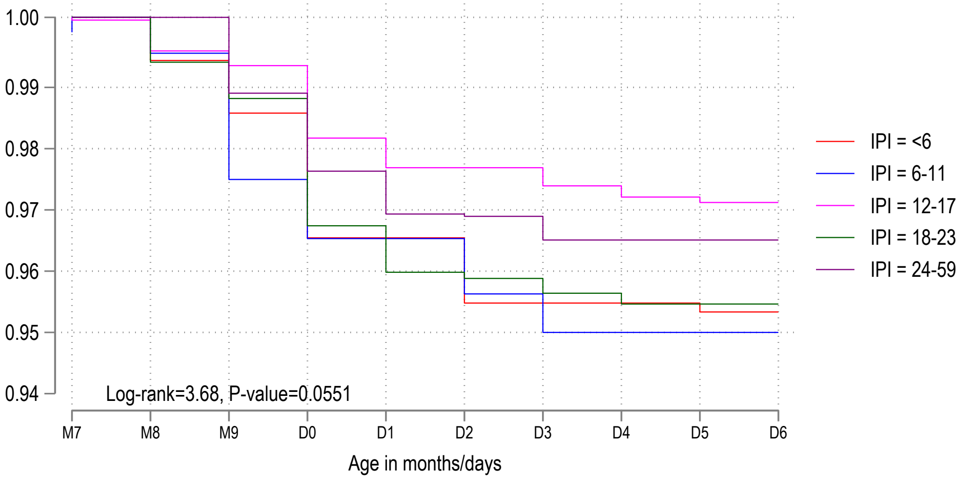

Number at risk

IPI = <6	247	243	237	232	227	227	225	225	225	225
IPI = 6-11	349	343	334	326	322	322	319	317	317	317
IPI = 12-17	589	580	573	565	559	556	556	554	553	553
IPI = 18-23	349	343	332	327	320	317	317	316	316	316
IPI = 24-59	696	681	660	643	635	630	630	627	627	627

E-Figure 1.96: Nepal (2016/17)

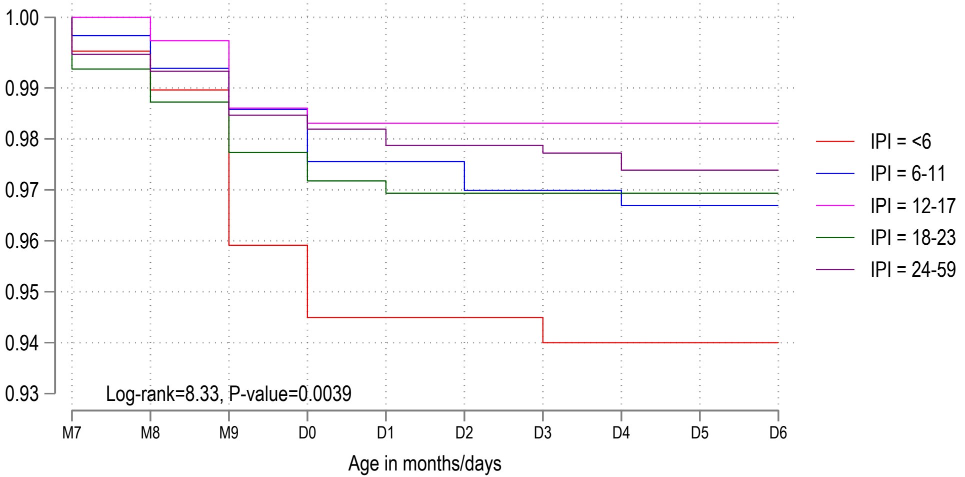

Number at risk

IPI = <6	243	240	236	228	225	225	225	224	224	224
IPI = 6-11	331	324	317	312	309	309	307	307	306	306
IPI = 12-17	364	358	338	332	331	331	331	331	331	331
IPI = 18-23	284	277	266	263	262	261	261	261	261	261
IPI = 24-59	504	483	457	445	444	442	442	442	440	440

E-Figure 1.97: Nepal (2021/22)

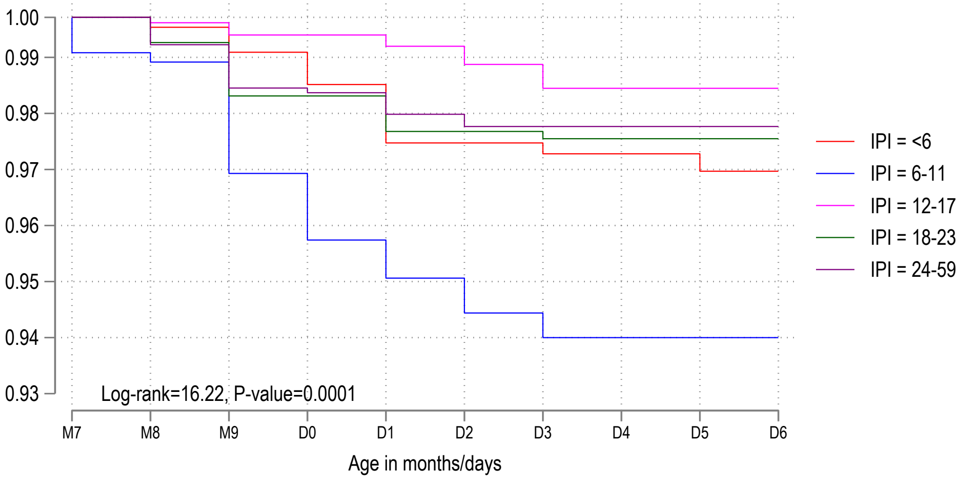

Number at risk

IPI = <6	235	232	230	228	227	225	225	224	224	223
IPI = 6-11	331	323	320	308	304	302	300	299	299	299
IPI = 12-17	425	421	412	408	408	407	406	404	404	404
IPI = 18-23	290	282	275	268	268	266	266	266	266	266
IPI = 24-59	517	496	474	464	464	462	461	461	461	461

E-Figure 1.98: Pakistan (2012/13)

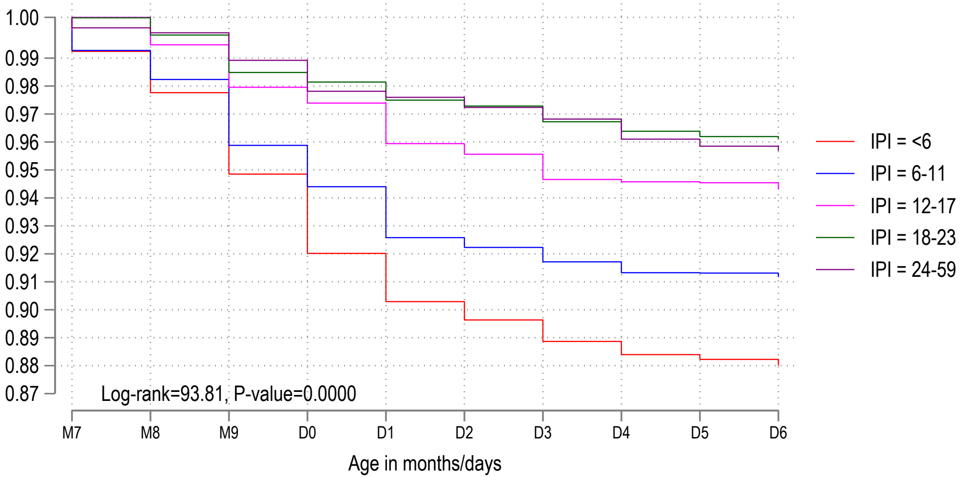

Number at risk

IPI = <6	1485	1453	1408	1356	1315	1290	1281	1270	1263	1260
IPI = 6-11	1697	1634	1577	1537	1513	1484	1478	1470	1463	1463
IPI = 12-17	1680	1655	1604	1574	1565	1542	1536	1521	1520	1519
IPI = 18-23	1100	1058	1031	1009	1005	999	996	991	987	985
IPI = 24-59	1476	1410	1345	1316	1302	1299	1294	1288	1279	1275

E-Figure 1.99: Pakistan (2017/18)

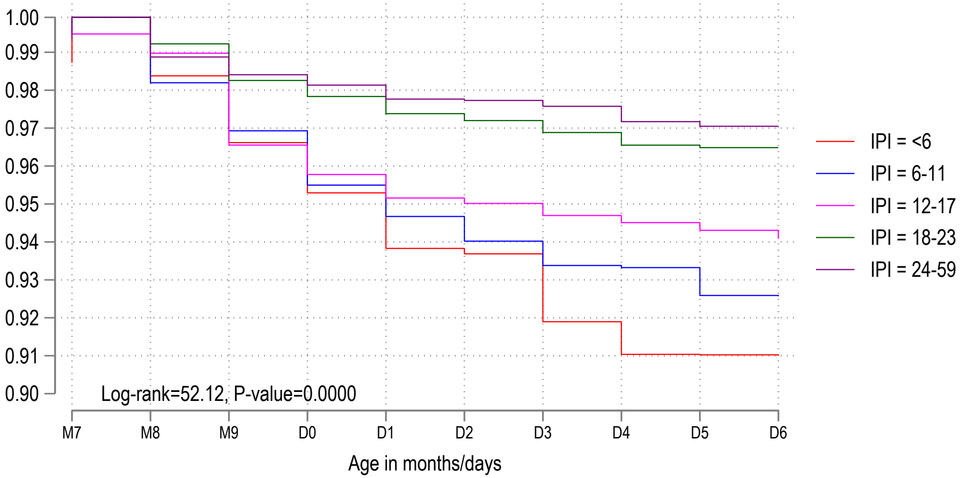

Number at risk

IPI = <6	1179	1159	1136	1115	1099	1083	1081	1060	1050	1050
IPI = 6-11	1410	1373	1335	1314	1295	1284	1275	1266	1265	1255
IPI = 12-17	1382	1360	1334	1294	1284	1275	1273	1269	1267	1264
IPI = 18-23	879	854	821	808	805	801	800	797	794	794
IPI = 24-59	1263	1216	1150	1141	1138	1133	1133	1131	1127	1125

E-Figure 1.100: Philippines (2003)

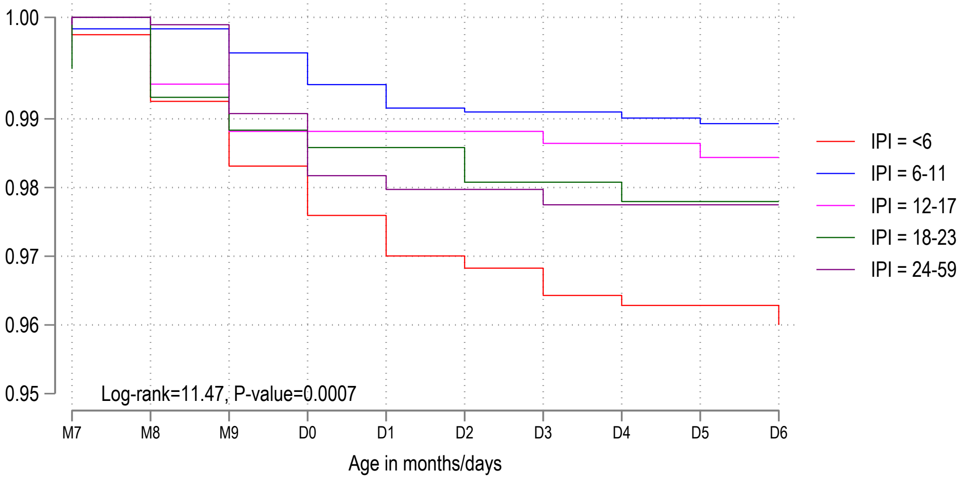

Number at risk

IPI = <6	543	532	524	516	512	509	508	506	505	505
IPI = 6-11	753	739	726	711	707	705	705	705	704	703
IPI = 12-17	707	684	665	654	654	654	654	653	653	651
IPI = 18-23	437	423	409	406	405	405	403	403	402	402
IPI = 24-59	574	553	527	507	502	501	501	500	500	500

E-Figure 1.101: Philippines (2022)

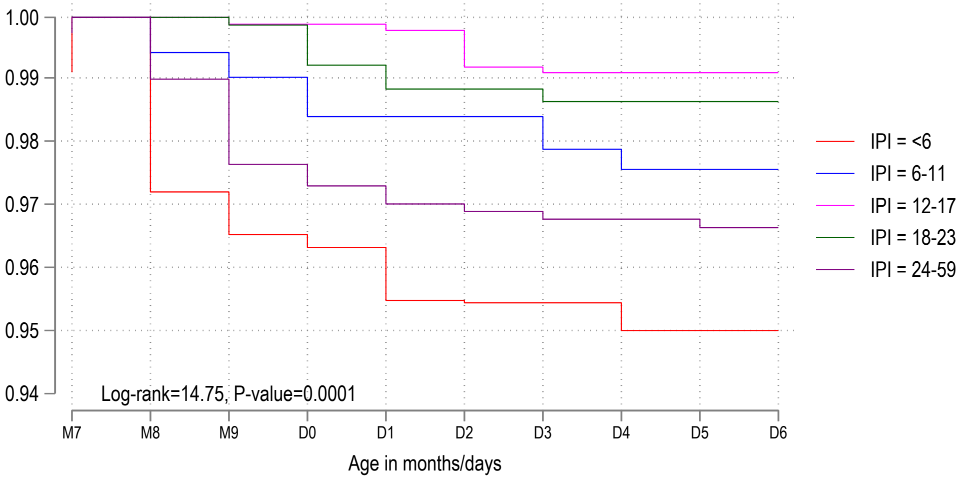

Number at risk

IPI = <6	305	301	289	287	286	284	284	284	282	282
IPI = 6-11	405	403	392	390	387	387	387	385	384	384
IPI = 12-17	414	406	400	399	399	398	396	396	396	396
IPI = 18-23	290	284	281	281	279	278	278	278	278	278
IPI = 24-59	570	536	504	488	486	485	484	483	483	483

E-Figure 1.102: Timor-Leste (2009/10)

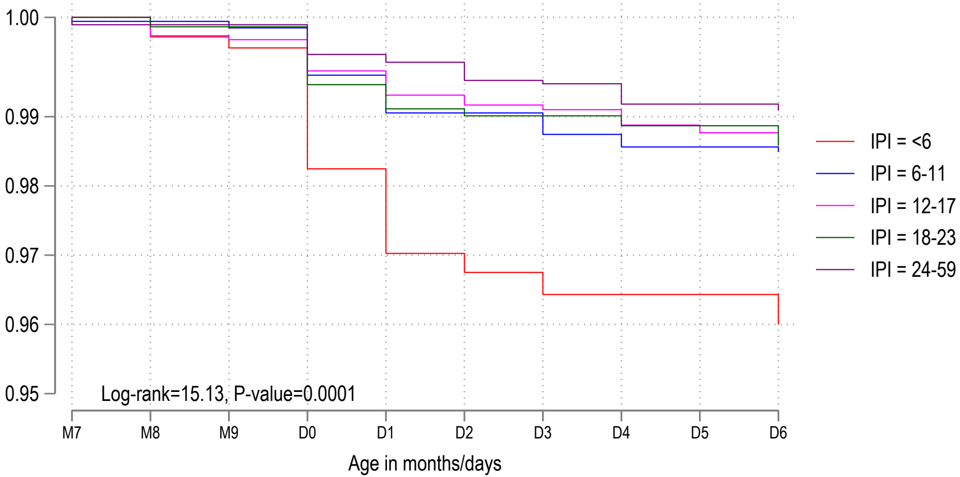

Number at risk

IPI = <6	367	363	359	355	349	345	344	343	343	343
IPI = 6-11	989	979	965	958	951	946	946	943	941	941
IPI = 12-17	1655	1629	1593	1589	1582	1576	1574	1573	1569	1567
IPI = 18-23	1148	1123	1089	1079	1070	1066	1065	1065	1064	1064
IPI = 24-59	1291	1255	1220	1208	1202	1201	1198	1197	1194	1194

E-Figure 1.103: Bolivia (2003/4)

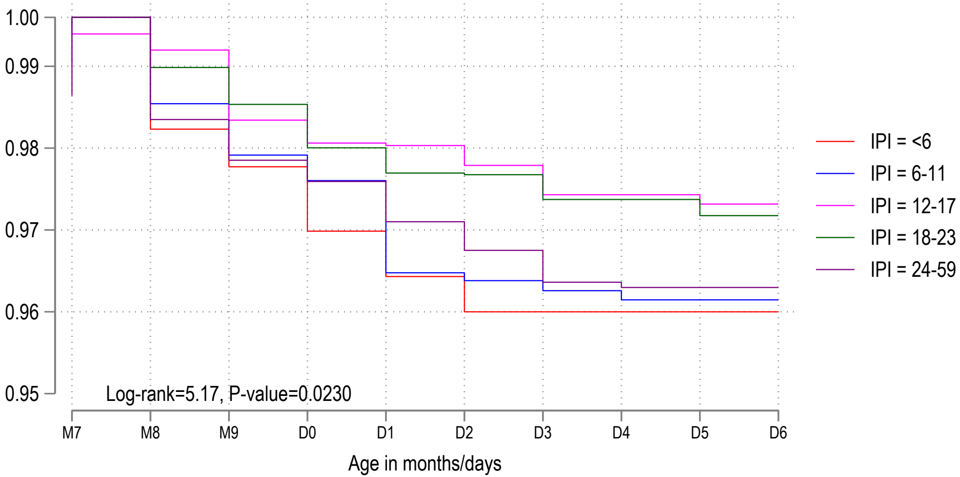

Number at risk

IPI = <6	532	529	516	512	508	505	503	503	503	501
IPI = 6-11	954	939	917	905	902	892	891	890	889	888
IPI = 12-17	1339	1317	1293	1274	1270	1270	1267	1262	1262	1260
IPI = 18-23	927	909	889	876	871	869	868	866	866	864
IPI = 24-59	1126	1073	1026	1016	1013	1008	1005	1001	1000	1000

E-Figure 1.104: Bolivia (2008)

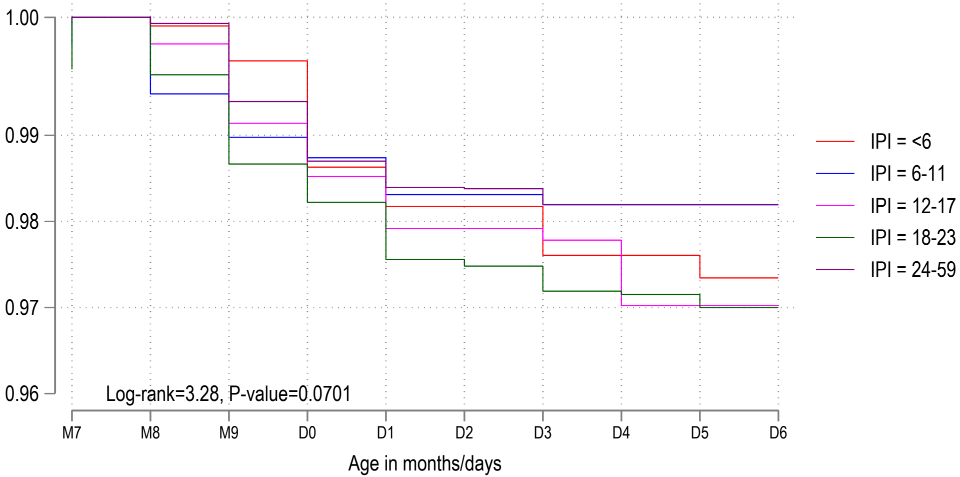

Number at risk

IPI = <6	352	345	338	336	332	330	330	328	328	325
IPI = 6-11	628	613	595	588	586	584	584	583	583	583
IPI = 12-17	860	850	827	813	808	803	803	802	795	795
IPI = 18-23	560	542	527	520	518	514	514	512	512	511
IPI = 24-59	678	648	608	594	590	588	588	587	587	587

E-Figure 1.105: Colombia (2005)

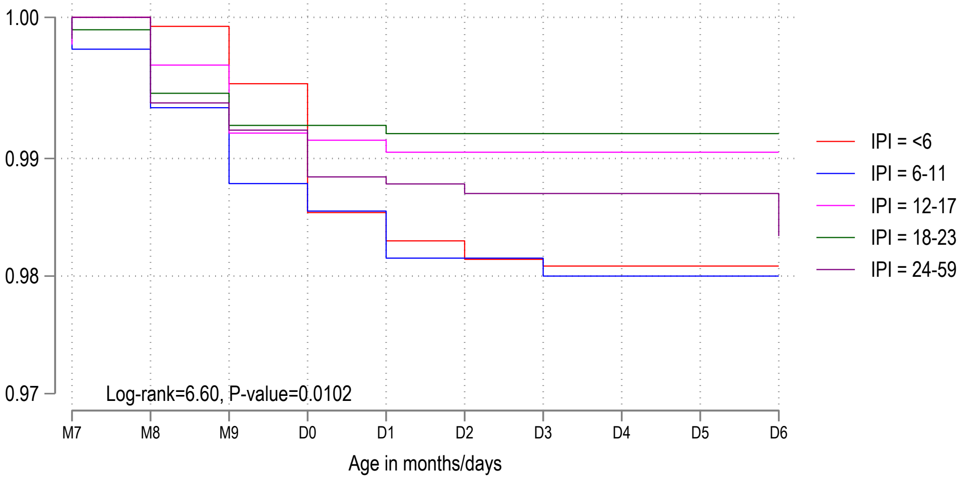

Number at risk

	M7	M8	M9	D0	D1	D2	D3	D4	D5	D6
IPI = <6	612	600	591	584	577	576	575	575	575	575
IPI = 6-11	1085	1065	1042	1030	1028	1024	1024	1022	1022	1022
IPI = 12-17	1174	1150	1127	1116	1115	1114	1114	1114	1114	1114
IPI = 18-23	750	742	716	710	710	709	709	709	709	709
IPI = 24-59	1433	1386	1352	1344	1338	1337	1336	1336	1336	1336

E-Figure 1.106: Dominican Republic (2002)

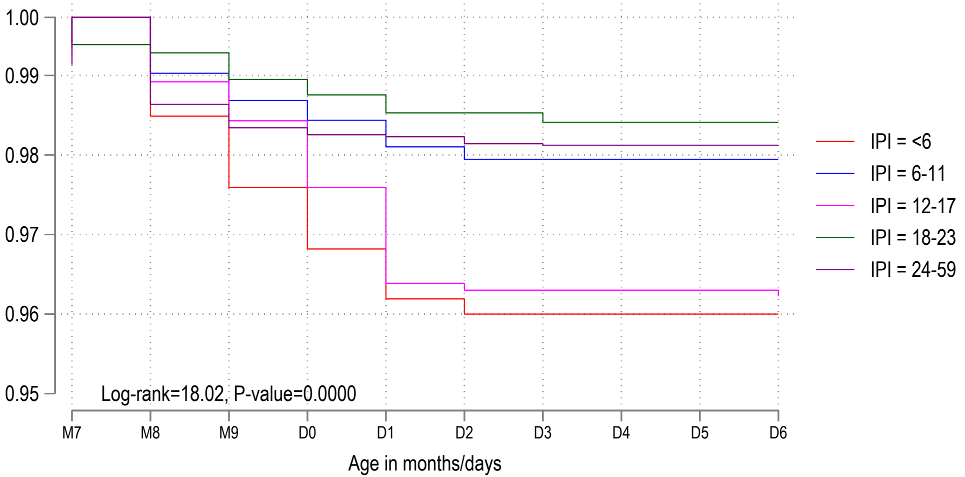

Number at risk

IPI = <6	826	808	787	779	773	768	766	766	766	766
IPI = 6-11	1113	1091	1070	1066	1063	1059	1058	1058	1058	1058
IPI = 12-17	1016	989	962	953	945	934	933	933	933	929
IPI = 18-23	637	613	591	587	586	585	585	584	584	584
IPI = 24-59	1094	1046	993	980	979	979	978	978	978	978

E-Figure 1.107: Guatemala (2014/15)

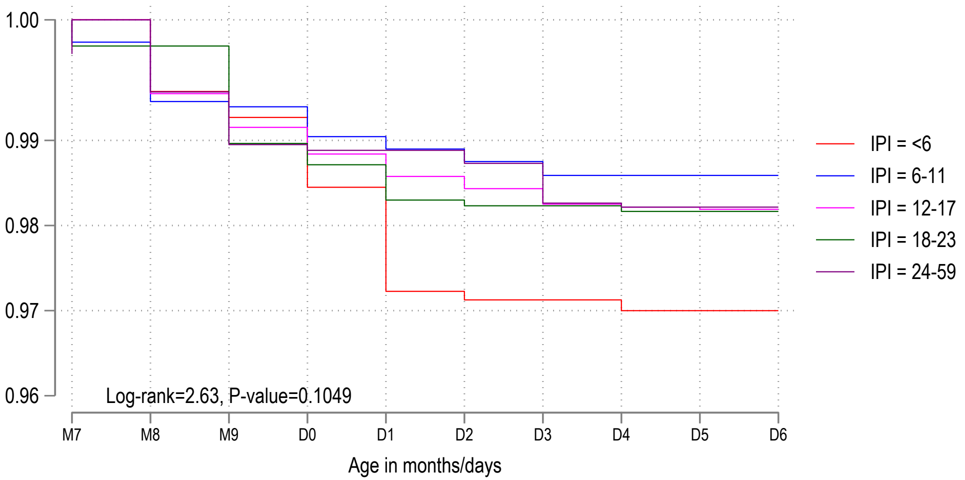

Number at risk

	M7	M8	M9	D0	D1	D2	D3	D4	D5	D6
IPI = <6	341	334	325	324	322	318	317	317	317	317
IPI = 6-11	824	810	784	782	779	778	777	776	776	776
IPI = 12-17	1300	1271	1240	1232	1228	1225	1223	1221	1221	1220
IPI = 18-23	994	969	939	925	923	919	919	919	918	918
IPI = 24-59	1710	1642	1558	1537	1536	1536	1534	1526	1526	1526

E-Figure 1.108: Honduras (2005/6)

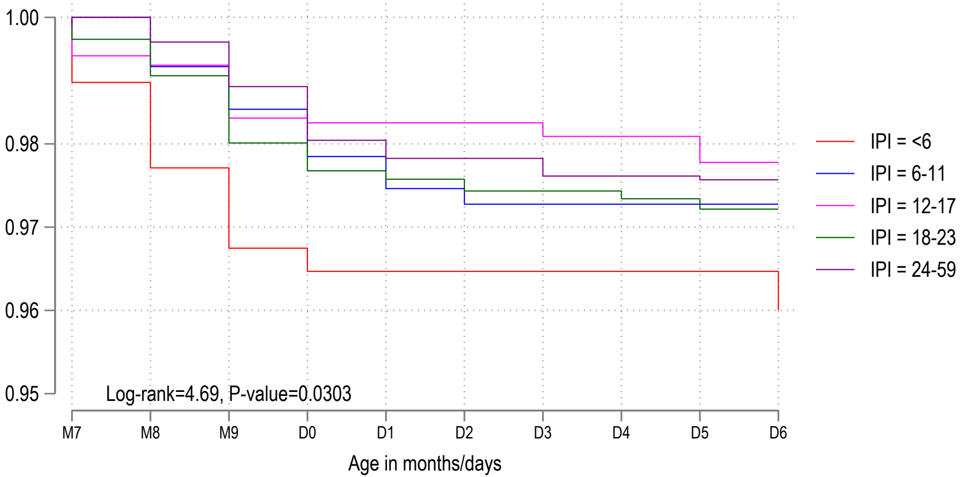

Number at risk

IPI = <6	392	385	376	371	370	370	370	370	370	370
IPI = 6-11	765	758	741	731	727	724	723	723	723	723
IPI = 12-17	932	919	897	884	884	884	884	883	883	880
IPI = 18-23	851	838	822	812	809	808	807	807	806	805
IPI = 24-59	1465	1425	1365	1339	1330	1327	1327	1324	1324	1323

E-Figure 1.109: Honduras (2011/12)

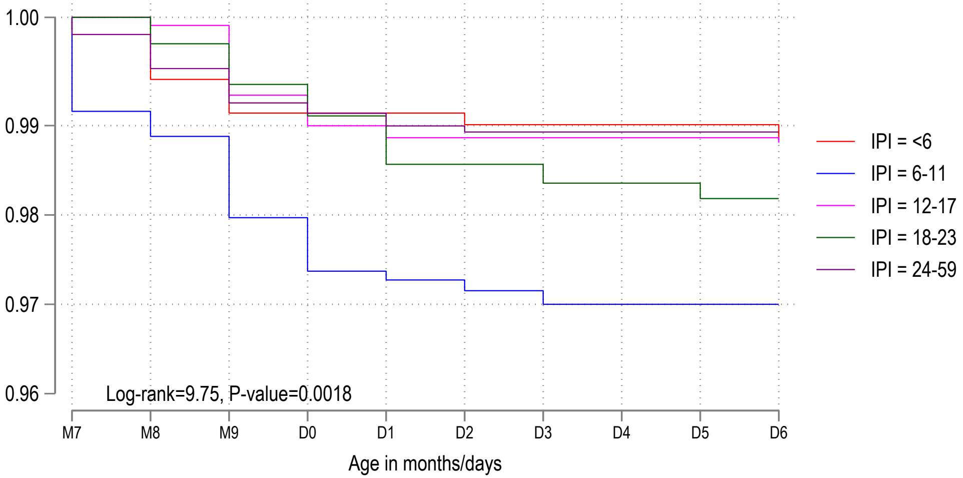

Number at risk

IPI = <6	290	289	279	278	278	278	278	278	278	278
IPI = 6-11	607	586	566	558	554	554	553	552	552	552
IPI = 12-17	712	708	690	683	680	679	679	679	679	679
IPI = 18-23	556	542	527	524	522	519	519	518	518	517
IPI = 24-59	1284	1241	1168	1148	1147	1145	1145	1145	1145	1145

E-Figure 1.110: Nicaragua (2001)

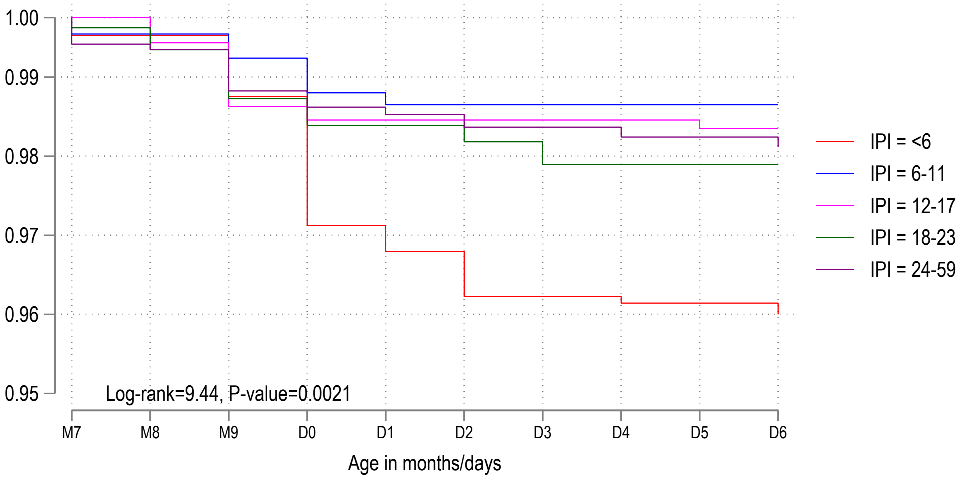

Number at risk

IPI = <6	353	349	346	342	336	335	333	333	333	333
IPI = 6-11	546	538	530	524	522	521	521	521	521	521
IPI = 12-17	650	631	611	601	600	600	600	600	600	600
IPI = 18-23	429	420	409	402	401	401	400	399	399	399
IPI = 24-59	593	573	535	523	522	521	521	521	520	520

E-Figure 1.111: Peru (2000)

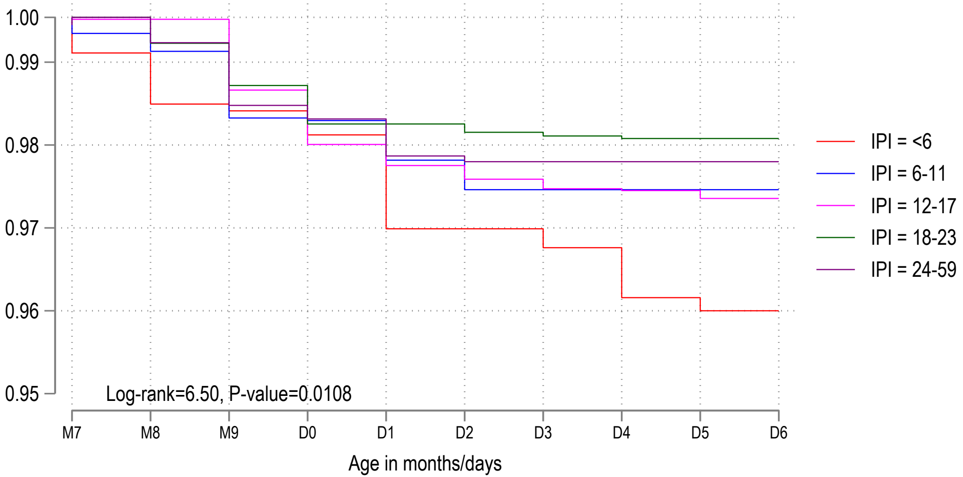

Number at risk

	M7	M8	M9	D0	D1	D2	D3	D4	D5	D6
IPI = <6	418	408	402	400	399	394	394	394	391	390
IPI = 6-11	792	780	769	759	758	755	752	752	752	752
IPI = 12-17	1031	1020	997	978	971	969	967	966	966	965
IPI = 18-23	823	804	778	770	766	766	766	765	765	765
IPI = 24-59	1231	1181	1116	1096	1094	1089	1089	1089	1089	1088

E-Figure 1.112: Peru (2007/8)

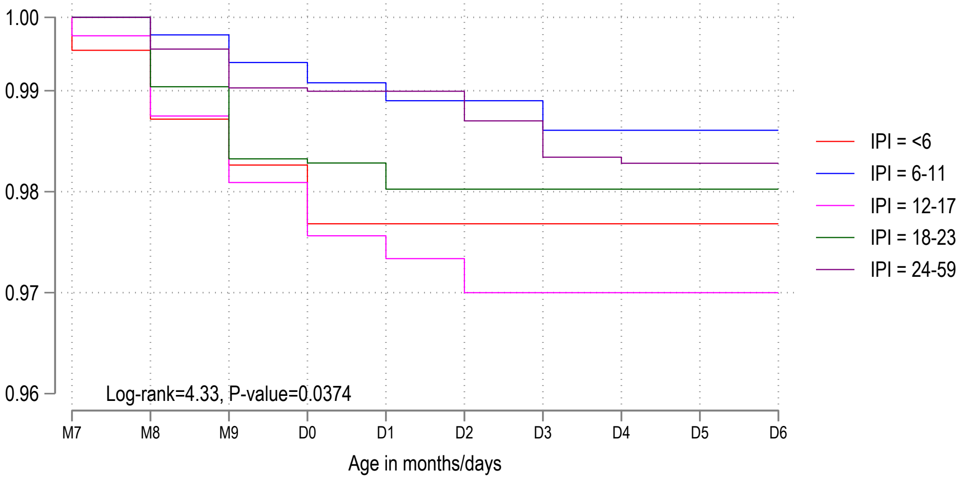

Number at risk

	M7	M8	M9	D0	D1	D2	D3	D4	D5	D6
IPI = <6	236	232	224	223	221	221	221	221	221	221
IPI = 6-11	409	397	383	380	379	378	378	377	377	377
IPI = 12-17	564	554	538	531	528	527	525	525	525	525
IPI = 18-23	450	445	427	421	421	420	420	420	420	420
IPI = 24-59	698	668	623	616	616	616	614	612	612	612

E-Figure 1.113: Peru (2009)

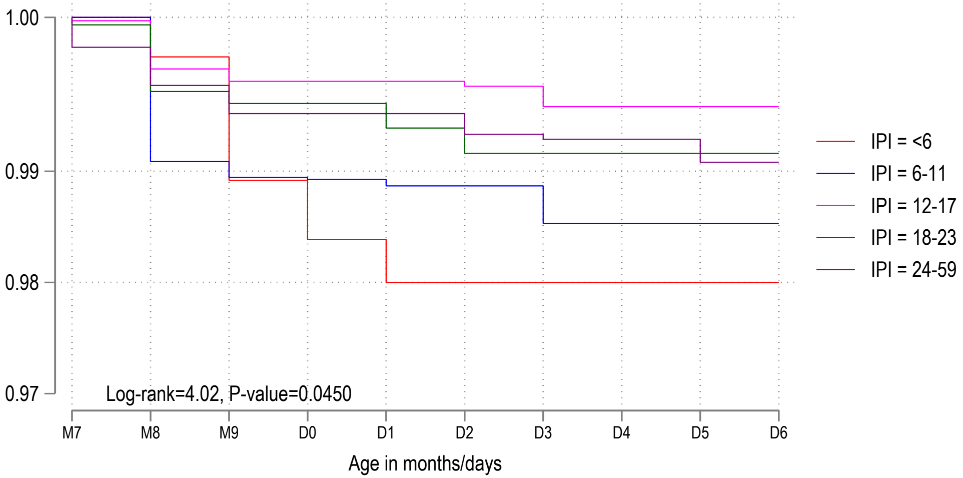

Number at risk

IPI = <6	236	233	229	226	225	224	224	224	224	224
IPI = 6-11	462	457	444	443	443	443	443	442	442	442
IPI = 12-17	621	605	592	588	588	588	588	587	587	587
IPI = 18-23	456	443	437	437	437	436	435	435	435	435
IPI = 24-59	819	782	735	724	724	724	723	722	722	721

E-Figure 2: Survey-specific hazard ratios (HRs) of perinatal mortality, by IPI (months) following livebirth

A) IPI of less than 6 months versus IPI 18-23 months

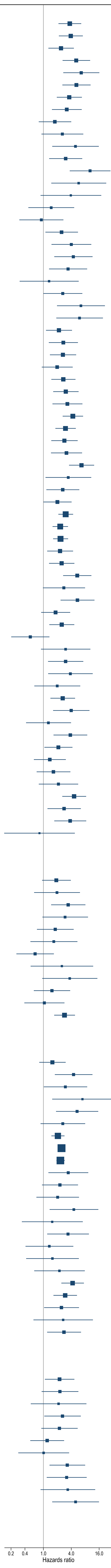

Region Survey	HR	95% CI
**Sub-Saharan Africa**		
Angola (2015/16)	3.72	[2.15, 6.44]
Benin (2006)	3.91	[2.19, 6.99]
Benin (2011/12)	2.41	[1.30, 4.47]
Benin (2017/18)	5.13	[2.63, 10.02]
Burkina Faso (2003)	6.57	[2.72, 15.87]
Burkina Faso (2010)	5.17	[2.60, 10.26]
Burundi (2010/11)	3.62	[1.97, 6.65]
Ethiopia (2005)	3.20	[1.55, 6.61]
Ethiopia (2011)	1.77	[0.80, 3.92]
Ethiopia (2016)	2.57	[0.92, 7.15]
Gambia (2013)	4.93	[1.57, 15.43]
Gambia (2019/20)	3.02	[1.35, 6.77]
Ghana (2003)	10.16	[3.75, 27.51]
Ghana (2008)	5.79	[1.50, 22.32]
Ghana (2014)	3.92	[0.88, 17.44]
Guinea (2005)	1.48	[0.48, 4.54]
Guinea (2018)	0.90	[0.31, 2.66]
Kenya (2003)	2.48	[1.12, 5.49]
Kenya (2008/9)	4.01	[1.51, 10.64]
Kenya (2014)	4.44	[1.74, 11.32]
Kenya (2022)	3.41	[1.34, 8.65]
Lesotho (2009/10)	1.33	[0.31, 5.68]
Liberia (2013)	2.62	[1.01, 6.80]
Liberia (2019/20)	6.46	[2.00, 20.94]
Madagascar (2003/4)	6.04	[1.93, 18.98]
Madagascar (2008/9)	2.17	[1.15, 4.07]
Madagascar (2021)	2.69	[1.32, 5.46]
Malawi (2000)	2.65	[1.40, 5.00]
Malawi (2004/5)	1.99	[0.93, 4.22]
Malawi (2010)	2.70	[1.50, 4.85]
Malawi (2015/16)	3.05	[1.65, 5.66]
Mali (2001)	3.30	[1.59, 6.85]
Mali (2006)	4.32	[2.64, 7.07]
Mali (2012/13)	3.01	[1.84, 4.92]
Mali (2018)	2.85	[1.50, 5.40]
Mozambique (2003/4)	3.15	[1.48, 6.71]
Mozambique (2011)	6.66	[3.63, 12.23]
Namibia (2006/7)	3.46	[1.13, 10.61]
Niger (2006)	2.62	[1.17, 5.86]
Niger (2012)	2.01	[1.01, 3.99]
Nigeria (2008)	3.03	[2.13, 4.30]
Nigeria (2013)	2.31	[1.60, 3.34]
Nigeria (2018)	2.35	[1.64, 3.36]
Rwanda (2000)	2.29	[1.22, 4.30]
Rwanda (2005)	2.48	[1.34, 4.58]
Rwanda (2010/11)	5.38	[2.71, 10.69]
Rwanda (2014/15)	2.78	[0.99, 7.79]
Rwanda (2019/20)	5.45	[2.38, 12.47]
Senegal (2005)	1.83	[0.91, 3.72]
Senegal (2010/11)	2.49	[1.36, 4.56]
Senegal (2015)	0.52	[0.20, 1.33]
Senegal (2016)	3.02	[0.90, 10.15]
Senegal (2018)	3.02	[1.28, 7.14]
Senegal (2019)	3.83	[1.28, 11.41]
Sierra Leone (2008)	1.99	[0.65, 6.14]
Sierra Leone (2013)	2.61	[1.44, 4.75]
Tanzania (2004/5)	4.00	[1.64, 9.72]
Tanzania (2010)	1.29	[0.43, 3.88]
Tanzania (2015/16)	3.82	[1.68, 8.70]
Uganda (2000/1)	2.11	[1.06, 4.17]
Uganda (2006)	1.38	[0.63, 3.00]
Uganda (2011)	1.65	[0.72, 3.75]
Zambia (2007)	2.11	[0.81, 5.54]
Zambia (2013/14)	4.62	[2.59, 8.23]
Zambia (2018/19)	2.80	[1.24, 6.33]
Zimbabwe (2010/11)	3.79	[1.74, 8.27]
Zimbabwe (2015)	0.82	[0.14, 4.69]
**N. Africa/W. Asia/Europe**		
Egypt (2000)	1.91	[0.95, 3.84]
Egypt (2003)	1.97	[0.64, 6.04]
Egypt (2005)	3.44	[1.49, 7.95]
Egypt (2008)	2.95	[0.96, 9.03]
Egypt (2014)	1.81	[0.74, 4.43]
Jordan (2002)	1.68	[0.53, 5.34]
Jordan (2009)	0.66	[0.27, 1.65]
Jordan (2012)	2.50	[0.54, 11.67]
Jordan (2017/18)	3.71	[0.96, 14.39]
Morocco (2003/4)	1.53	[0.63, 3.72]
Turkey (2003/4)	1.05	[0.39, 2.80]
Yemen (2013)	2.86	[1.73, 4.72]
**Central, South & Southeast Asia**		
Bangladesh (2004)	1.57	[0.82, 2.98]
Bangladesh (2007)	4.49	[1.79, 11.28]
Bangladesh (2011)	2.99	[1.03, 8.67]
Bangladesh (2014)	6.99	[1.57, 31.11]
Bangladesh (2017/18)	5.34	[1.90, 15.02]
Cambodia (2010/11)	2.62	[0.88, 7.83]
India (2005/6)	2.06	[1.51, 2.81]
India (2015/16)	2.47	[2.09, 2.93]
India (2019/21)	2.34	[1.93, 2.83]
Indonesia (2002/3)	3.45	[1.30, 9.13]
Indonesia (2007)	2.28	[0.94, 5.50]
Indonesia (2012)	2.03	[0.71, 5.77]
Indonesia (2017)	4.56	[1.38, 15.07]
Myanmar (2015/16)	1.55	[0.35, 6.96]
Nepal (2006)	3.40	[1.22, 9.45]
Nepal (2011)	1.35	[0.42, 4.35]
Nepal (2016/17)	1.57	[0.43, 5.72]
Nepal (2021/22)	2.22	[0.64, 7.71]
Pakistan (2012/13)	4.27	[2.48, 7.36]
Pakistan (2017/18)	2.95	[1.67, 5.22]
Philippines (2003)	2.46	[1.05, 5.76]
Philippines (2022)	2.67	[0.62, 11.52]
Timor-Leste (2009/10)	2.79	[1.22, 6.38]
**Latin America & Caribbean**		
Bolivia (2003/4)	2.24	[1.09, 4.58]
Bolivia (2008)	2.28	[0.93, 5.60]
Colombia (2005)	2.13	[0.54, 8.34]
Dominican Republic (2002)	2.59	[1.06, 6.32]
Guatemala (2014/15)	2.22	[0.91, 5.40]
Honduras (2005/6)	1.20	[0.53, 2.75]
Honduras (2011/12)	1.01	[0.29, 3.53]
Nicaragua (2001)	3.28	[1.37, 7.84]
Peru (2000)	3.18	[1.20, 8.46]
Peru (2007/8)	3.37	[0.88, 12.91]
Peru (2009)	4.95	[1.57, 15.59]

B) IPI 6-11 months versus IPI 18-23 months

Region / Survey	HR	95% CI
**Sub-Saharan Africa**		
Angola (2015/16)	1.23	[0.64, 2.35]
Benin (2006)	1.75	[1.08, 2.84]
Benin (2011/12)	1.74	[0.98, 3.11]
Benin (2017/18)	1.95	[1.11, 3.41]
Burkina Faso (2003)	2.58	[1.13, 5.91]
Burkina Faso (2010)	1.72	[0.97, 3.04]
Burundi (2010/11)	1.37	[0.73, 2.58]
Ethiopia (2005)	2.05	[1.02, 4.09]
Ethiopia (2011)	0.96	[0.45, 2.02]
Ethiopia (2016)	3.67	[1.49, 9.01]
Gambia (2013)	5.52	[2.52, 12.10]
Gambia (2019/20)	2.05	[0.80, 5.26]
Ghana (2003)	5.37	[1.71, 16.83]
Ghana (2008)	4.69	[1.08, 20.42]
Ghana (2014)	3.23	[1.19, 8.79]
Guinea (2005)	1.77	[0.68, 4.57]
Guinea (2018)	1.47	[0.76, 2.85]
Kenya (2003)	2.31	[1.19, 4.47]
Kenya (2008/9)	2.43	[0.89, 6.64]
Kenya (2014)	2.18	[0.88, 5.41]
Kenya (2022)	1.15	[0.44, 3.00]
Lesotho (2009/10)	2.19	[0.86, 5.58]
Liberia (2013)	1.04	[0.38, 2.84]
Liberia (2019/20)	8.30	[2.63, 26.20]
Madagascar (2003/4)	2.30	[0.66, 8.08]
Madagascar (2008/9)	0.85	[0.44, 1.64]
Madagascar (2021)	1.19	[0.58, 2.43]
Malawi (2000)	2.02	[1.06, 3.87]
Malawi (2004/5)	0.91	[0.46, 1.82]
Malawi (2010)	1.76	[1.03, 3.02]
Malawi (2015/16)	2.07	[1.06, 4.06]
Mali (2001)	2.24	[1.25, 4.02]
Mali (2006)	2.23	[1.45, 3.42]
Mali (2012/13)	2.67	[1.55, 4.57]
Mali (2018)	2.48	[1.49, 4.13]
Mozambique (2003/4)	1.81	[0.93, 3.55]
Mozambique (2011)	2.46	[1.31, 4.60]
Namibia (2006/7)	1.25	[0.36, 4.36]
Niger (2006)	0.72	[0.30, 1.76]
Niger (2012)	1.65	[0.82, 3.30]
Nigeria (2008)	2.10	[1.55, 2.86]
Nigeria (2013)	1.71	[1.20, 2.44]
Nigeria (2018)	1.49	[1.13, 1.97]
Rwanda (2000)	1.14	[0.67, 1.95]
Rwanda (2005)	1.58	[0.90, 2.78]
Rwanda (2010/11)	3.18	[1.64, 6.18]
Rwanda (2014/15)	1.77	[0.72, 4.31]
Rwanda (2019/20)	1.49	[0.64, 3.48]
Senegal (2005)	1.87	[1.03, 3.40]
Senegal (2010/11)	1.94	[1.06, 3.56]
Senegal (2015)	0.66	[0.33, 1.34]
Senegal (2016)	1.08	[0.39, 2.96]
Senegal (2018)	1.88	[0.90, 3.93]
Senegal (2019)	0.43	[0.11, 1.68]
Sierra Leone (2008)	3.16	[1.29, 7.73]
Sierra Leone (2013)	1.43	[0.75, 2.74]
Tanzania (2004/5)	2.84	[1.48, 5.47]
Tanzania (2010)	0.97	[0.42, 2.26]
Tanzania (2015/16)	1.47	[0.68, 3.15]
Uganda (2000/1)	1.40	[0.80, 2.46]
Uganda (2006)	1.21	[0.61, 2.40]
Uganda (2011)	2.18	[1.12, 4.25]
Zambia (2007)	1.69	[0.78, 3.65]
Zambia (2013/14)	2.37	[1.30, 4.31]
Zambia (2018/19)	2.22	[1.09, 4.52]
Zimbabwe (2010/11)	1.40	[0.47, 4.16]
Zimbabwe (2015)	2.36	[0.87, 6.40]
**N. Africa/W. Asia/Europe**		
Egypt (2000)	1.57	[0.82, 3.00]
Egypt (2003)	1.14	[0.35, 3.68]
Egypt (2005)	0.82	[0.33, 2.06]
Egypt (2008)	2.06	[0.76, 5.60]
Egypt (2014)	1.07	[0.42, 2.77]
Jordan (2002)	1.17	[0.39, 3.50]
Jordan (2009)	0.93	[0.36, 2.43]
Jordan (2012)	1.25	[0.35, 4.50]
Jordan (2017/18)	1.45	[0.34, 6.28]
Morocco (2003/4)	0.50	[0.21, 1.19]
Turkey (2003/4)	1.04	[0.39, 2.77]
Yemen (2013)	1.28	[0.70, 2.32]
**Central, South & Southeast Asia**		
Bangladesh (2004)	0.90	[0.46, 1.80]
Bangladesh (2007)	0.94	[0.31, 2.83]
Bangladesh (2011)	3.87	[1.50, 10.01]
Bangladesh (2014)	11.56	[2.82, 47.39]
Bangladesh (2017/18)	2.98	[1.18, 7.56]
Cambodia (2010/11)	1.31	[0.43, 3.96]
India (2005/6)	1.71	[1.30, 2.24]
India (2015/16)	1.40	[1.19, 1.65]
India (2019/21)	1.42	[1.19, 1.69]
Indonesia (2002/3)	0.73	[0.26, 2.06]
Indonesia (2007)	2.12	[0.83, 5.40]
Indonesia (2012)	0.66	[0.22, 1.95]
Indonesia (2017)	0.91	[0.28, 2.97]
Myanmar (2015/16)	0.49	[0.10, 2.41]
Nepal (2006)	3.78	[1.53, 9.35]
Nepal (2011)	1.26	[0.43, 3.69]
Nepal (2016/17)	0.72	[0.23, 2.24]
Nepal (2021/22)	3.42	[1.21, 9.65]
Pakistan (2012/13)	2.58	[1.57, 4.25]
Pakistan (2017/18)	2.13	[1.17, 3.89]
Philippines (2003)	0.78	[0.33, 1.85]
Philippines (2022)	1.83	[0.45, 7.34]
Timor-Leste (2009/10)	1.10	[0.55, 2.20]
**Latin America & Caribbean**		
Bolivia (2003/4)	1.45	[0.77, 2.76]
Bolivia (2008)	0.85	[0.34, 2.12]
Colombia (2005)	2.43	[0.82, 7.16]
Dominican Republic (2002)	1.68	[0.74, 3.81]
Guatemala (2014/15)	0.73	[0.29, 1.81]
Honduras (2005/6)	0.91	[0.45, 1.86]
Honduras (2011/12)	1.95	[0.84, 4.53]
Nicaragua (2001)	0.80	[0.28, 2.31]
Peru (2000)	1.67	[0.68, 4.13]
Peru (2007/8)	0.95	[0.26, 3.46]
Peru (2009)	1.38	[0.38, 5.02]

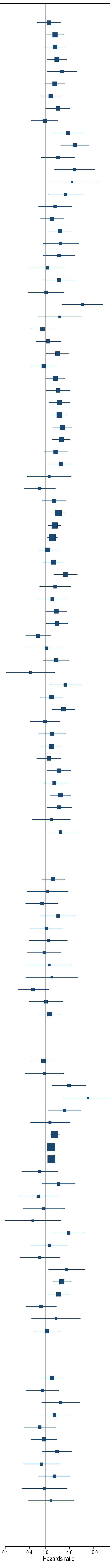

C) IPI 12-17 months versus IPI 18-23 months

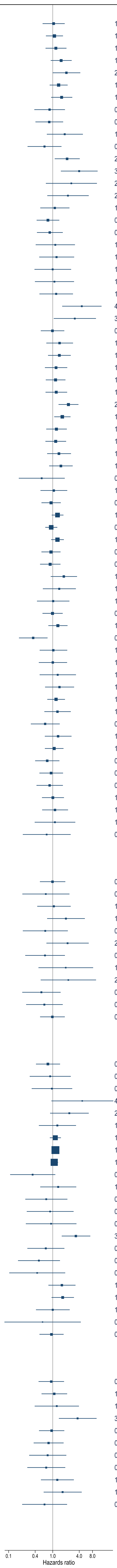

Region / Survey	HR	95% CI
**Sub-Saharan Africa**		
Angola (2015/16)	1.05	[0.60, 1.84]
Benin (2006)	1.09	[0.71, 1.68]
Benin (2011/12)	1.18	[0.70, 2.02]
Benin (2017/18)	1.55	[0.91, 2.64]
Burkina Faso (2003)	2.05	[1.01, 4.14]
Burkina Faso (2010)	1.36	[0.86, 2.14]
Burundi (2010/11)	1.59	[0.93, 2.73]
Ethiopia (2005)	0.85	[0.39, 1.86]
Ethiopia (2011)	0.83	[0.41, 1.69]
Ethiopia (2016)	1.88	[0.74, 4.74]
Gambia (2013)	0.65	[0.27, 1.55]
Gambia (2019/20)	2.13	[1.12, 4.03]
Ghana (2003)	3.98	[1.55, 10.23]
Ghana (2008)	2.64	[0.71, 9.87]
Ghana (2014)	2.22	[0.76, 6.44]
Guinea (2005)	1.12	[0.53, 2.35]
Guinea (2018)	0.78	[0.44, 1.39]
Kenya (2003)	0.86	[0.44, 1.66]
Kenya (2008/9)	1.14	[0.42, 3.14]
Kenya (2014)	1.23	[0.50, 3.01]
Kenya (2022)	1.00	[0.39, 2.56]
Lesotho (2009/10)	1.09	[0.40, 2.99]
Liberia (2013)	1.20	[0.51, 2.83]
Liberia (2019/20)	4.57	[1.66, 12.58]
Madagascar (2003/4)	3.17	[1.07, 9.39]
Madagascar (2008/9)	0.99	[0.54, 1.80]
Madagascar (2021)	1.43	[0.73, 2.83]
Malawi (2000)	1.41	[0.79, 2.52]
Malawi (2004/5)	1.19	[0.67, 2.10]
Malawi (2010)	1.16	[0.71, 1.91]
Malawi (2015/16)	1.20	[0.70, 2.09]
Mali (2001)	2.27	[1.37, 3.76]
Mali (2006)	1.65	[1.09, 2.48]
Mali (2012/13)	1.22	[0.72, 2.05]
Mali (2018)	1.17	[0.69, 1.98]
Mozambique (2003/4)	1.39	[0.76, 2.55]
Mozambique (2011)	1.53	[0.84, 2.79]
Namibia (2006/7)	0.56	[0.17, 1.84]
Niger (2006)	1.06	[0.53, 2.09]
Niger (2012)	0.92	[0.56, 1.49]
Nigeria (2008)	1.28	[0.96, 1.72]
Nigeria (2013)	0.93	[0.69, 1.25]
Nigeria (2018)	1.28	[0.94, 1.76]
Rwanda (2000)	0.91	[0.56, 1.48]
Rwanda (2005)	0.88	[0.52, 1.47]
Rwanda (2010/11)	1.79	[0.91, 3.49]
Rwanda (2014/15)	1.41	[0.61, 3.29]
Rwanda (2019/20)	1.02	[0.45, 2.35]
Senegal (2005)	0.99	[0.59, 1.65]
Senegal (2010/11)	1.31	[0.81, 2.13]
Senegal (2015)	0.36	[0.17, 0.75]
Senegal (2016)	1.04	[0.51, 2.09]
Senegal (2018)	1.01	[0.49, 2.08]
Senegal (2019)	1.30	[0.51, 3.30]
Sierra Leone (2008)	1.43	[0.68, 3.00]
Sierra Leone (2013)	1.19	[0.76, 1.86]
Tanzania (2004/5)	1.28	[0.65, 2.52]
Tanzania (2010)	0.68	[0.32, 1.41]
Tanzania (2015/16)	1.32	[0.67, 2.62]
Uganda (2000/1)	1.08	[0.67, 1.72]
Uganda (2006)	0.75	[0.40, 1.39]
Uganda (2011)	0.92	[0.51, 1.68]
Zambia (2007)	0.85	[0.43, 1.67]
Zambia (2013/14)	1.01	[0.58, 1.77]
Zambia (2018/19)	1.13	[0.58, 2.18]
Zimbabwe (2010/11)	1.12	[0.40, 3.19]
Zimbabwe (2015)	0.73	[0.21, 2.51]
**N. Africa/W. Asia/Europe**		
Egypt (2000)	0.99	[0.52, 1.90]
Egypt (2003)	0.70	[0.21, 2.35]
Egypt (2005)	1.06	[0.45, 2.51]
Egypt (2008)	1.99	[0.76, 5.25]
Egypt (2014)	0.68	[0.21, 2.16]
Jordan (2002)	2.17	[0.73, 6.44]
Jordan (2009)	0.67	[0.24, 1.86]
Jordan (2012)	1.97	[0.48, 8.10]
Jordan (2017/18)	2.26	[0.54, 9.43]
Morocco (2003/4)	0.55	[0.21, 1.48]
Turkey (2003/4)	0.65	[0.25, 1.66]
Yemen (2013)	0.98	[0.52, 1.84]
**Central, South & Southeast Asia**		
Bangladesh (2004)	0.78	[0.42, 1.44]
Bangladesh (2007)	0.88	[0.30, 2.52]
Bangladesh (2011)	0.96	[0.34, 2.72]
Bangladesh (2014)	4.68	[0.94, 23.23]
Bangladesh (2017/18)	2.39	[0.88, 6.44]
Cambodia (2010/11)	1.27	[0.49, 3.31]
India (2005/6)	1.15	[0.87, 1.51]
India (2015/16)	1.15	[0.98, 1.35]
India (2019/21)	1.09	[0.91, 1.30]
Indonesia (2002/3)	0.35	[0.11, 1.12]
Indonesia (2007)	1.33	[0.53, 3.35]
Indonesia (2012)	0.71	[0.24, 2.11]
Indonesia (2017)	0.87	[0.26, 2.92]
Myanmar (2015/16)	0.92	[0.25, 3.39]
Nepal (2006)	3.36	[1.62, 6.98]
Nepal (2011)	0.70	[0.27, 1.81]
Nepal (2016/17)	0.49	[0.17, 1.43]
Nepal (2021/22)	0.44	[0.10, 1.88]
Pakistan (2012/13)	1.62	[0.81, 3.23]
Pakistan (2017/18)	1.68	[0.95, 2.98]
Philippines (2003)	1.00	[0.42, 2.38]
Philippines (2022)	0.59	[0.08, 4.26]
Timor-Leste (2009/10)	0.94	[0.51, 1.73]
**Latin America & Caribbean**		
Bolivia (2003/4)	0.93	[0.48, 1.77]
Bolivia (2008)	1.09	[0.57, 2.09]
Colombia (2005)	1.24	[0.40, 3.86]
Dominican Republic (2002)	3.68	[1.39, 9.72]
Guatemala (2014/15)	0.94	[0.49, 1.80]
Honduras (2005/6)	0.81	[0.37, 1.73]
Honduras (2011/12)	0.77	[0.30, 2.00]
Nicaragua (2001)	0.71	[0.27, 1.89]
Peru (2000)	1.27	[0.55, 2.96]
Peru (2007/8)	1.68	[0.63, 4.46]
Peru (2009)	0.65	[0.21, 2.08]

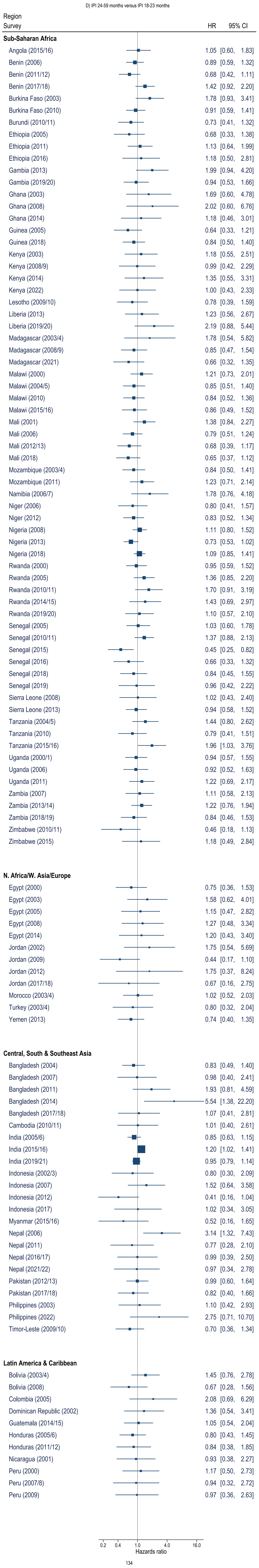
D) IPI 24-59 months versus IPI 18-23 months
Region
Survey
HR
95% CI
Sub-Saharan Africa
Angola (2015/16) 1.05 [0.60, 1.83]
Benin (2006) 0.89 [0.59, 1.32]
Benin (2011/12) 0.68 [0.42, 1.11]
Benin (2017/18) 1.42 [0.92, 2.20]
Burkina Faso (2003) 1.78 [0.93, 3.41]
Burkina Faso (2010) 0.91 [0.59, 1.41]
Burundi (2010/11) 0.73 [0.41, 1.32]
Ethiopia (2005) 0.68 [0.33, 1.38]
Ethiopia (2011) 1.13 [0.64, 1.99]
Ethiopia (2016) 1.18 [0.50, 2.81]
Gambia (2013) 1.99 [0.94, 4.20]
Gambia (2019/20) 0.94 [0.53, 1.66]
Ghana (2003) 1.69 [0.60, 4.78]
Ghana (2008) 2.02 [0.60, 6.76]
Ghana (2014) 1.18 [0.46, 3.01]
Guinea (2005) 0.64 [0.33, 1.21]
Guinea (2018) 0.84 [0.50, 1.40]
Kenya (2003) 1.18 [0.55, 2.51]
Kenya (2008/9) 0.99 [0.42, 2.29]
Kenya (2014) 1.35 [0.55, 3.31]
Kenya (2022) 1.00 [0.43, 2.33]
Lesotho (2009/10) 0.78 [0.39, 1.59]
Liberia (2013) 1.23 [0.56, 2.67]
Liberia (2019/20) 2.19 [0.88, 5.44]
Madagascar (2003/4) 1.78 [0.54, 5.82]
Madagascar (2008/9) 0.85 [0.47, 1.54]
Madagascar (2021) 0.66 [0.32, 1.35]
Malawi (2000) 1.21 [0.73, 2.01]
Malawi (2004/5) 0.85 [0.51, 1.40]
Malawi (2010) 0.84 [0.52, 1.36]
Malawi (2015/16) 0.86 [0.49, 1.52]
Mali (2001) 1.38 [0.84, 2.27]
Mali (2006) 0.79 [0.51, 1.24]
Mali (2012/13) 0.68 [0.39, 1.17]
Mali (2018) 0.65 [0.37, 1.12]
Mozambique (2003/4) 0.84 [0.50, 1.41]
Mozambique (2011) 1.23 [0.71, 2.14]
Namibia (2006/7) 1.78 [0.76, 4.18]
Niger (2006) 0.80 [0.41, 1.57]
Niger (2012) 0.83 [0.52, 1.34]
Nigeria (2008) 1.11 [0.80, 1.52]
Nigeria (2013) 0.73 [0.53, 1.02]
Nigeria (2018) 1.09 [0.85, 1.41]
Rwanda (2000) 0.95 [0.59, 1.52]
Rwanda (2005) 1.36 [0.85, 2.20]
Rwanda (2010/11) 1.70 [0.91, 3.19]
Rwanda (2014/15) 1.43 [0.69, 2.97]
Rwanda (2019/20) 1.10 [0.57, 2.10]
Senegal (2005) 1.03 [0.60, 1.78]
Senegal (2010/11) 1.37 [0.88, 2.13]
Senegal (2015) 0.45 [0.25, 0.82]
Senegal (2016) 0.66 [0.33, 1.32]
Senegal (2018) 0.84 [0.45, 1.55]
Senegal (2019) 0.96 [0.42, 2.22]
Sierra Leone (2008) 1.02 [0.43, 2.40]
Sierra Leone (2013) 0.94 [0.58, 1.52]
Tanzania (2004/5) 1.44 [0.80, 2.62]
Tanzania (2010) 0.79 [0.41, 1.51]
Tanzania (2015/16) 1.96 [1.03, 3.76]
Uganda (2000/1) 0.94 [0.57, 1.55]
Uganda (2006) 0.92 [0.52, 1.63]
Uganda (2011) 1.22 [0.69, 2.17]
Zambia (2007) 1.11 [0.58, 2.13]
Zambia (2013/14) 1.22 [0.76, 1.94]
Zambia (2018/19) 0.84 [0.46, 1.53]
Zimbabwe (2010/11) 0.46 [0.18, 1.13]
Zimbabwe (2015) 1.18 [0.49, 2.84]
N. Africa/W. Asia/Europe
Egypt (2000) 0.75 [0.36, 1.53]
Egypt (2003) 1.58 [0.62, 4.01]
Egypt (2005) 1.15 [0.47, 2.82]
Egypt (2008) 1.27 [0.48, 3.34]
Egypt (2014) 1.20 [0.43, 3.40]
Jordan (2002) 1.75 [0.54, 5.69]
Jordan (2009) 0.44 [0.17, 1.10]
Jordan (2012) 1.75 [0.37, 8.24]
Jordan (2017/18) 0.67 [0.16, 2.75]
Morocco (2003/4) 1.02 [0.52, 2.03]
Turkey (2003/4) 0.80 [0.32, 2.04]
Yemen (2013) 0.74 [0.40, 1.35]
Central, South & Southeast Asia
Bangladesh (2004) 0.83 [0.49, 1.40]
Bangladesh (2007) 0.98 [0.40, 2.41]
Bangladesh (2011) 1.93 [0.81, 4.59]
Bangladesh (2014) 5.54 [1.38, 22.20]
Bangladesh (2017/18) 1.07 [0.41, 2.81]
Cambodia (2010/11) 1.01 [0.40, 2.61]
India (2005/6) 0.85 [0.63, 1.15]
India (2015/16) 1.20 [1.02, 1.41]
India (2019/21) 0.95 [0.79, 1.14]
Indonesia (2002/3) 0.80 [0.30, 2.09]
Indonesia (2007) 1.52 [0.64, 3.58]
Indonesia (2012) 0.41 [0.16, 1.04]
Indonesia (2017) 1.02 [0.34, 3.05]
Myanmar (2015/16) 0.52 [0.16, 1.65]
Nepal (2006) 3.14 [1.32, 7.43]
Nepal (2011) 0.77 [0.28, 2.10]
Nepal (2016/17) 0.99 [0.39, 2.50]
Nepal (2021/22) 0.97 [0.34, 2.78]
Pakistan (2012/13) 0.99 [0.60, 1.64]
Pakistan (2017/18) 0.82 [0.40, 1.66]
Philippines (2003) 1.10 [0.42, 2.93]
Philippines (2022) 2.24 [0.71, 10.70]
Timor-Leste (2009/10) 0.70 [0.36, 1.34]
Latin America & Caribbean
Bolivia (2003/4) 1.45 [0.76, 2.78]
Bolivia (2008) 0.67 [0.28, 1.56]
Colombia (2005) 2.08 [0.69, 6.29]
Dominican Republic (2002) 1.36 [0.54, 3.41]
Guatemala (2014/15) 1.05 [0.54, 2.04]
Honduras (2005/6) 0.80 [0.43, 1.45]
Honduras (2011/12) 0.84 [0.38, 1.85]
Nicaragua (2001) 0.93 [0.38, 2.27]
Peru (2000) 1.17 [0.50, 2.73]
Peru (2007/8) 0.94 [0.32, 2.72]
Peru (2009) 0.97 [0.36, 2.63]
0.2
0.4
1.0
4.0
16.0
Hazards ratio

E-Table 6: Tests of proportional-hazards assumption

Region<br>Survey	Chis-square	P-value
**sub-Saharan Africa**		
Angola (2015/16)	0.8	0.381
Benin (2006)	0.6	0.456
Benin (2011/12)	1.4	0.237
Benin (2017/18)	1.4	0.241
Burkina Faso (2003)	0.9	0.352
Burkina Faso (2010)	0.3	0.602
Burundi (2010/11)	0.1	0.768
Ethiopia (2005)	0.2	0.623
Ethiopia (2011)	0.0	0.932
Ethiopia (2016)	0.1	0.788
Gambia (2013)	0.0	0.970
Gambia (2019/20)	2.9	0.089
Ghana (2003)	0.0	0.894
Ghana (2008)	0.3	0.603
Ghana (2014)	0.0	0.951
Guinea (2005)	0.4	0.533
Guinea (2018)	1.1	0.292
Kenya (2003)	0.1	0.762
Kenya (2008/9)	1.3	0.255
Kenya (2014)	0.7	0.399
Kenya (2022)	0.1	0.744
Lesotho (2009/10)	0.4	0.515
Liberia (2013)	0.6	0.437
Liberia (2019/20)	0.0	0.826
Madagascar (2003/4)	1.4	0.238
Madagascar (2008/9)	1.4	0.234
Madagascar (2021)	0.9	0.354
Malawi (2000)	0.0	0.992
Malawi (2004/5)	1.2	0.274
Malawi (2010)	0.1	0.744
Malawi (2015/16)	0.0	0.890
Mali (2001)	0.3	0.590
Mali (2006)	0.1	0.796
Mali (2012/13)	0.0	0.982
Mali (2018)	0.0	0.858
Mozambique (2003/4)	2.0	0.161
Mozambique (2011)	2.3	0.128
Namibia (2006/7)	1.3	0.258
Niger (2006)	0.6	0.443
Niger (2012)	2.1	0.149
Nigeria (2008)	2.5	0.111
Nigeria (2013)	0.3	0.587
Nigeria (2018)	0.0	0.842

Rwanda (2000)	0.0	0.899
Rwanda (2005)	1.0	0.316
Rwanda (2010/11)	0.2	0.617
Rwanda (2014/15)	0.4	0.511
Rwanda (2019/20)	1.2	0.276
Senegal (2005)	3.0	0.083
Senegal (2010/11)	0.8	0.379
Senegal (2015)	0.2	0.669
Senegal (2016)	0.0	0.860
Senegal (2018)	0.7	0.393
Senegal (2019)	0.6	0.424
Sierra Leone (2008)	0.2	0.619
Sierra Leone (2013)	0.0	0.901
Tanzania (2004/5)	0.1	0.793
Tanzania (2010)	5.3	0.021
Tanzania (2015/16)	6.5	0.011
Uganda (2000/1)	0.4	0.534
Uganda (2006)	2.0	0.159
Uganda (2011)	0.6	0.437
Zambia (2007)	1.9	0.168
Zambia (2013/14)	1.7	0.187
Zambia (2018/19)	0.1	0.710
Zimbabwe (2010/11)	0.9	0.345
Zimbabwe (2015)	0.0	0.845
**North Africa, Western Asia & Europe**		
Egypt (2000)	0.1	0.717
Egypt (2003)	2.5	0.113
Egypt (2005)	0.2	0.698
Egypt (2008)	0.9	0.349
Egypt (2014)	3.6	0.058
Jordan (2002)	1.3	0.258
Jordan (2009)	0.0	0.993
Jordan (2012)	0.4	0.529
Jordan (2017/18)	6.1	0.014
Morocco (2003/4)	5.6	0.017
Türkiye (2003/4)	0.7	0.390
Yemen (2013)	0.6	0.452
**Central, South & Southeast Asia**		
Bangladesh (2004)	2.0	0.153
Bangladesh (2007)	1.0	0.329
Bangladesh (2011)	0.0	0.828
Bangladesh (2014)	3.1	0.076
Bangladesh (2017/18)	0.1	0.714
Cambodia (2010/11)	0.1	0.811
India (2005/6)	0.0	0.881
India (2015/16)	0.4	0.525
India (2019/21)	5.8	0.016
Indonesia (2002/3)	7.9	0.005
Indonesia (2007)	1.4	0.240
Indonesia (2012)	4.2	0.040

Indonesia (2017)	1.4	0.245
Myanmar (2015/16)	0.9	0.342
Nepal (2006)	0.3	0.569
Nepal (2011)	1.8	0.185
Nepal (2016/17)	0.4	0.552
Nepal (2021/22)	0.1	0.744
Pakistan (2012/13)	4.1	0.042
Pakistan (2017/18)	0.7	0.399
Philippines (2003)	1.2	0.271
Philippines (2022)	0.3	0.602
Timor-Leste (2009/10)	0.5	0.480
**Latin America & Caribbean**		
Bolivia (2003/4)	0.1	0.726
Bolivia (2008)	0.2	0.639
Colombia (2005)	0.2	0.635
Dominican Republic (2002)	4.6	0.031
Guatemala (2014/15)	0.0	0.890
Honduras (2005/6)	0.1	0.819
Honduras (2011/12)	4.5	0.035
Nicaragua (2001)	0.0	0.945
Peru (2000)	2.3	0.132
Peru (2007/8)	1.6	0.211
Peru (2009)	1.8	0.178

The 10 surveys highlighted in red were excluded for the sensitivity analysis as the hazards-proportional assumption did not hold, p-value <0.05.

E-Table 7: The pooled hazard ratios (HRs) of perinatal mortality by IPI preceded by livebirth for surveys that met the proportionality assumption

	Pooled	95%CI		
**Inter-pregnancy interval**				
<6 months	**2.74**	**(2.53**	-	**2.97)**
6-11 months	**1.68**	**(1.55**	-	**1.81)**
12-17 months	**1.16**	**(1.10**	-	**1.23)**
18-23 months	1.00			
24-59 months	1.01	(0.95	-	1.07)

Based on 103 surveys.

E-Figure 3: Survey-specific hazard ratios of perinatal mortality, by IPI (months) following Stillbirth

A) IPI of less than 3 months versus IPI 3+ months

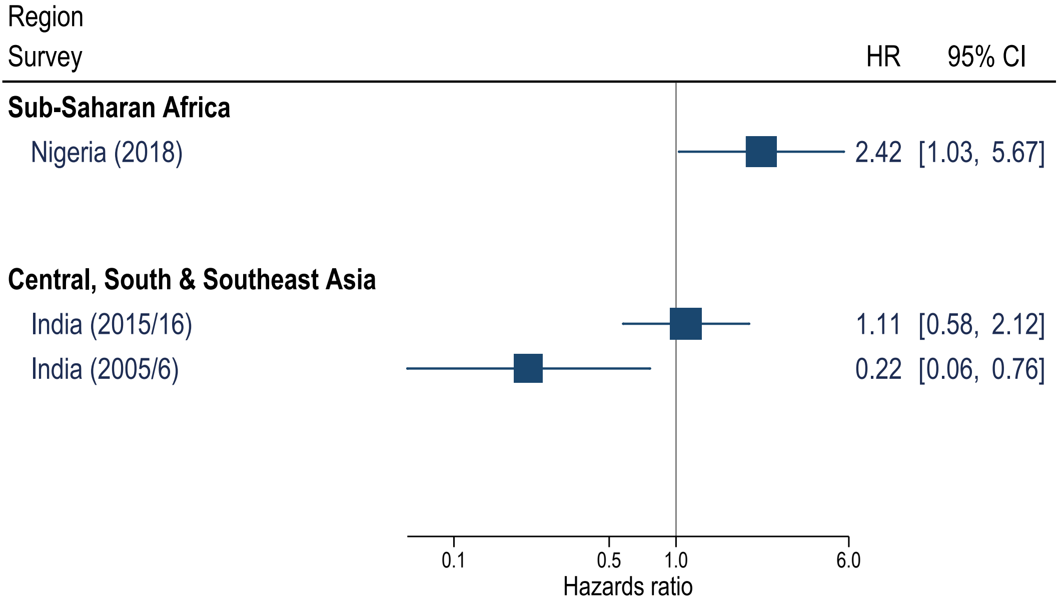

B) IPI of less than 6 months versus IPI 6+ months

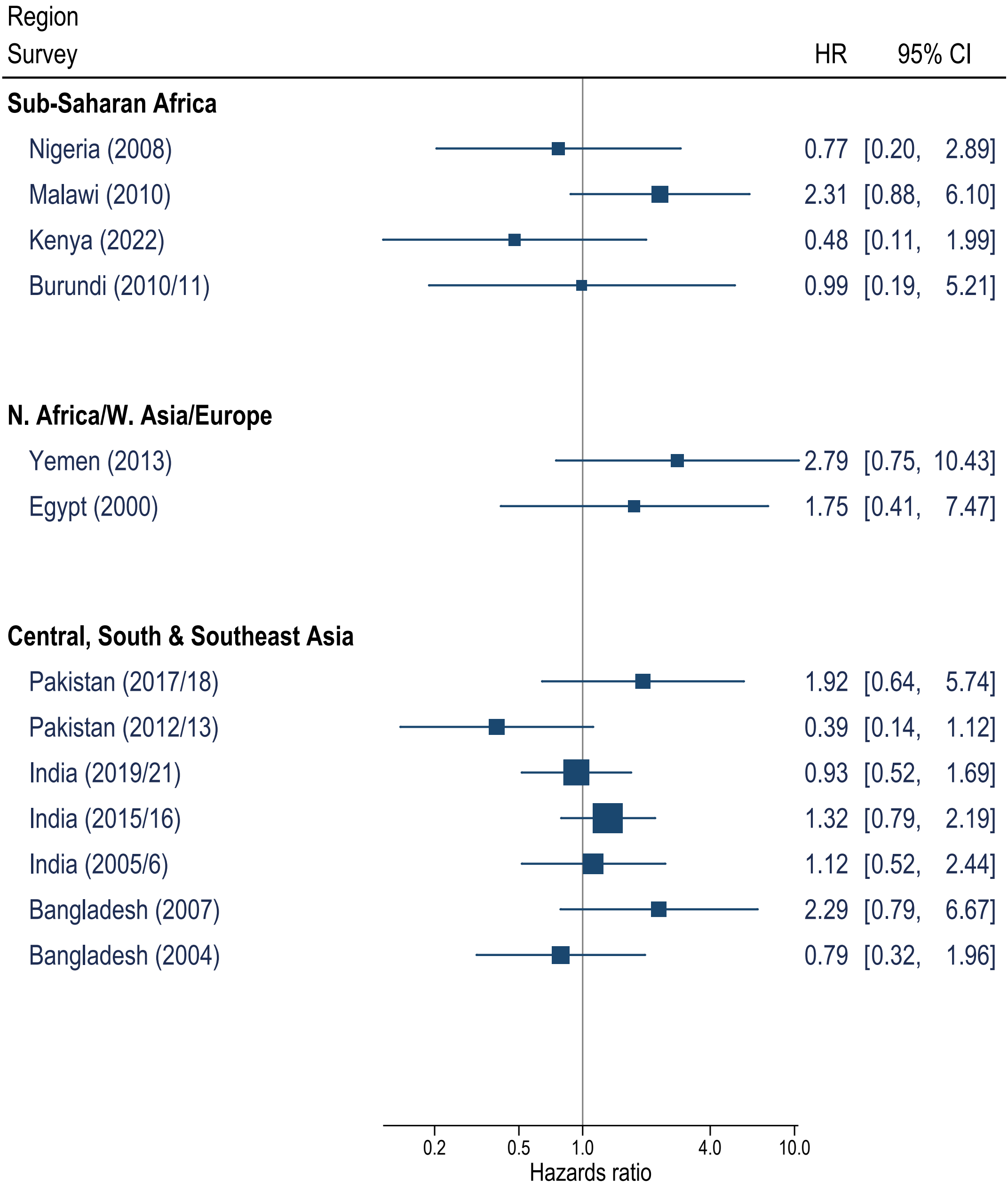

C) IPI of less than 12 months versus IPI 12+ months

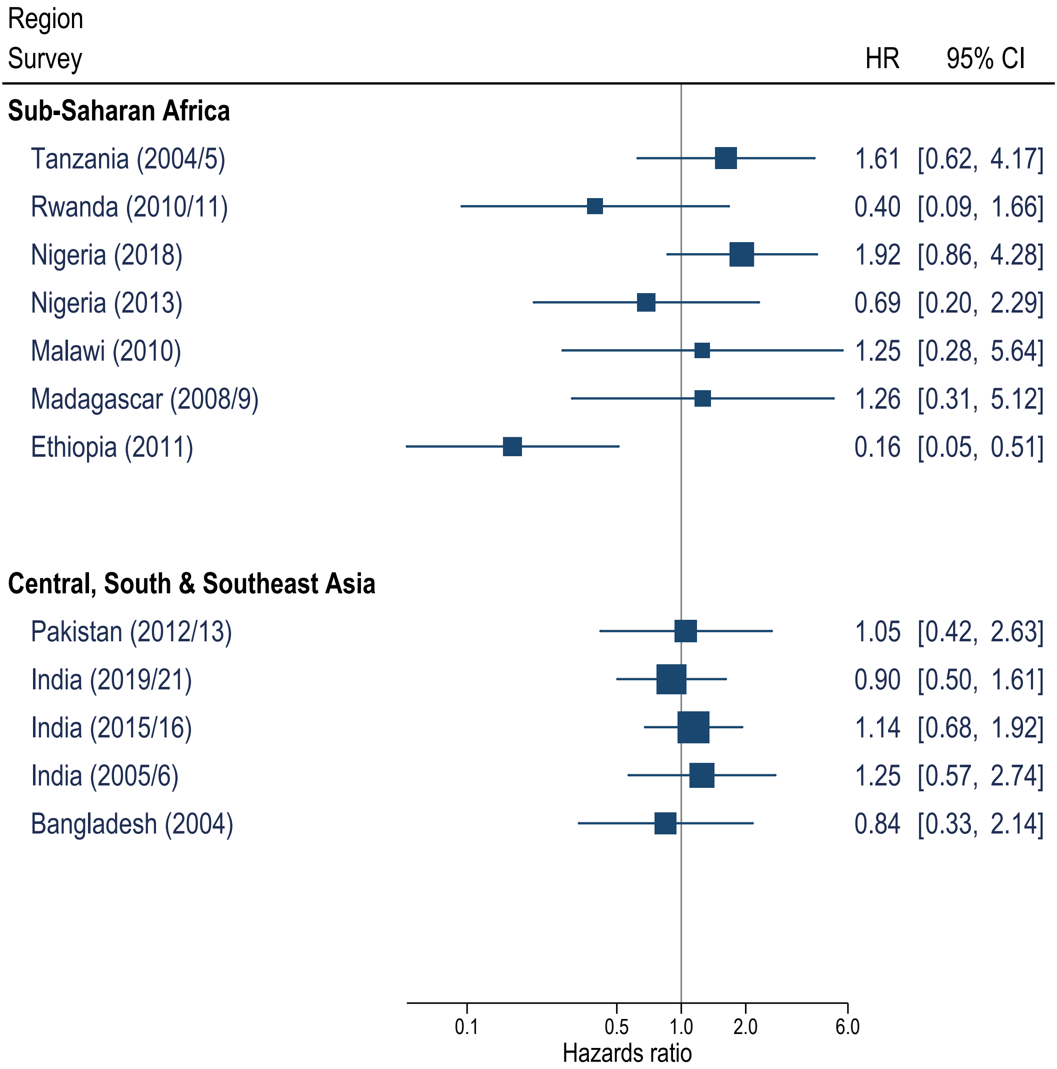

E-Figure 4: Survey-specific hazard ratios of perinatal mortality, by IPI (months) following abortion

A) IPI of less than 3 months versus IPI 3+ months

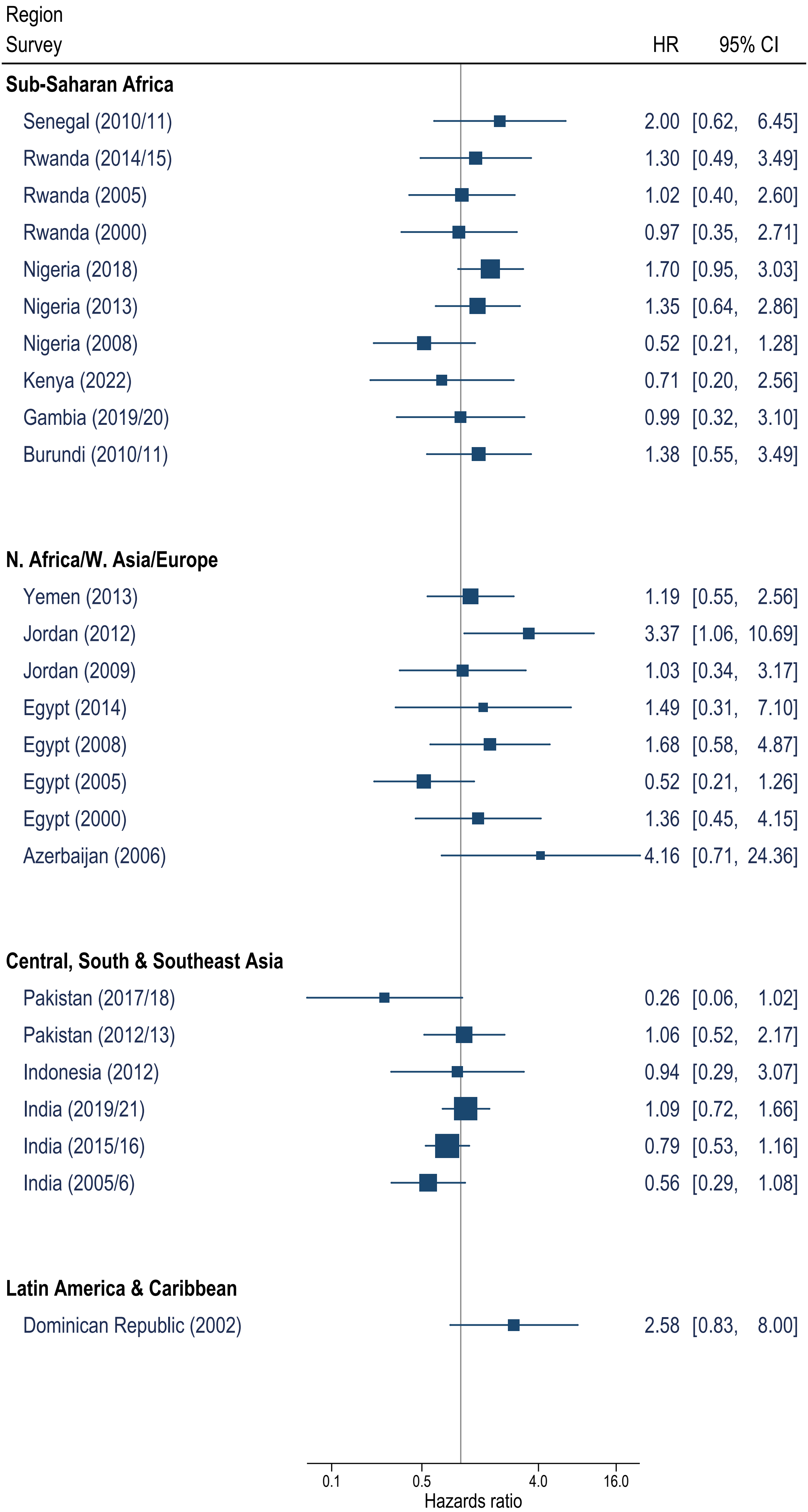

B) IPI of less than 6 months versus IPI 6+ months

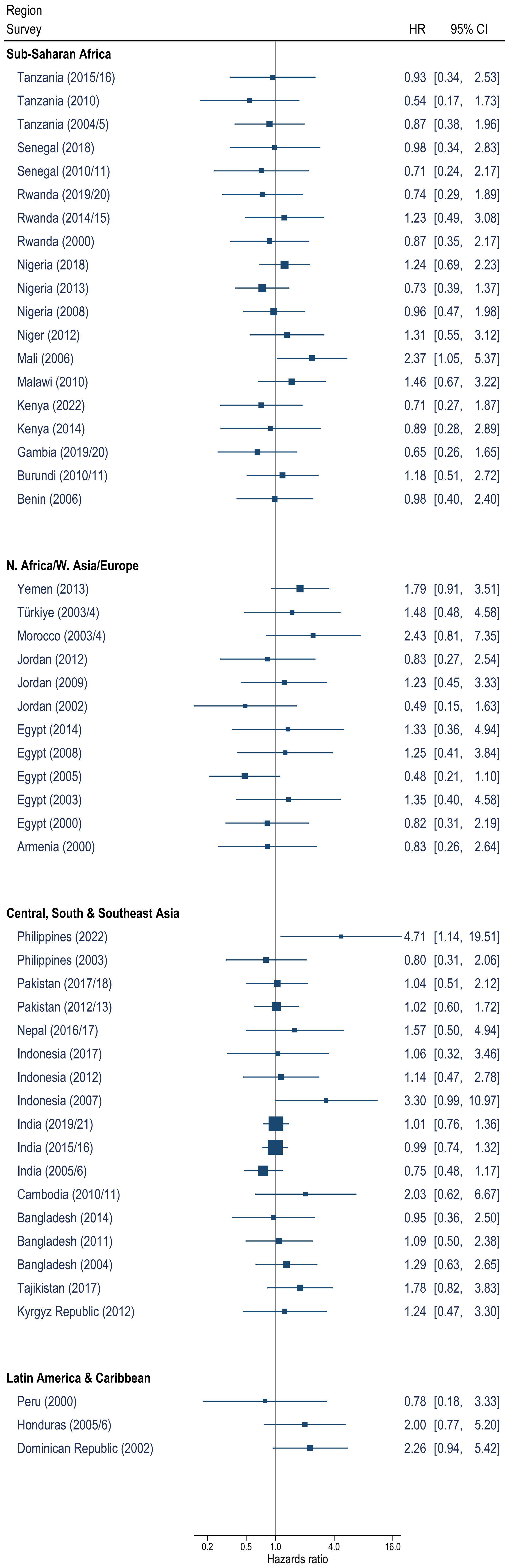

C) IPI of less than 12 months versus IPI 12+ months

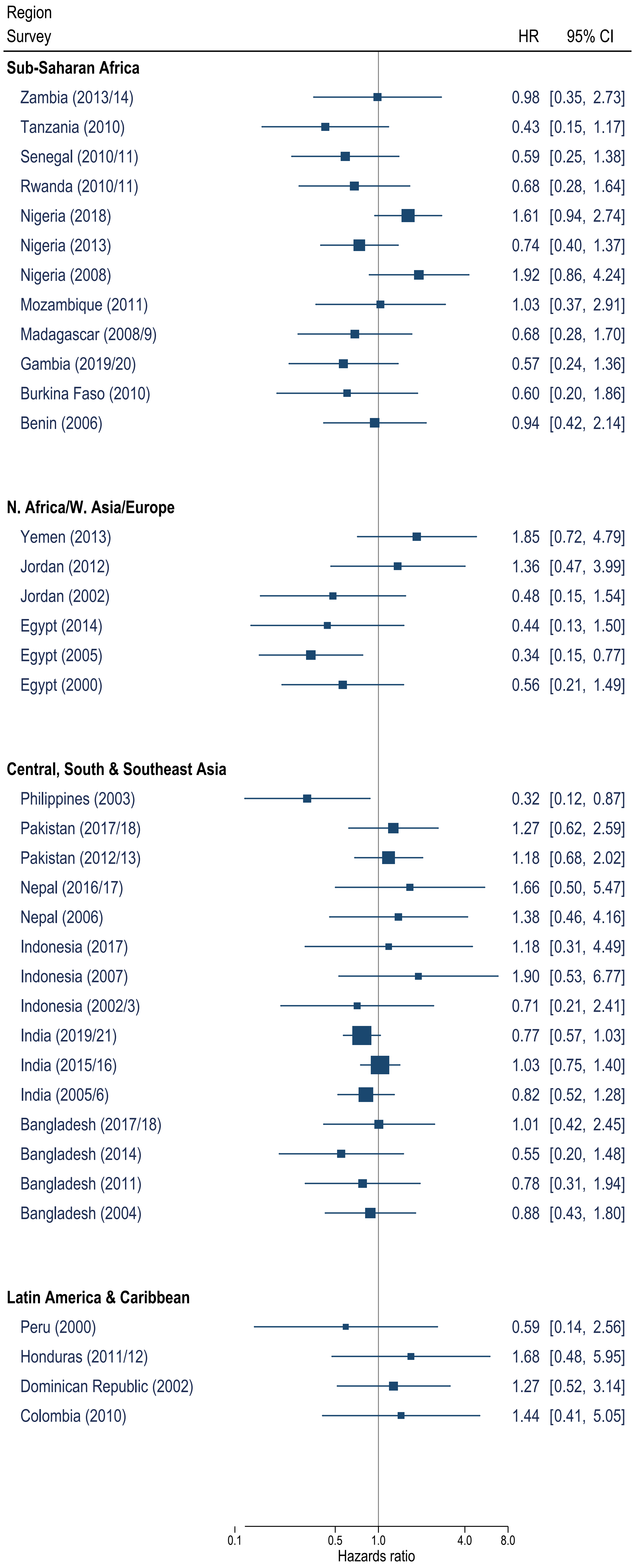

E-Figure 5: Correlation of median IPI with contraceptive failure rate, at survey level for the most recent DHS surveys

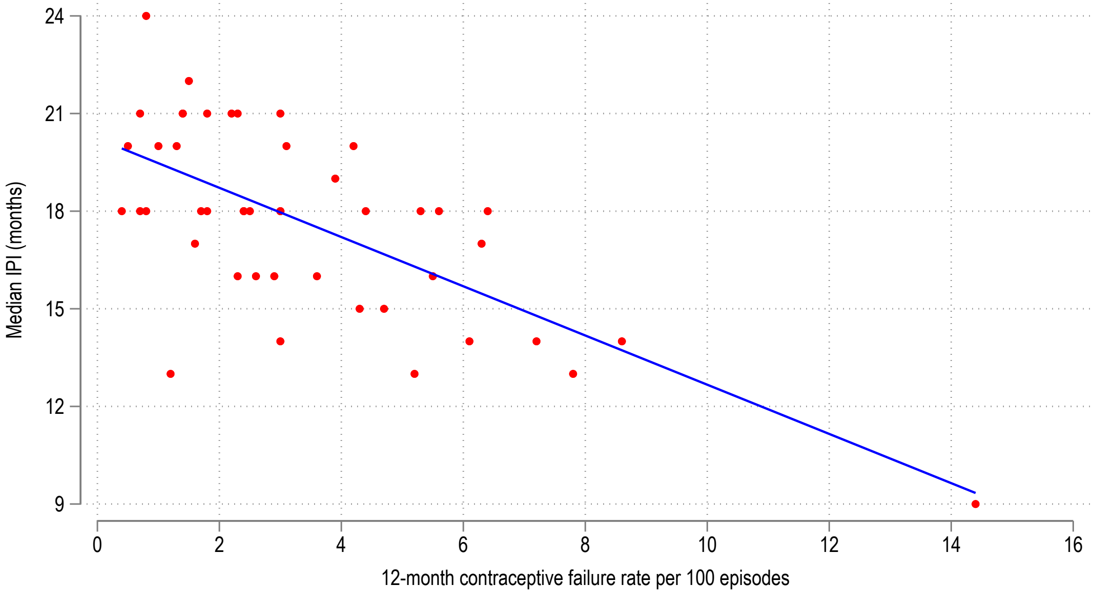

Supplement: Supplementary appendix [file mmc1.pdf]
